# Hallucinating symmetric protein assemblies

**DOI:** 10.1126/science.add1964

**Published:** 2022-09-15

**Authors:** B. I. M. Wicky, L. F. Milles, A. Courbet, R. J. Ragotte, J. Dauparas, E. Kinfu, S. Tipps, R. D. Kibler, M. Baek, F. DiMaio, X. Li, L. Carter, A. Kang, H. Nguyen, A. K. Bera, D. Baker

**Affiliations:** 1 Department of Biochemistry, University of Washington, Seattle, WA, USA.; 2 Institute for Protein Design, University of Washington, Seattle, WA, USA.; 3 Howard Hughes Medical Institute, University of Washington, Seattle, WA, USA.

## Abstract

Deep learning generative approaches provide an opportunity to broadly explore protein structure space beyond the sequences and structures of natural proteins. Here we use deep network hallucination to generate a wide range of symmetric protein homo-oligomers given only a specification of the number of protomers and the protomer length. Crystal structures of 7 designs are very close to the computational models (median RMSD: 0.6 Å), as are 3 cryoEM structures of giant 10 nanometer rings with up to 1550 residues and C33 symmetry; all differ considerably from previously solved structures. Our results highlight the rich diversity of new protein structures that can be generated using deep learning, and pave the way for the design of increasingly complex components for nanomachines and biomaterials.

Cyclic protein oligomers play key roles in almost all biological processes and constitute nearly 30% of all deposited structures in the Protein Data Bank (PDB, (1) (2–4)). Because of the many applications of cyclic protein oligomers, ranging from small molecule binding and catalysis to building blocks for nanocage assemblies (5), *de novo* design of such structures has been of considerable interest from the beginning of the protein design field (6, 7). While there have been a number of successes (8–10), current approaches typically require specification of the structure of the protomers in advance. With the exception of parametrically designed structures (11, 12), design strategies involve rigid body docking of characterized monomers into higher order symmetric structures, followed by interface optimization to generate low-energy assembled states (13–17). The requirement that the protomer structure be specified in advance has limited the exploration of the full space of oligomeric structures, such as assemblies with more intertwined chains. For monomeric protein design, broad exploration of the space of possible structures has become possible by deep network hallucination: starting from a random amino acid sequence, Markov chain Monte Carlo (MCMC) optimization favoring folding to a well-defined state converges on new sequences that fold to new structures (18–21). By extension, we reasoned that deep network hallucination could enable the design of higher-order protein assemblies in one step, without prespecification or experimental confirmation of the structures of the protomers, provided that a suitable loss function specifying both protomer folding and assembly could be formulated (18–20, 22–25).

We set out to broadly explore the space of cyclic protein homo-oligomers by developing a method for hallucinating such structures that places no constraints on the structures of either the protomers or the overall assemblies. Starting from only a choice of chain length *L* and oligomer valency *N* (2 for a dimer, 3 for a trimer, etc.), the method carries out a Monte Carlo search in sequence space starting from a random sequence (Fig. 1A). The loss function guiding the search is computed by inputting *N* copies of the sequence into the AlphaFold2 (AF2) network (26), and combining structure prediction confidence metrics (pLDDT; per-residue structural accuracy (27), and pTM; an estimate of the TM-score (28)) with a measure of cyclic symmetry (the standard deviation of the distances between the center of mass of adjacent protomers within the predicted structure).

We found that monomers and dimeric to heptameric assemblies could readily be generated by this procedure for chains of 65 to 130 amino acids, with converging trajectories typically coalescing to cyclic homo-oligomeric structures within a few hundred steps (approximately 1 to 7 days of CPU-time for monomers to heptamers respectively, Fig. S1–2). The resulting structures are topologically diverse, spanning all-α, mixed α/β and all-β structures, and differ from the structures of cyclic *de novo* designs present in the PDB (Fig. 1B). These assemblies, which we term HALs, also differ from natural proteins in both structure (Fig. 1C) and sequence (Fig. 1D), with the median closest relatives in the PDB having TM-scores of 0.67 and 0.57 for the protomers and oligomers respectively (29% of the structures have TM-scores < 0.5, the cutoff for fold assignment in CATH/SCOP (29)), indicating considerable generalization beyond the PDB training set.

We selected 150 designs with AF2 pLDDT > 0.7 and pTM > 0.7 for experimental testing. However, virtually none showed significant soluble expression when produced in *E. coli* (median soluble yield: 9 mg per liter of culture-equivalent, Fig. S3), and of the few that were marginally soluble none had both the expected oligomerization state by size-exclusion chromatography (SEC), and a circular dichroism (CD) profile consistent with the hallucinated structure. We speculated that this failure could be a consequence of over-fitting during MCMC optimization leading to the generation of adversarial sequences, i.e. confidently-predicted sequences with unrealistic biophysical properties (Fig. S4–5). Adversarial samples have been generated by activation maximization in the context of image classification neural networks, which similarly leads to unrealistic outputs (30–32). To eliminate such over-fitting, we generated new sequences for the HAL backbones using the recently developed ProteinMPNN sequence design neural network (*accompanying manuscript: Dauparas et al*.). For each original backbone, 24 to 48 sequences were generated with ProteinMPNN, and assembly to the target oligomeric structure validated with AF2 (these dozens of evaluations compared to the hundreds performed during hallucination make overfitting much less likely). In addition, we independently evaluated the sequences using an updated version of RoseTTAFold (RF2) (33), and found that RF2 did not confidently predict the structure of most of the original AF2 hallucinated sequences, but successfully predicted almost all ProteinMPNN sequences (Fig. S4, S6–7).

We tested 96 ProteinMPNN-designed HALs with pLDDT > 0.75 and root-mean-square deviation (RMSD) to original backbone < 1.5 Å and found that 71/96 (74%) were expressed to high levels (median yield: 247 mg per liter of culture-equivalent), 50/96 (52%) had a SEC retention volume consistent with the size of the oligomer (of which 30 (60%) were monodisperse) (Fig. 1F and Fig. S8–9), and at least 21/96 (22%) had the correct oligomeric state when assessed by SEC-Multi Angle Light Scattering (SEC-MALS) (Fig. 1G and Fig. S10). CD analysis of the soluble samples indicated that 67/71 (96%) had secondary structure contents consistent with the designs (Fig. S9). These success rates are in stark contrast to those of the original AF2 hallucinated sequences, indicating that the MCMC procedure generates viable backbones with over-fitted sequences exhibiting various pathologies (Fig. S5), and highlights the power of ProteinMPNN to generate sequences which fold to a given backbone structure (Fig. 1E). We assessed the thermal stability of the 71 soluble HALs by CD spectroscopy, and found that 54 maintained their secondary structure up to 95 °C (Fig. S9). SEC characterization of the heated-treated samples indicated that most designs retained their oligomeric state, suggesting that ProteinMPNN-designed HALs are thermostable (Fig. 1H, S9).

To evaluate design accuracy we attempted crystallization of 19 designs and succeeded in solving crystal structures for seven (three C2s, two C3s and two C4s, Fig. 2). All crystal structures had the correct oligomerization state and closely matched the design models (median Cα RMSD of 0.6 Å across all designs, with resolutions ranging from 1.8 to 3.4 Å, Fig. S11, Table S1). The side chain conformations in the crystal structures also closely match those of the design models (Fig. 2).

The solved structures exhibit striking diversity with many intricate structural features. HALC2_062 (Fig. 2A) is a three-layer homo-dimer with a single helix from each protomer packed together between two outer β-sheets (one from each protomer), while HALC2_065 (Fig. 2B) is also a mixed α/β homo-dimer, but has a single, continuous β-sheet shared between both chains, which wraps around two perpendicular paired helices. These two hallucinated structures are distinct from any structure in the PDB, with TM-scores to their best matches of 0.59 and 0.54 respectively (Fig. 4A–B, Table S2). HALC2_068 (Fig. 2C) is a fully helical dimer with an extensive interface formed by 6 interacting helices (3 from each protomer), with a single perpendicular helix buttressing the interfacial helices. Despite the low secondary structure complexity and absence of long-range contacts, this design also differs significantly from its closest structural relative in the PDB (TM-score: 0.57, Fig. 4C, Table S2). HALC3_104 (Fig. 2D) is a homo-trimeric coiled-coil, with a central bundle of three helices, augmented by an outer-ring of three shorter helices that lie in the groove formed by adjacent protomer (the closest matching structure in the PDB has a TM-score of 0.88, Fig. 4D, Table S2). HALC3_109 (Fig. 2E) is a homo-trimeric three-layer all-helical structure, with three inner helices splaying outwards to contact two additional helices from the same protomers at angles of roughly 25° and 90°; the closest assembly in the PDB has a TM-score of 0.69 (Fig. 4E, Table S2). HALC4_135 (Fig. 2F) is a coiled-coil composed of helical hairpins reminiscent of HALC3_104, but with C4 symmetry instead of C3, and a discontinuous superhelical twist. Despite its simple topology, the closest structural homologue to this design has a TM-score of only 0.59 (Fig. 4F, Table S2). HALC4_136 (Fig. 2G) is composed of 3-helix protomers with eight outer helices encasing four almost fully hydrophobic inner helices, where two of the helices are rigidly linked through a 90° helical kink. The closest match in the PDB has a TM-score of 0.71, but the matched structure has C5 symmetry rather than the C4 symmetry of the design and crystal structure (Fig. 4G, Table S2).

Next, we sought to generate HALs of greater complexities across longer length-scales by extending the design specifications to structures of higher symmetry (up to C42) and longer oligomeric assembly sequence lengths (up to 1800 residues). To generate multiple possible oligomers from a single structure, we specified the MCMC trajectories as single-chains with internal sequence symmetry; the resulting structure-symmetric repeat proteins can be split into any desired oligomeric assembly compatible with factorization (e.g. C15 into a pentamer, shorthanded as C15–5). To maximize the exploration of the design space while minimizing the use of computational resources, we devised an evolution-based computational strategy: many short MCMC trajectories (< 50 steps) outputs were clustered by structure prediction confidence metrics (pLDDT and pTM), and then used to seed new trajectories (see Supplementary Materials). Using this approach, we hallucinated cyclic homo-oligomers from C5 to C42 with their largest dimension ranging from 7 to 14 nm (median: 10 nm), which were then divided into homo-trimers, tetramers, pentamers, hexamers, heptamers, octamers, and dodecamer, and the backbones were re-designed with ProteinMPNN (Fig 1C). While the α/β topology of some of these larger HALs is reminiscent of natural Leucine Rich Repeats (LRRs, (34)), which is reflected by a median highest protomer TM-scores of 0.64, these ring-shaped structures differ considerably from the horseshoe folds of LRRs that do not close into cyclic structures. The closest oligomer structures in the PDB have a median TM-score of 0.47, and BLAST sequence similarity searches for the repetitive sequence motif do not return any significant hits (Fig. 1D); the hallucination process as in the earlier cases generalizes beyond the training set.

These larger HALs have overall molecular weights greater than 100 kDa, and thus were well-suited for structural characterization by electron microscopy (EM). We screened soluble large HALs with a SEC retention volume consistent with the size of their oligomeric state by negative stain EM (nsEM), and in most cases observed monodisperse particles of the expected size and circular shape. We obtained 2D class averages and 3D *ab initio* reconstructed electron density maps for six designs with C6 to C42 internal repeat symmetry (factorized as: two C5s, three C6s, and one C7) that clearly showed low-resolution structural features and diameters consistent with their designs (Fig. 3A, Fig. S12). We selected three designs: one C15 homo-pentamer (HALC5–15_262), one C18 homo-hexamer (HALC6–18_265) and one C33 homo-trimer (HALC3–33_343) for high-resolution single particle cryoEM characterization. We collected datasets that produced 2D class averages with clear secondary structure feature placements, and 3D *ab initio* reconstruction and refinement yielded 3D electron density maps at 4.38 Å, 6.51 Å and 6.32 Å resolution respectively (Fig. 3B, Fig. S13–16). HALC5–15_262 was originally designed as a homo-hexamer, but structure prediction calculations were more consistent with a pentameric structure of nearly identical protomer conformation and only a very slightly shifted subunit interface (Fig. S17); the cryoEM structure is also a pentamer with an Cα RMSD of 1.69 Å to this predicted structure (Fig. S16).

These hallucinated rings are giant structures quite unlike anything in the PDB. The three rings solved by cryoEM, HALC5–15_262, HALC6–18_265 and HALC3–33_343, are 87 Å, 99 Å and 100 Å in diameter and 40 to 50 Å high, with a continuous parallel β-sheet in the lumen of the pore, and outer helices that enforce the curvature and closure of the ring. HALC3–33_343 has a simple helix-loop-sheet structural motif as its repeating unit, while in HALC5–15_262 and HALC6–18_265, the repeating unit contains two distinct helix-loop-sheet elements, which produces an alternating helical outer pattern clearly observable in the 2D class averages. While both structures have matches to LRRs for their protomers (TM-score of 0.65 for both, but to different structures), the oligomeric assemblies are strikingly different from any natural protein (TM-scores of 0.48 and 0.49 respectively, Fig. 4H–I, Table S2). HALC3–33_343 has an unusual internal loop region breaking the outer helices midway in the repeat, producing a widening of the ring on one side, which is clearly visible in the cryoEM reconstruction; the protomer has a low TM-score (0.48) despite having an LRR-like topology, and the oligomer is even further from anything currently known (TM-score: 0.41, Fig. 4J, Table S2) The high structural symmetry of these designed complexes rivals that of natural proteins: the highest cyclic symmetry recorded in the PDB for naturally occurring proteins is C39 (Vault proteins (35), PDB 4HL8 and 7PKY), and there are no closed symmetric α/β ring-like structures.

## Conclusion

**Fig. 1.**
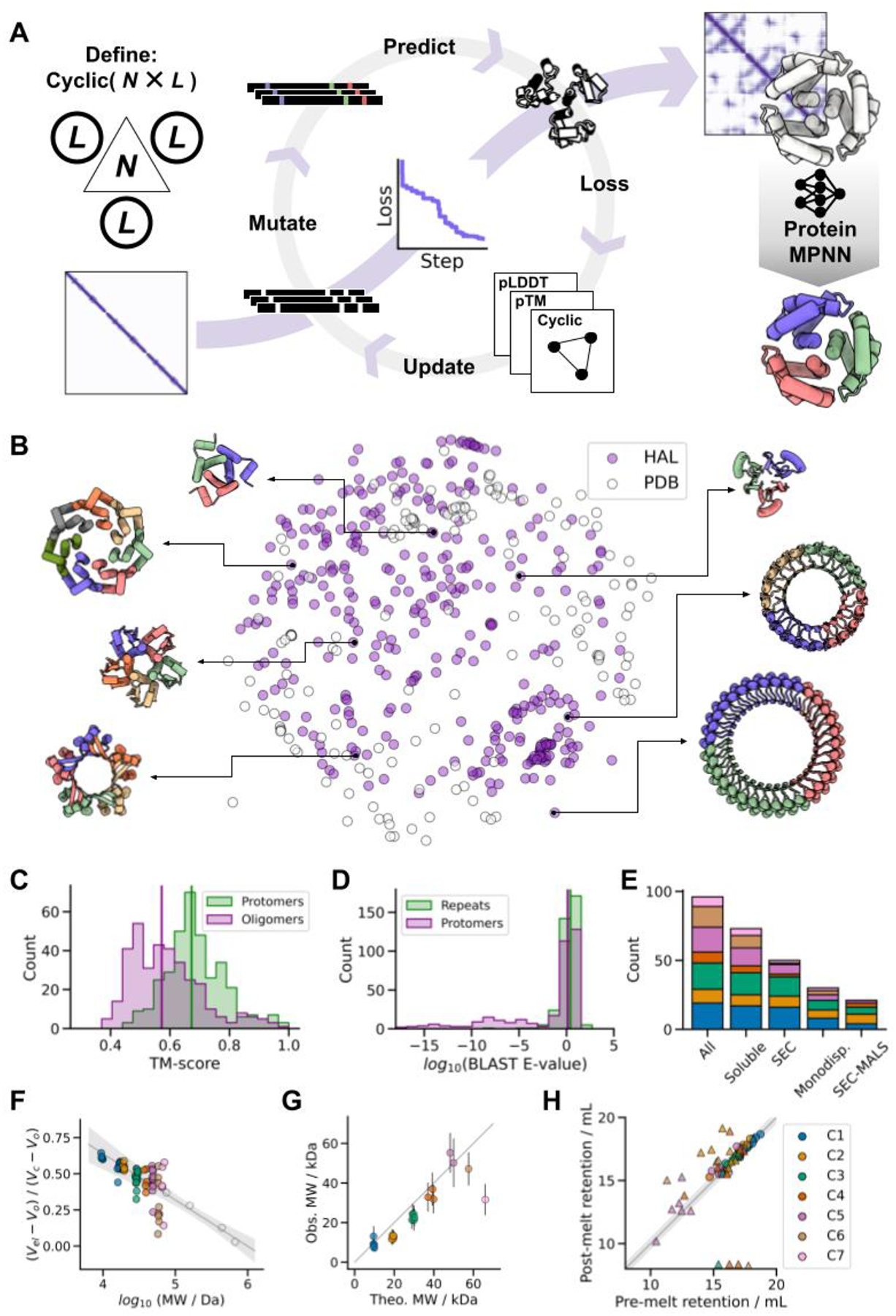
Hallucinating symmetric protein assemblies (**A**) Starting from choice of a cyclic symmetry and protein length, a random sequence is optimized by MCMC through the AF2 network until the resulting structure fits the design objective, followed by sequence re-design with ProteinMPNN. (**B**) The method generates structurally diverse outputs, quantified here by multi-dimensional scaling of protomer pairwise structural similarities between experimentally tested HALs (*N* = 351) and all *de novo* cyclic oligomers present in the PDB (*N* = 162). (**C**) Generated structures differ from those in the PDB. Median TM-scores to the closest match: 0.67 and 0.57 for the protomers and oligomers respectively (vertical lines). (**D**) Generated sequences are unrelated to naturally-occuring proteins. Median BLAST E-values from the closet hit in UniRef100: 2.6 and 1.3 for the repeat motifs and protomers respectively (vertical lines). (**E**) Success counts of ProteinMPNN-designed HALs at different levels of characterization. (**F**) Most soluble HALs have SEC retention volumes consistent with their oligomeric state. The gray line shows the fit to calibration standards (open circles), and the shaded area represents the 95% confidence interval of the calibration. (**G**) The observed molecular weights of HALs from SEC-MALS are close to those computed from the design models. (**H**) ProteinMPNN-designed HALs are thermostable. Pre-melting and post-melting retention volumes are closely correlated; circles represent designs that remained monodisperse, while triangles indicate polydispersity after heat-treatment. In plots **E**-**H**, the data is categorized by cyclic symmetry classes. The legend is shown in **H**.

Our deep learning-based approach to designing cyclic homo-oligomers jointly generates protomers and their oligomeric assemblies without the need for a hierarchical docking approach. We report a rich assortment of *de novo* protein homo-oligomers across the nanoscopic scale, with broad topological diversity while maintaining design constraints such as symmetry and oligomeric state. These hallucinated oligomers differ substantially from natural oligomers in both sequence (median lowest BLAST E-value against UniRef100 of 1.3 for the repeated sequence motifs, Fig. 1D, Table S3)) and structure (median best TM-score between biounits from the PDB and HALS of 0.57, Fig. 1C, Table S2); our computational pipeline interpolates and extends native fold-space rather than simply recapitulating memorized protein structures, demonstrating the power of deep learning to explore previously uncharted regions of the design landscape (Fig. 1B). Our results also highlight the power of the ProteinMPNN method for protein sequence design; of the 30 out of the 192 designs evaluated experimentally by either SEC-MALS, nsEM, cryoEM, or X-ray crystallography, 27 had the intended oligomeric state, and 7 out of 19 for which crystallization was attempted formed diffracting crystals (this is a considerably higher crystallization success rate than typical for Rosetta *de novo* designs, and suggests that ProteinMPNN may generate protein surfaces more likely to form crystal contacts). More generally, our results show that a rich diversity of protein structures and assemblies beyond what exists in the PDB can now be accessed by deep learning-based generative models.

The formalism described here can be extended to other types of complex design tasks, including the design of higher order point group symmetries, arbitrary symmetric or asymmetric hetero-oligomeric assemblies, oligomeric scaffolding of existing functional domains, and design of multiple states, provided a loss function describing the solution can be formalized and computed. Computational requirements and hardware memory limitations become bottlenecks for hallucination of increasingly large structures; the development of computationally less expensive structure prediction methods with fewer parameters, as well as generative approaches such as diffusion models ((36, 37)) which more directly sample in structure space, should enable the design of even more complex protein structures and assemblies.

## Supplementary Material

SI

## Figures and Tables

**Fig. 2.**
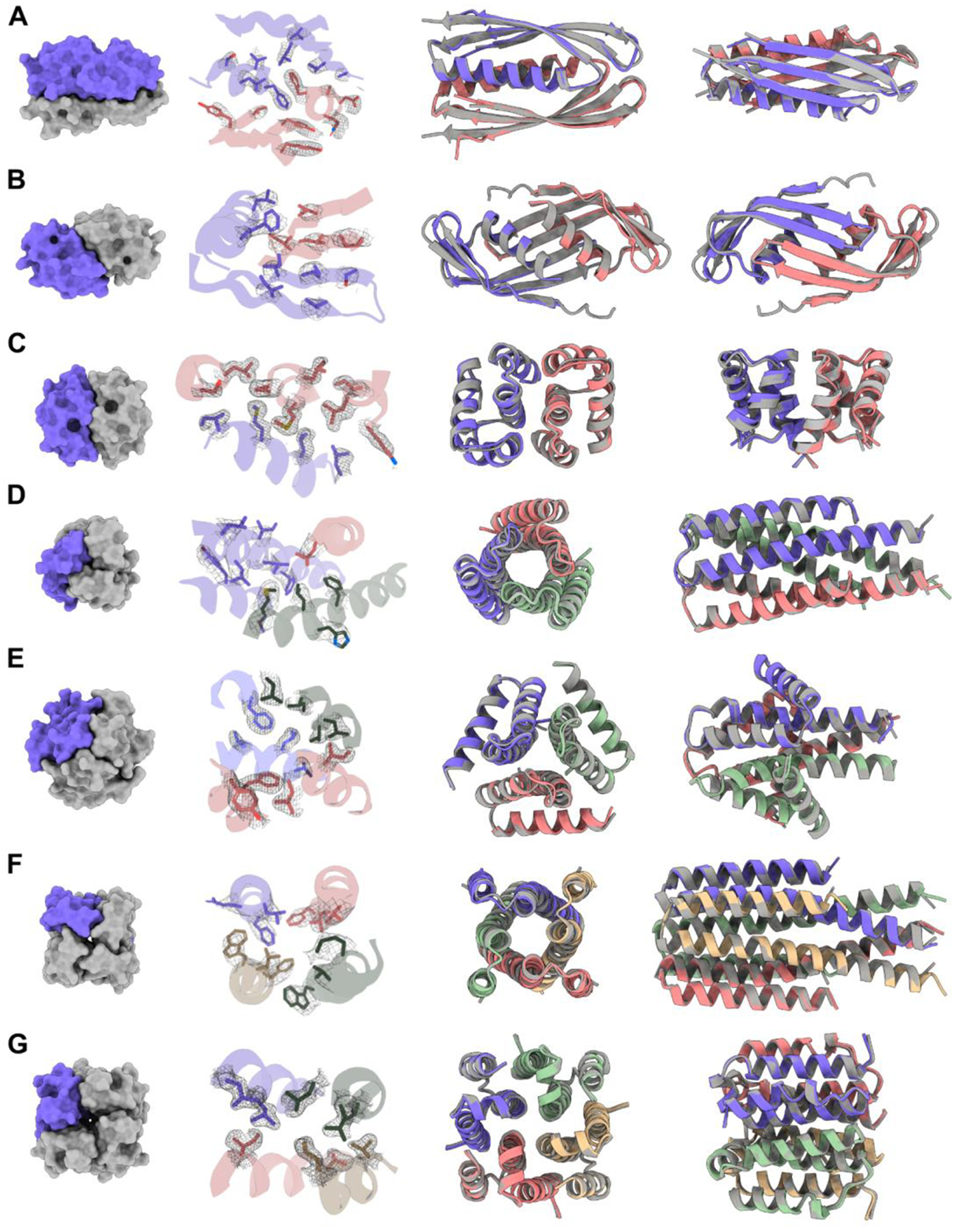
Structures of HALs solved by X-ray crystallography compared to their design models. (**A**) HALC2_062 (RMSD: 0.81 Å). (**B**) HALC2_065 (RMSD: 1.02 Å). (**C**) HALC2_068 (RMSD: 0.86 Å). (**D**) HALC3_104 (RMSD: 0.42 Å). (**E**) HALC3_109 (RMSD: 0.46 Å). (**F**) HALC4_135 (RMSD: 0.60 Å). (**G**) HALC4_136 (RMSD: 0.34 Å). For each row, the first panel shows a surface rendering of the oligomer with one protomer highlighted in purple, the second highlights the side-chain rotamers of the design model to the 2mFo-DFc map (in gray), and the last two panels show two different orientations of the structural overlays between the model (gray) and the solved structure (colored by chains).

**Fig. 3.**
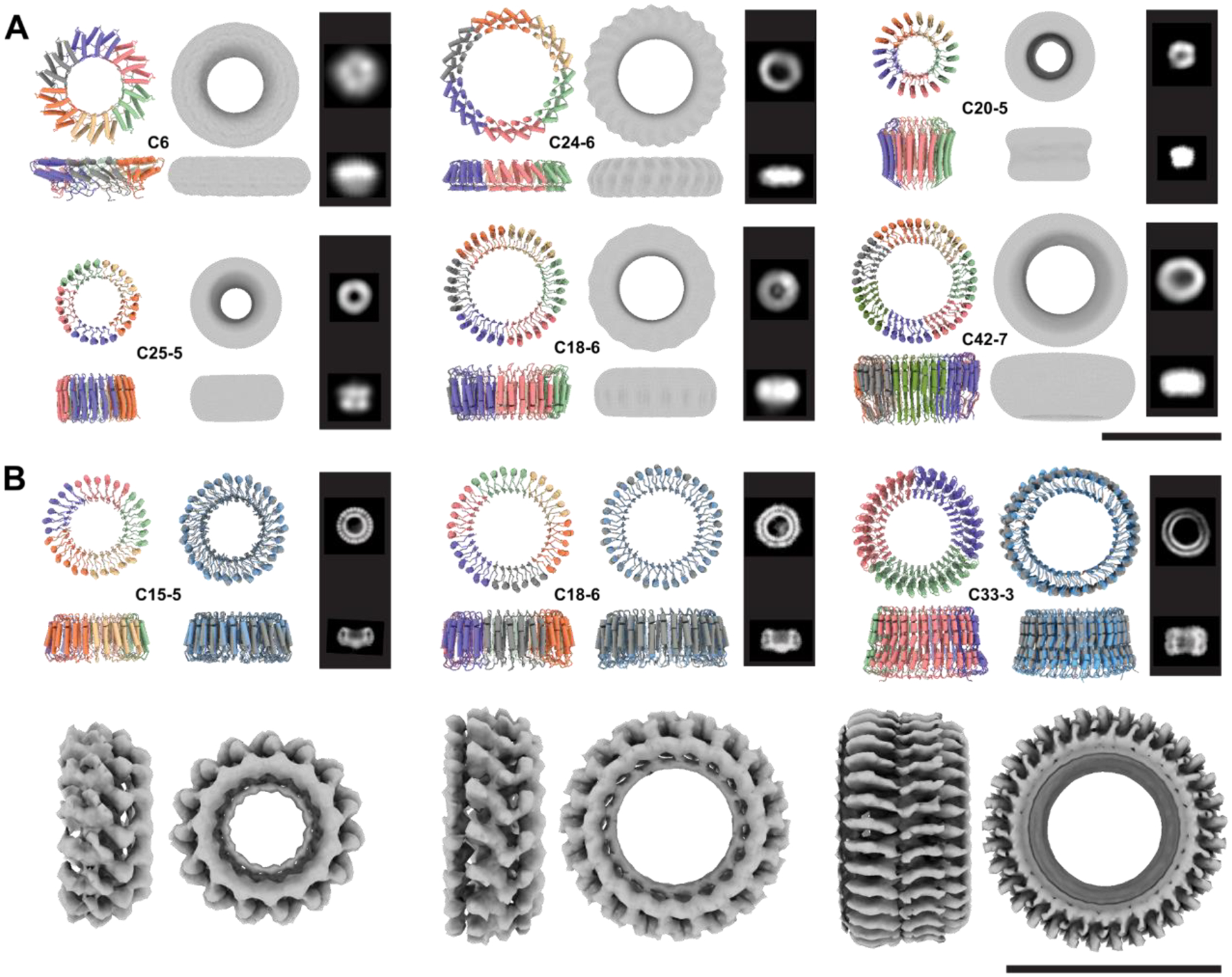
Cryo-electron and negative stain electron microscopy validation of large HALs. For each design, the model is shown colored by chain and the corresponding internal symmetry (X) and oligomerization state (Y) are indicated (CX-Y). The electron density map is shown next to the model alongside characteristic 2D class averages. (**A**) Negative stain characterization of HALs. Ring diameters are 92 Å, 110 Å, 75 Å, 80 Å, 100 Å, 107 Å, for HALC6_220, HALC24–6_316, HALC20–5_308, HALC25–5_341, HALC18–6_278 and HALC42–7_351, respectively. (**B**) CryoEM characterisation of three large HALs. The ring diameters are 87 Å, 99 Å, and 100 Å for HALC15–5_262, HALC18–6_265, and HALC33–3_343, respectively. Top row left panels: design model colored by chain; Top row, right panels: superpositions of the CryoEM model (gray) and design model (blue). The computed backbone atom RMSD between the designed and experimental structure are 0.81 Å, 1.69 Å, and 2.30 Å respectively (Fig. S16). Bottom row: 4.38 Å, 6.51 Å, and 6.32 Å cryoEM electron density maps. Scale bars = 10 nm.

**Fig. 4.**
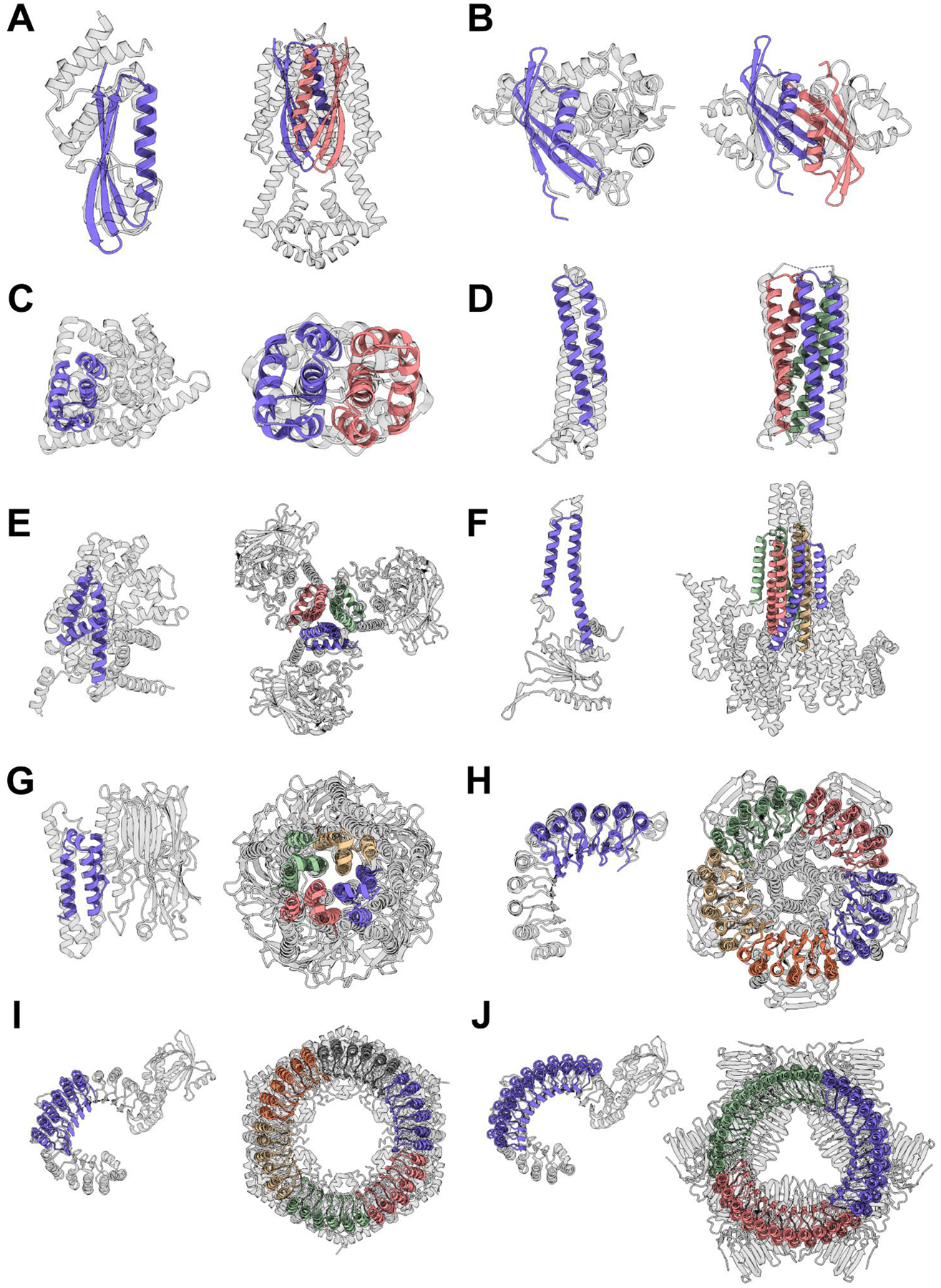
Hallucinated structures differ significantly from their closest matches in the PDB. For each structure solved by crystallography (Fig. 2) or cryoEM (Fig. 3B), the closest structural match to the protomer and to the oligomer are shown on the left and right respectively. Designs are colored by chain and the closest matching PDB is shown in gray. In most cases the closest oligomer has an entirely different structure; this is particularly evident for the larger designs in **G**-**H**. TM-scores (protomer | oligomer) are indicated in parentheses, and the PDB IDs are reported in Table S2. (**A**) HALC2_062 (0.69 | 0.59). (**B**) HALC2_065 (0.67 | 0.54). (**C**) HALC2_068 (0.67 | 0.57). (**D**) HALC3_104 (0.87 | 0.88). (**E**) HALC3_109 (0.78 | 0.69). (**F**) HALC4_135 (0.80 | 0.59). (**G**) HALC4_136 (0.80 | 0.71). (**H**) HALC15–5_262 (0.65 | 0.46). (**I**) HALC18–6_265 (0.65 | 0.49). (**J**) HALC33–3_343 (0.49 | 0.41).

## Data Availability

All data is available in the main text or as supplementary materials. Scripts and computational methods are available on GitHub (https://github.com/bwicky/oligomer_hallucination), Crystallographic datasets have been deposited in the PDB (accession codes: 8D03, 8D04, 8D05, 8D06, 8D07, 8D08 8D09). EM maps have been deposited in the EMDB (accession codes: EMD-27658, EMD-27659, EMD-27660).
