# Supplementary material for: Hallucinating symmetric protein assemblies
Source: Science. Author manuscript; Available in PMC 2022 Dec 6. (PMC9724707; doi:10.1126/science.add1964)
Supplement: SI [file NIHMS1843544-supplement-SI.docx]

Supplementary Materials for

**Hallucinating symmetric protein assemblies**

B. I. M. Wicky^1,2^†, L. F. Milles^1,2^†, A. Courbet^1,2,3^†, R. J. Ragotte^1,2^, J. Dauparas^1,2^, E. Kinfu^1,2^, S. Tipps^1,2^, R. D. Kibler^1,2^, M. Baek^1,2^, F. DiMaio^1,2^, X. Li^1,2^, L. Carter^1,2^, A. Kang^1,2^, H. Nguyen^1,2^, A. K. Bera^1,2^, D. Baker^1,2,3^

^1^ Department of Biochemistry, University of Washington, Seattle, WA, USA.

^2^ Institute for Protein Design, University of Washington, Seattle, WA, USA.

^3^ Howard Hughes Medical Institute, University of Washington, Seattle, WA, USA.

† These authors contributed equally to this work.

Corresponding author: David Baker, dabaker@uw.edu

**This PDF file includes:**

Materials and Methods

Figs. S1 to S17

Tables S1 to S4

Supplementary references

**Other Supplementary Materials for this manuscript include:**

Data frame containing all protein information and experimental data

Oligomer hallucination code

Design models

## **Materials and Methods**

### Computational design strategy

We reasoned that the ability of AF2 to predict oligomers could be employed to design such structures using a MCMC search in sequence space combined with a suitable loss function. The advantage of such a method is its ability to jointly optimize the protomer and oligomer structures, without putting any constraints on the nature of the protomer itself (e.g. the requirement to adopt a well-folded structure in isolation as is typically the case for docking approaches). We employed simplifications during AF2 predictions to reduce computational cost, and defined a composite loss function composed of structure quality terms and a geometric term.

MCMC trajectories were initialized with a random protomer sequence of specified length, with the composition of amino acids respecting the BLOSUM62 background frequencies. Cysteines were disallowed for all hallucinations. Protomers sequences were concatenated to generate oligomeric assemblies during AF2 prediction: chain breaks in the concatenated protomer sequences were specified by re-indexing residues after the break with a 200 increment, resulting in AF2 predicting them as separate chains. To reduce computational costs the number of recycles was set to 1, the number of ensembles was also set to 1, and AMBER relax was not performed. After each prediction, losses were computed on the AF2 prediction confidence metrics (pLDDT, pTM, pAE) as well as the coordinates of the predicted structure.

Mean AF2 pLDDT and AF2 pTM scale between 0 and 1, where higher values are better, thus the loss (by definition the objective to minimize) was calculated for each as one minus their respective values. For enforcing cyclic symmetry we computed a cyclic loss term defined as the standard deviation between the center of mass of adjacent protomers (computed on Cα). Minimizing this value enforces cyclic symmetry.

The loss functions computed to generate all cyclic oligomers <= C7 was:

Dual_cyclic: loss = 1 - 0.5*(AF2pTM +AF2 pLDDT) + standard deviation(center of masses)

After an initial prediction, mutations were introduced in the protomer sequences (tied positions), and the structure re-predicted. Positions with low pLDDT values (lowest half) were targeted, and mutations were chosen based on the BLOSUM62 substitution frequencies. The number of mutations at each step was linearly decayed over the course of the trajectory starting from 3 per protomer down to 1.

Simulated annealing was employed during optimization, with the starting temperature set to 0.01 and the half-life of the exponential decay set to 500 steps. Mutations were accepted or rejected according to the Metropolis criterion.

All parameters and loss functions mentioned above and several others are available in the code repository. We highlight that the command to generate e.g. a homo-trimer is simply:

./oligomer_hallucination.py --L 65 --oligo AAA+ --loss dual_cyclic --out C3_oligomer

Modest computational means were sufficient to hallucinate assemblies up to C7 with protomer lengths of 65 amino acids. The largest C7 assemblies required a week on a single CPU with 6 GB of memory to generate 300 steps, which can be sufficient for convergence (pLDDT > 0.70 and pTM > 0.70) . For smaller assemblies (e.g. a C3 with protomers composed of 65 amino acids) approximately 500 steps per day could be obtained on a single CPU with 5 GB of memory.

The structures generated from AF2 hallucination were sequence re-designed with ProteinMPNN using only the restrictions that protomer sequences in the oligomeric assembly were tied to be identical, and cysteines were disallowed. For each backbone 24-48 sequences were generated with ProteinMPNN using a temperature of 0.2. The quality of these sequences was assessed with AF2 using all 5 models (model_{1-5}_ptm), checking both the confidence metrics and the structural recapitulation of the original backbone geometry. Sequences were filtered on having AF2 pLDDT > 0.75, and a RMSD to the original protomer backbone < 1.5 Å (computed with TMalign, (*28*)). For each original backbone the four designs with highest AF2 pLDDT were inspected by eye, and up to three MPNN sequences per original input backbone were ordered for experimental testing.

All code is available on GitHub and Zenodo: to be uploaded

ProteinMPNN is available on GitHub: to be uploaded

AF2 is available at: https://github.com/deepmind/alphafold

RoseTTAFold is available at: https://github.com/RosettaCommons/RoseTTAFold

### RoseTTAFold prediction of oligomers

An updated version of RoseTTAFold was used to evaluate designed oligomers. This RoseTTAFold model has multiple architectural improvements over the original published model, including; 1) use of a 3D track from the beginning, with coordinates from a template or the previous recycling round, 2) communication between 1D, 2D, and 3D tracks through attention biasing, and 3) use of recycling that executes the network multiple times with the updated input embeddings based on outputs from the previous cycle. The model was trained with 3 recycling steps. The training dataset comprised; 1) both single-chain and biologically relevant complex structures from the PDB released before April 30, 2020, and 2) AlphaFold2 model structures for UniRef50 representatives. For the examples used during training that were oligomers, we added 200 to the residue numbers of the following subunits to indicate chain breaks to the network. Two rounds of model training were performed; 1) an initial training (200 epochs, with 25600 examples per epoch and a batch size of 64) based on the masked language recovery loss, distogram prediction loss, predicted LDDT loss, and FAPE loss followed by, 2) fine-tuning (50 epochs, with 25600 examples per epoch and a batch size of 64) with additional loss terms on bond geometry and van der Waals scoring function. We trained the model with a crop size of 256 residues, and then fine-tuned it with a larger crop (384 residues). The AdamW Optimizer with default pytorch parameters was used. For the initial training we linearly increased the learning rate to 0.001 over the first 1000 optimization steps, and further decreased the learning rate by a factor of 0.95 for every additional 5000 optimization steps. The fine-tuning stage started from the pre-trained model weights, and used the lower learning rate (0.0005), no warm-up steps, and the same step-wise learning rate decay.

During inference we added 200 to the residue indices of subsequent subunits to indicate chain breaks, as we did during model training. The model was recycled 20 times, and the predicted structure having the highest LDDT estimation was selected. The oligomer structure predictions were generated from the designed sequence only, without any MSA or template information.

Comparison between designs and natural proteins

The outputs generated during AF2 hallucination and ProteinMPNN sequence design were assessed for their sequence and structure novelties. Sequence homologues were searched using BLAST (Protein-Protein BLAST version 2.11.0+) against UniRef100 (snapshot from March 2, 2022) and the E-value of the best hit reported. Both the sequence of the protomer as well as the repeated sequence motif were queried. In the case of small HALs, the protomer and repeated sequence motif were equivalent, but not in the case of large HALs (i.e. HALCX-Y), where protomers are composed of repeated sequence motifs. Structural comparisons to published structures were performed at the protomer level (using TMalign version 20190425, (*28*)) against the PDB (snapshot from April 15, 2022) and over the whole oligomer (using MMalign version 20210816, (*38*)) against all biounits assigned in the PDB (snapshot from April 15, 2022). In both cases results are reported as TM-score.

Computational assessment of designs

Small HALs that were experimentally tested (both hallucinated and ProteinMPNN sequences) were scored with varying metrics in order to investigate differentiating features between the design classes. Structure prediction confidence metrics from AF2, Rosetta scores, and additional biophysical properties were computed, and receiver operator characteristics (ROC) curves computed for each to identify the features giving the strongest signals (Fig. S5). For each designed sequence, only the AF2 model with the highest pTM score was considered for the analysis, and the structure minimized with Rosetta prior to scoring (beta_nov16 weights). Metrics with extensive properties were normalized to the *oligomer* length, i.e. length of the protomer chain ✕ the symmetry (indicated by the suffix _per_res in the score’s name). Detailed definitions of the different score-terms of the Rosetta energy function can be found in Alford et al. (*39*), and descriptions of the other scores are given below:

frac_{X} : fraction of residue {X} in the sequence.

AF2_ptm : AF2 pTM score.

AF2_{pLDDT|pTM}_mean : mean of the respective array from AF2.

AF2_{pLDDT|pTM}_median : median of the respective array from AF2.

AF2_{pLDDT|pTM}_std : standard deviation of the respective array from AF2.

AF2_{pLDDT|pTM}_min : minimum value of the respective array from AF2.

AF2_{pLDDT|pTM}_max : maximum value of the respective array from AF2.

AF2_{pLDDT|pTM}_range : difference between the maximum and minimum of the respective array from AF2.

9mer : worst RMSD from the structural alignment of all 9-amino acid stretches from the design against fragments from the PDB.

nomega_off_per_res : number of omega angles < 174° (normalized).

ss_sc : measures the geometric surface complementarity of protein secondary structure elements, as defined by Lawrence & Colman (*40*).

helix_sc : same but for helices only.

loop_sc : same but for loops only.

packstat : packing statistics (higher is better).

holes : Z-score of the amount of void within the structure, compared to the PDB.

cav_vol_per_res : sum of cavity volumes (reported in Å^3^), normalized.

psipred_match : fraction of residues that match the secondary structure assignment of PSIPRED (version 4, (*41*)).

psipred_mismatch_prob : geometric average of the probability of picking the wrong secondary structure type at all residue positions (from PSIPRED).

psipred_probability : sum of Boltzmann-weighted probabilities (from PSIPRED, T=0.6) of each residue adopting the correct secondary structure, where 0 is complete secondary structure agreement, and 1 is no agreement.

frac_DSSP_extended : fraction of extended secondary structure (assigned by DSSP, (*42*)).

frac_DSSP_helix : fraction of helical secondary structure (assigned by DSSP).

pyrosetta_sap : spatial aggregation propensity, which is defined as the effective dynamically exposed hydrophobicity of a certain patch on the protein surface (*43*).

pyrosetta_sap_per_res : same but normalized.

buried_npsa_per_res : buried non-polar surface area (reported in Å^2^), normalized.

exposed_npsa_per_res : exposed non-polar surface area (reported in Å^2^), normalized.

exposed_hydrophobics_per_res : sum of solvent-accessible surface area (SASA) above 20 Å^2^ for hydrophobic residues (FAMILYVW), normalized.

total_sasa_per_res : total SASA (reported in Å^2^), normalized.

hydrophobic_sasa_per_res : total SASA (reported in Å^2^) of hydrophobic residues, normalized.

polar_sasa_per_res : total SASA (reported in Å^2^) of polar residues, normalized.

total_charge_per_res : total charge of the protein (sum of -D, -E, +K, +R), normalized.

Representation of the structural space

A representation of the structural space covered by the outputs of the hallucination trajectories compared to all *de novo* cyclic structures deposited in the PDB is shown in Fig. 1B. The plot was obtained by Multidimensional scaling (as implemented in the sklearn python library) on a pre-computed pairwise distance matrix. Pairwise distances were defined as 1-TM-score, and the score computed with TMalign (version 20190425). The list of 162 *de novo* cyclic structures was obtained by using the following gate on a snapshot of the PDB from April 17, 2022:

Entry Polymer Composition == homomeric protein &

Polymer Entity Sequence Length >= 40 &

Structure Keywords contains 'de novo' &

Type == Cyclic

1ec5,1g6u,1jm0,1jmb,1lt1,1mft,1ovr,1ovu,1ovv,1u7j,1u7m,1uw1,1vjq,1y47,1y66,2gjf,2gjh,2i7u,2jst,2kik,2mg4,2p05,2p09,2wqh,2zgd,2zgg,3cwo,3dgo,3lt8,3lt9,3lta,3ltb,3ltc,3ltd,3m22,3m24,3mlg,3ol0,3rhu,3tdm,3tdn,3v1b,3v1c,3v1d,3v1e,3v1f,3vjf,3ww7,3ww8,3wwb,3wwf,4db8,4dba,4etj,4f2v,4glu,4hxt,4loa,4lpu,4lpv,4lpw,4lpx,4lpy,4m6a,4ndj,4ndk,4ney,4nez,4o60,4ow4,4pww,4qfv,4rjv,4wpy,4yfo,4yxy,4zcn,4zxz,5a0o,5bvb,5c39,5di5,5dn0,5dns,5dqa,5dra,5dzb,5eil,5f53,5h78,5hpn,5hry,5hrz,5hs0,5i1z,5izs,5j0h,5j0i,5j0j,5j0k,5j0l,5j10,5j2l,5j73,5k7v,5kay,5kba,5kwd,5l0p,5od9,5tph,5u35,5vl4,5ys7,6ff6,6g6q,6idc,6iei,6kos,6m6z,6msq,6msr,6n9h,6naf,6nek,6nla,6nx2,6ny8,6nye,6nyi,6nyk,6nz1,6nz3,6o0c,6o0i,6o35,6qsh,6tjb,6tjc,6tjd,6u1s,6v8e,6veh,6w40,6w6x,6wxo,6wxp,6xh5,6xi6,6xns,6xr2,6xss,6xt4,6y7n,6zv9,7ax0,7bww,7dns,7k3h,7kxs,7m0q,7nbi

### Plasmid construction

Plasmids for expressing HALs were constructed from synthetic DNA according to the following procedure: Linear DNA fragments (Integrated DNA Technologies, IDT eblocks) encoding design sequences and including overhangs suitable for a BsaI restriction digest were cloned into custom target vectors using Golden Gate Assembly. All subcloning reactions resulted in C-terminally HIS-tagged constructs, either as: MSG-design-GSHHHHHH (entry vector LM670) or a MSG-design-GSGSHHWGSTHHHHHH (entry vector LM627), where the underlined sequence is the SNAC-tag used for cleaving the HIS-tag for crystallization (*44*).

The entry vectors for Golden Gate cloning are modified pET29b+ vectors that contain a lethal ccdb gene between the BsaI restriction sites that is both under control of a constitutive promoter and in the T7 reading frame. The lethal gene reduces background by ensuring that plasmids that do not contain an insert (and therefore still carry the lethal gene) kill transformants. The vectors were propagated in ccdb resistant NEB Stable cells (New England biolabs C3040H, always grown from fresh transformants). Plasmids were deposited with Addgene.

Golden Gate reactions (5 uL per well) were set up on a 96 well PCR plate as:

10x T4 Buffer 0.5 uL 10x T4 Buffer (New England Biolabs B0202S)

Vector 10-20 fmol Vector (either LM627 or LM670)

BsaI-HFv2 (3U) 0.15 uL BsaI-HFv2 (New England Biolabs R3733L)

T4 Ligase (100U) 0.25uL T4 Ligase (New England Biolabs M0202L)

- (20-40 fmol) linear DNA fragment, typically 1 uL of 10 ng/uL stock

Complete with nuclease-free water to 5 uL total reaction volume.

The reactions were incubated at 37 °C for 20 minutes, followed by 5 min at 60 °C in a thermocycler (Biorad T100) with the lid heated to 105 °C.

Small-scale protein solubility screen

For initial solubility screens, Golden Gate reaction mixtures were transformed into BL21(DE3) (New England Biolabs) as follows: 1 uL of reaction mixture was added to 6-8 uL of competent cells on ice in a 96 well PCR plate. The mixture was incubated on ice for 30 minutes, then heat-shocked for 10 s at 42 °C in a block heater (IKA Dry Block Heater 3), then rested on ice for 2 minutes. Subsequently, 100 uL of room temperature SOC media (New England Biolabs) was added to the cells, followed by incubation at 37 °C with shaking at 1000 rpm on a Heidolph Titramax1000 / Incubator 1000.

The transformations were then grown in a 96 well deep-well plate (2 mL total well volume) in autoclaved LB media supplemented with 50 μg mL^-1^ Kanamycin at 37 °C and 1000 rpm. In the following protocols all growth plates were covered with breathable film (Breathe Easier, Diversified Biotech) during incubation.

The following day, glycerol stocks were made from the overnight cultures (100 uL of 50 % [v/v] Glycerol in water mixed with 100 uL bacterial culture, frozen and kept at -80 °C. Subsequently, two 96 deep well plates were prepared with 900 uL per well of autoclaved Terrific Broth II (MP biomedicals) supplemented with 50 μg mL^-1^ Kanamycin, and 100 uL of the overnight culture were added and grown for 1.5 h at 37 °C, 1200 rpm (Heidolph Titramax1000 / Incubator 1000). The cultures were then induced with IPTG by adding 10 uL of 100 mM (final concentration approximately 1 mM) per well with an electric repeater pipette (Eppendorf, E4x series), and grown for another 4 h at 37 °C, 1200 rpm. Cultures were combined into a single 96 well plate for a total culture volume of 2 mL and harvested by centrifugation at 4000 x g for 5 min. Growth media was discarded by rapidly inverting the plate, and harvested cell pellets were either processed directly, or frozen at -80 °C.

Proteins were purified by HIS tag-based ​​Immobilized metal affinity chromatography (IMAC). Bacterial pellets were resuspended and lysed in 300 uL B-PER chemical lysis buffer (Thermo Fisher Scientific) supplemented with 0.1 mg mL^-1^ Lysozyme (from a 100 mg mL^-1^ stock in 50 % [v/v] Glycerol, kept at -20 °C, Millipore Sigma), 50 Units of Benzonase per mL (Merck/Millipore Sigma, stored at - 20 °C), and 1 mM PMSF (Roche Diagnostics, from a 100 mM stock kept in Propan-2-ol, stored at room temperature). The plate was sealed with an aluminum foil cover and vortexed for several minutes until the bacterial pellet was completely resuspended (on a Vortex Genie II, Scientific Industries). The lysate was incubated, shaking for 5 minutes, before being spun down at 4000 x g for 15 minutes. In the meantime, 75 uL of Nickel-NTA resin bed volume (Thermo Scientific, resin was regenerated before each run and stored in 20 % [v/v] Ethanol) was added to each well of a 96 well fritted plate (25 μm frit, Agilent 200953-100). To increase wash step speed, the resin was equilibrated on a plate vacuum manifold (Supelco, Sigma) by drawing 3 x 400 uL of Wash buffer (20 mM Tris, 300 mM NaCl, 25 mM Imidazole, pH 8.0) over the resin using the vacuum manifold at its lowest pressure setting.

The supernatant (280 uL) of the lysate was extracted after the spin down and applied to the equilibrated resin and allowed to slowly drip through over ~5 minutes. Subsequently the resin was washed on the vacuum manifold with 3 x 400 uL of wash buffer. Lastly the fritted plate spouts were blotted on paper towels to drain excess wash buffer. Then 250 uL of Elution buffer (20 mM Tris, 300 mM NaCl, 500 mM Imidazole, pH 8.0) was applied to each well and incubated for 5 minutes before eluting the protein by centrifugation at 1500 x g for 5 minutes into a 96 well collection plate. Eluate was stored at 4 °C.

Screening samples for EM and initial SDS-PAGE (Biorad Criterion 26well stain free - anykD) analysis to assess solubility were prepared using this method. Correct protomer masses were verified by Liquid chromatography-mass spectrometry (LC-MS, Agilent) on soluble eluates. To identify the molecular mass of each protein, intact mass spectra was obtained via reverse-phase LC/MS with an Agilent G6230B TOF on an AdvanceBio RP-Desalting column (A: H2O with 0.1% Formic Acid, B: Acetonitrile with 0.1% Formic Acid), and subsequently deconvoluted with Bioconfirm using a total entropy algorithm.

Larger-scale protein expression and purification for biophysical studies

Overnight autoinduction cultures were seeded from the glycerol stocks made for the small scale screen. Growth media was TB-II autoinduction media: TB-II (Terrific Broth II, MP biomedicals - prepared according to manufacturer's specifications: 50 g / L, autoclaved) supplemented with Studier 5052 components from a 50x stock (final concentrations: 5 g / L glycerol, 0.5 g / L dextrose, 2 g / L lactose monohydrate), and 2 mM MgSO_4_.

For the initial screen of 150 AF2 hallucinations, 50 mL cultures were grown in 250 mL baffled flasks (24h, 37 °C, 250 rpm). For the subsequent screen of the MPNN designed sequences, 15 mL cultures were grown in 125 mL baffled flasks (16h, 37 °C, 250 rpm). Cultures were harvested by centrifugation at 4000 x g for 5 minutes, and pellets were stored frozen at -80 °C, or processed directly.

The parameters for the purification of the initial 150 AF2 based hallucinations and the MPNN redesigned sequences are given as ( AF2 | MPNN ) differed slightly because of differences in expression culture volume ( 50 mL | 15 mL )

For protein purification, pellets were resuspended in ( 10 mL | 5 mL ) Wash buffer (20 mM Tris, 300 mM NaCl, 25 mM Imidazole, pH 8.0 at room temperature, supplemented with 0.1 mg mL^-1^ Lysozyme, 0.01 mg mL^-1^, Deoxyribonuclease I (DNAse I, Millipore Sigma), 1 mM PMSF) by vortexing for several minutes until the pellet was fully resuspended. The resuspension was sonicated (Qsonica, Q500 with a: 4 pronged horn | 24 pronged horn) as 10 s ON, 10 s OFF, (45% | 80 %) amplitude for 5 minutes of total ON time, and samples were kept on ice during the whole procedure.

The sonicated lysate was centrifuged at (14000 x g | 4000 x g) for 15-45 minutes to remove the insoluble fraction. Plates with 25 μm bottom frits with ( 24 | 48 ) wells (Agilent 201415-100 | 201003-100 ) were filled with ( 1 mL | 0.5 mL ) of bed Ni-NTA resin (Qiagen or Thermo Fisher), and equilibrated with three rinses of Wash buffer (at least 30 resin bed volumes) on a vacuum manifold as described above.

The fritted plate spouts were closed with parafilm, and the supernatant was added to each well. The plate was sealed and incubated lightly agitated for 30 minutes. The supernatant was drained from the resin, and the resin bed washed three times with ( 10 mL | 5 mL ) of Wash buffer (at least 30 resin bed volumes) on the vacuum manifold. Excess Wash buffer was blotted from the spouts on paper towels, and the resin was pre-eluted with 80% resin bed volume of Elution buffer, followed by protein elution into ( 1.1 mL | 0.8 mL ) of Elution buffer (20 mM Tris, 300 mM NaCl, 500 mM Imidazole, pH 8.0).

Size Exclusion Chromatography (SEC)

IMAC eluates were sterile-filtered through a 96 well filter plate (0.2 μm polyethersulphone (PES) membrane, Agilent 204510-100) by centrifugation at 2000 x g for 5 minutes.

Size exclusion chromatography was performed using an autosampler-equipped Akta pure system (Cytiva) on a Superdex S200 Increase 10/300 GL column at room temperature. The running buffer was 20 mM Na-PO4, 100 mM NaCl, pH 7.4 at room temperature. Selected fractions (shown in Figure S4) were pooled and concentrated using Spin filters (3 kDa molecular weight cutoff, Amicon, Millipore Sigma) and stored at 4 °C before downstream characterizations. Protein identities were confirmed by reverse-phase LC-MS as described above.

SEC retention volume to molecular weight equivalencies were calibrated with protein standards (Cytiva LMW and HMW kits for the S75 and S200 columns, respectively).

Samples for electron Microscopy were purified by SEC using a Superdex 6 10/300 GL increase column (Cytiva) and TBS running buffer (25 mM Tris pH 8.0, 100 mM NaCl). SEC elution fractions corresponding to the design's theoretical elution volumes were concentrated in TBS prior to structural and biochemical analysis.

Size Exclusion Chromatography - Multi Angle Light Scattering (SEC-MALS)

Pooled SEC samples were analyzed by SEC-MALS in 20 mM Na-PO4, 100 mM NaCl, pH 7.4 at concentrations between 0.75 and 7.94 mg/mL on a Superdex 75 10/300 or Superdex 200 10/300 column in line with a Heleos multi-angle static light scattering and an Optilab T-rEX detector (Wyatt Technology Corporation). Data was analyzed using ASTRA (Wyatt Technologies) to calculate the weighted average molar mass (Mw) of the selected species and the number average molar mass (Mn) to determine monodispersity by polydispersity index (PDI) = Mw/Mn.

Circular Dichroism (CD)

Circular Dichroism was performed on a Jasco 1500 CD spectrometer with a 6 sample rotating turret. Samples were placed in 1 mm pathlength cuvettes (Hellma QS Quartz cell) at concentrations of 0.25 mg mL^-1^ in 20 mM Na-PO4, 100 mM NaCl, pH 7.4 buffer. The temperature was ramped from 25 °C to 95 °C, recording full CD spectra between 200 and 260 nm in 10 °C intervals, and reading at 222 nm in 2 °C intervals. After reaching 95 °C the samples were allowed to cool back to 25 °C before recording a final spectrum. Samples were recovered, filtered over a 0.2 μm PES membrane, and re-run over SEC as described above.

Crystallography sample preparation and data collection

19 designs were chosen to undergo crystallization screens. Each design was expressed as described above in 0.5 L cultures. Following affinity purification, each design underwent SEC into SNAC cleavage buffer (100 mM CHES, 100 mM NaCl, 100 mM acetone oxime, 500 mM guanidine HCl, pH 8.6). Following SEC, 2 mM of NiCl_2_ was added and the solution was incubated overnight at 37°C. Following cleavage, the solutions containing the cleaved protein products were incubated with 1 mL Ni-NTA resin to bind any uncleaved product, and the flow through was collected. Following SEC into Crystallization buffer (20mM Tris, 50 mM NaCl, pH 8.0), each sample was concentrated to approximately 15 mg mL^-1^. The following sitting drop broad screens were set up at room temperature with three protein:crystallization condition ratios (1:1, 1:2, 2:1) using the mosquito pipetting instrument (sptlabtech): Midas (Molecular Dimensions), Proplex (Molecular Dimensions), JCSG+ (Molecular Dimensions), Morpheus (Molecular Dimensions), Pact Premier (Molecular Dimensions), LMB (Molecular Dimensions), Index (Hampton Research) and PGA (Molecular Dimensions). Each was monitored weekly for crystal growth using the JANSi UVEX imaging system.

The following conditions yielded diffracting crystals for our designs: 0.05 M Cesium chloride, 0.1 M MES pH 6.5, 30% Jeffamine M-600 (HALC3_104); Morpheus condition H5 (HALC3_109); 0.1 M BIS-TRIS pH 6.5, 2.0 M Ammonium sulfate (HALC2_062); 0.2 M Lithium sulfate monohydrate, 0.1 M BIS-TRIS pH 6.5, 25% w/v Polyethylene glycol 3,350 (HALC4_135); 0.1M SPG buffer pH 5 25% w/v PEG 1500 (HALC4_136), 0.04 M Potassium phosphate, 16% PEG 8000, 20% Glycerol (HALC2_068); and 0.2 M Ammonium nitrate pH 6.3, 20% PEG 3350 (HALC2_065). Where required, crystals were cryoprotected with 20% glycerol or 25% ethylene glycol prior to flash freezing in liquid nitrogen. Data collection was done using the Advanced Photon Source synchrotron. Images were integrated using XDS 20220110 (*45*). Aimless (*46*) was used for scaling and merging. Phaser 2.8 (*47*) was used for molecular replacement using the design models as search models (either monomer or oligomeric complex). Models were built using Coot 0.9.8 (*48*) and refined with Phenix refine from Phenix 1.20 (*49*) and RefMac (*50*) from CCP4 7.1 (*46*) suite. All structures were validated using MolProbity 4.5.1(*51*). Crystallographic statistics are available in Table S1.

Negative Stain Electron Microscopy (nsEM):

SEC fractions corresponding to the designs were concentrated in TBS prior to negative stain EM screening. Samples were then immediately diluted 5 to 150 times in TBS buffer (25 mM Tris, 100 mM NaCl, pH 8.0) depending on the concentration of the samples. A final volume of 5 μL was applied on negatively glow discharged, carbon-coated 400-mesh copper grids (01844-F, TedPella Inc.), then washed with Milli-Q Water and stained using 0.75% uranyl formate as previously described (*52*). Air-dried grids were then imaged on either a FEI Talos L120C TEM (FEI Thermo Scientific) equipped with a 4K × 4K Gatan OneView camera at a magnification of 57,000x and pixel size of 2.51 Å. Micrographs collection was automated using EPU software (FEI Thermo Scientific) and were imported into CisTEM software (*53*) or cryoSPARC software (*54*, *55*). CTF estimation was done with CTFFIND4 and a circular blob picker was used to select particles which were then subjected to 2D classification. *Ab initio* reconstruction and homogeneous refinement in Cn symmetry were used to generate 3D electron density maps. All EM maps can be found in supplementary data.

CryoEM Sample Preparation and Data Collection:

CryoEM grids were prepared by diluting protein samples with TBS 1 to 10 times immediately before applying 3.5 μL to glow-discharged 400 mesh, C-flat, 2 micron holes, 2 micron spacing, CF-2/2-4C (CF-224C-100) (Electron Microscopy Sciences) cryoEM grids. For some samples, multiple blots were applied in order to obtain the best particle density. All grids were blotted using a blot force of 0 and 5.5 second blot time at 100% humidity and 4°C and plunge-frozen in liquid ethane using a Vitrobot Mark IV (FEI Thermo Scientific). All cryoEM grids were screened on a Glacios transmission electron microscope (FEI Thermo Scientific) operated at 200 kV and equipped with a Gatan K2 or K3 Summit direct detector. Automated glacios data collection was carried out using Leginon (*56*) at a nominal magnification of 36,000x (1.16 Å/pixel). Movies were acquired in counting mode fractionated in 50 frames of 200 ms at 8.5 e-/pixel/sec for a total dose of ~65e-/Å^2^. Details of data processing for each design are illustrated in Fig. S13-15.

CryoEM data processing:

Multiple datasets were collected for each design and combined early on during processing. See Fig. S13-15 and processing flowcharts for details. Briefly, images were manually curated to remove poor quality acquisitions such as bad ice or large regions of carbon. Dose-weighting and image alignment of all 50 frames was carried out using MotionCor2 (*57*) with 5X5 patch or with cryosparc v2 patch alignment tool with default parameters. Super-resolution data was binned 2X during alignment. Initial CTF parameters were estimated using CTFfind4 (*58*). Particle picking was done with a gaussian blob picker and in some cases followed by a template picker. Particles were extensively classified in 2D to remove ice and noisy particles, yielding in some cases relatively few particles. Starting models for all designs were always obtained *ab initio*, despite clear evidence of the expected design in 2D. FSC curves were generated using cryoSPARC. All EM maps have been deposited in the EMDB (accession codes: EMD-27658, EMD-27659, EMD-27660), and can be found in the supplementary data.

Visualization and figures

All structural images for figures were generated with either PyMOL, Chimera or ChimeraX. Data was processed and figures were plotted using Pandas, MatplotLib, and Seaborn python libraries. Figures were further rendered and assembled using Adobe Illustrator and Inkscape.


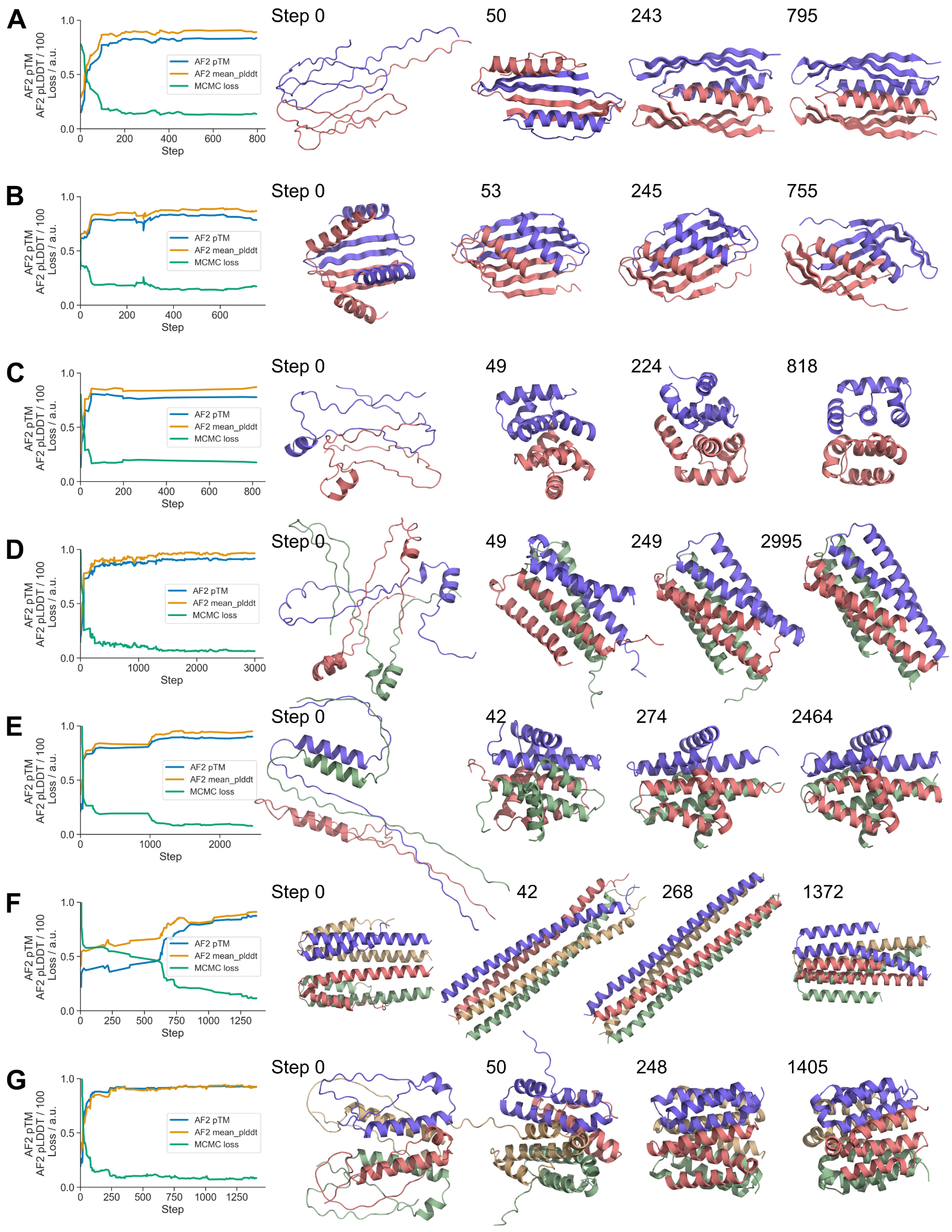


**Fig. S1. MCMC trajectories of oligomer hallucination with intermediate structures shown.** HAL hallucination trajectories with AF2 confidence metrics and the MCMC loss per accepted step shown on the left. Structures of the first step (random sequence initialization), accepted structures near step 50 and 250, and the final accepted step are shown for: (**A**) HALC2_062 (**B**) HALC2_065 (**C**) HALC2_068 (RMSD: 3.91 Å | 0.92 Å). (**D**) HALC3_104 (**E**) HALC3_109 (**F**) HALC4_135 (**G**) HALC4_136. Note that in some cases, even the initial (randomly initialized) sequence is predicted to contain residual structure, albeit with low confidence.


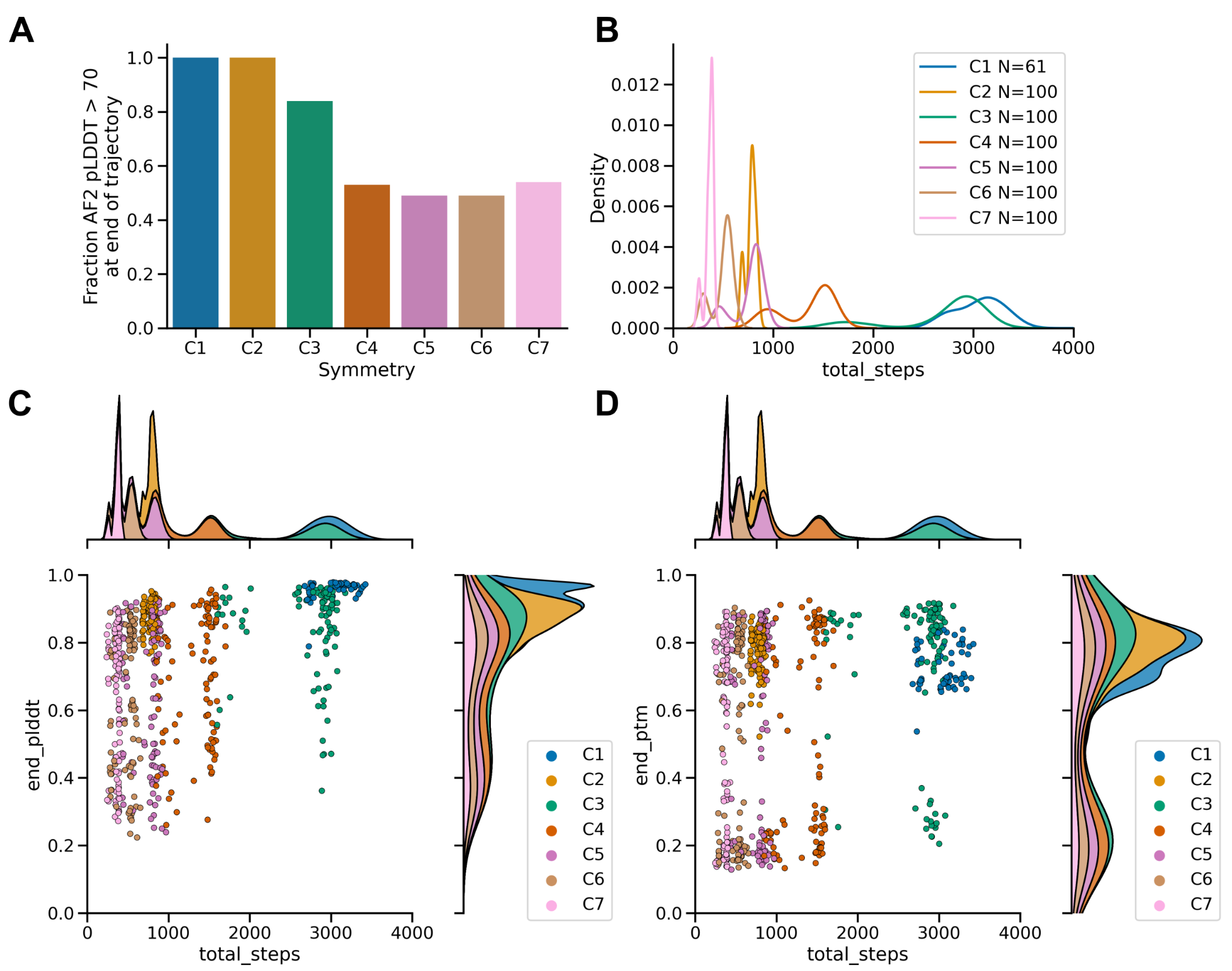


**Fig. S2. Convergence of hallucination trajectories by symmetry class.** (**A**) Higher symmetry leads to fewer designs having high (> 70) AF2 pLDDT scores after the allocated runtime. (**B**) This effect is confounded by the fewer total number of MCMC steps performed for higher symmetries due to the increased computational cost per step. These parameters are displayed in the bottom panels, which show the pLDDT (**C**) or pTM (**D**) score of the last accepted step as a function of the total number of steps. Marginal distributions are shown as stacked KDEs.


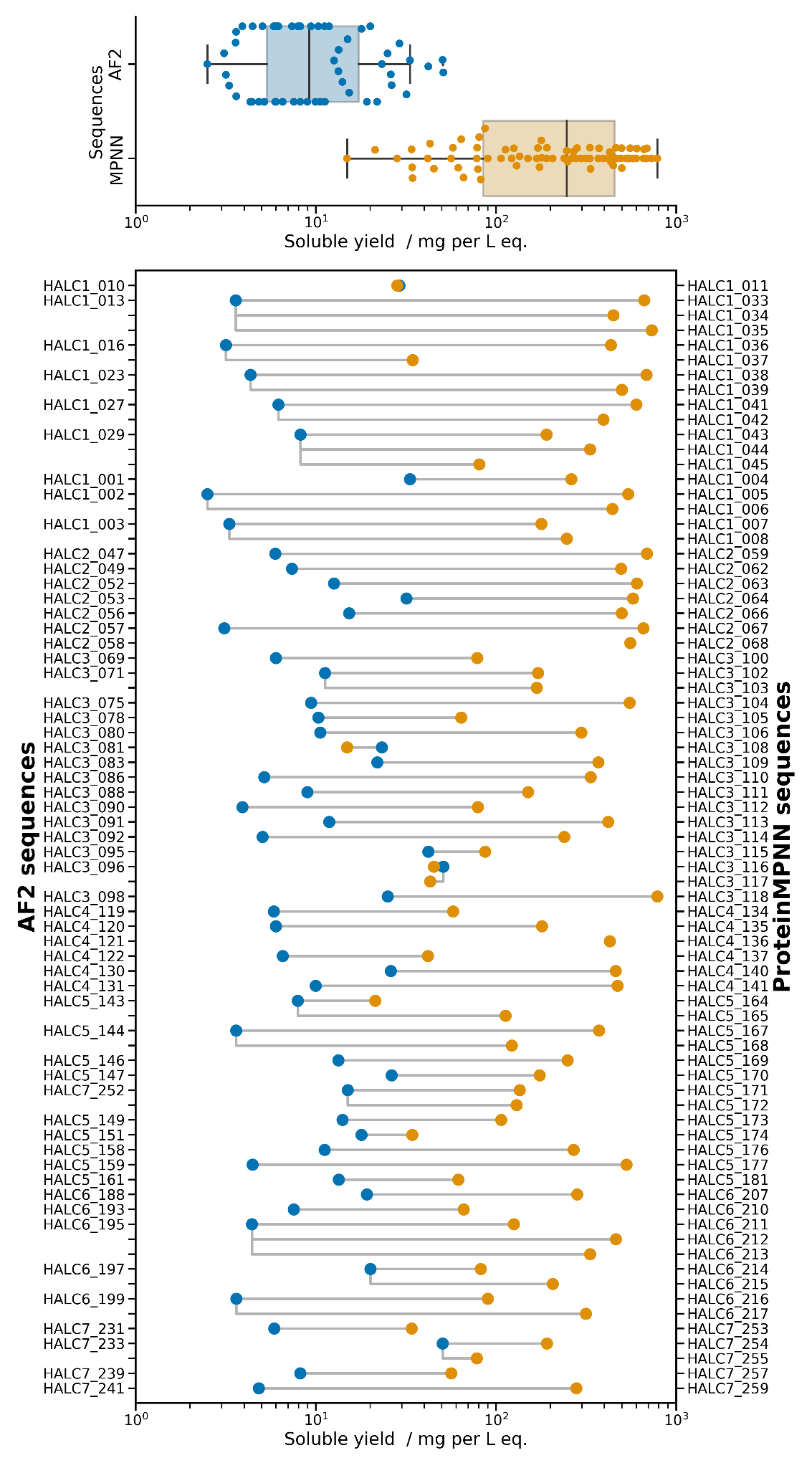

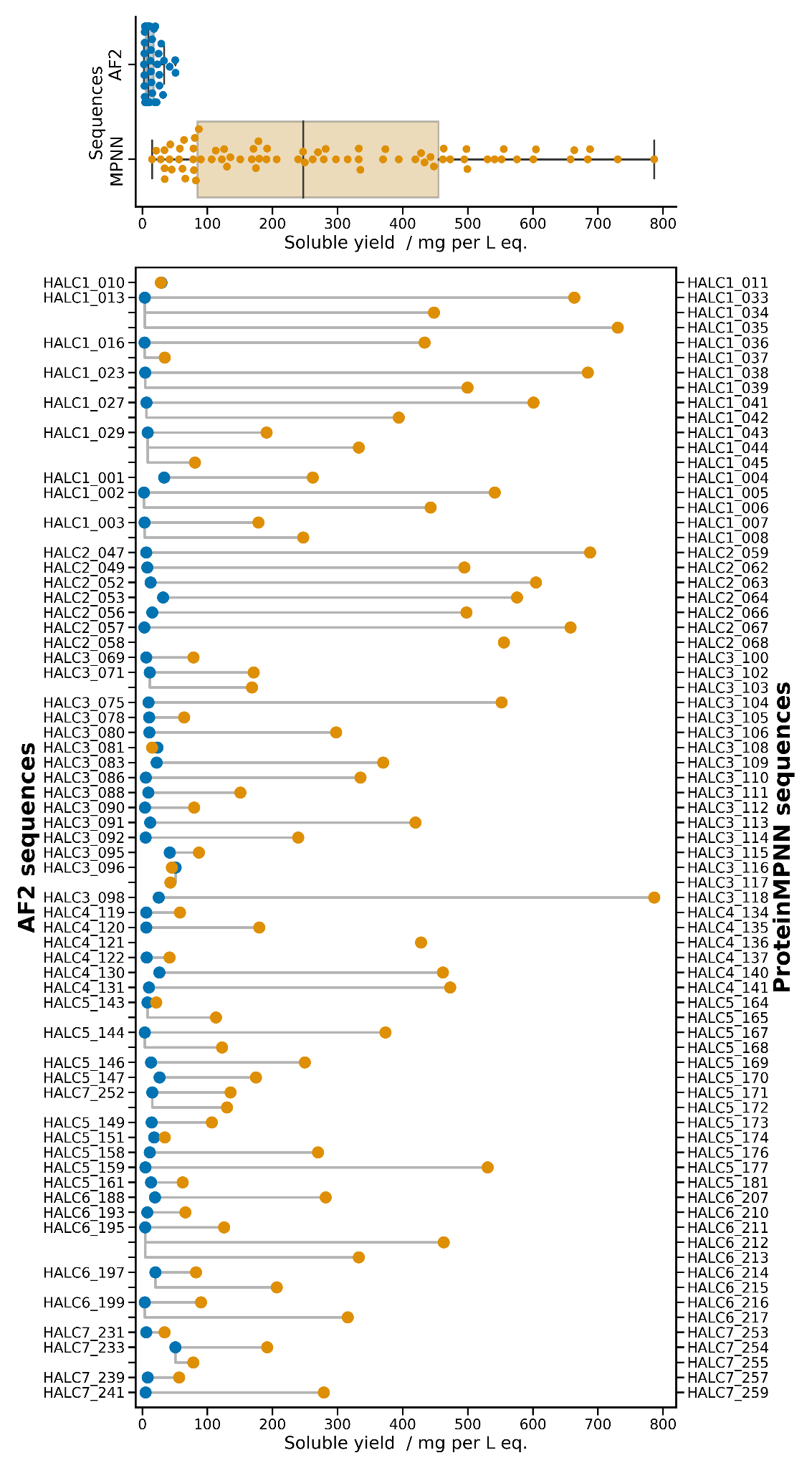


**Fig. S3. Soluble yield of AF2-hallucinated and ProteinMPNN-designed sequences for small HALs.** The plots show the total soluble yield per liter of culture equivalent calculated from integrating the SEC elution profiles (and normalizing by the sequence-specific extinction coefficients) for the original AF2 designs, compared to their ProteinMPNN redesigns. In some cases, more than one ProteinMPNN sequence per backbone was tested. The top two plots summarize the difference in yield: for the AF2 designs a median yield of 9 mg per L eq. as compared to 247 mg per L eq. for the MPNN sequences. The top plot shows the data on a log scale.


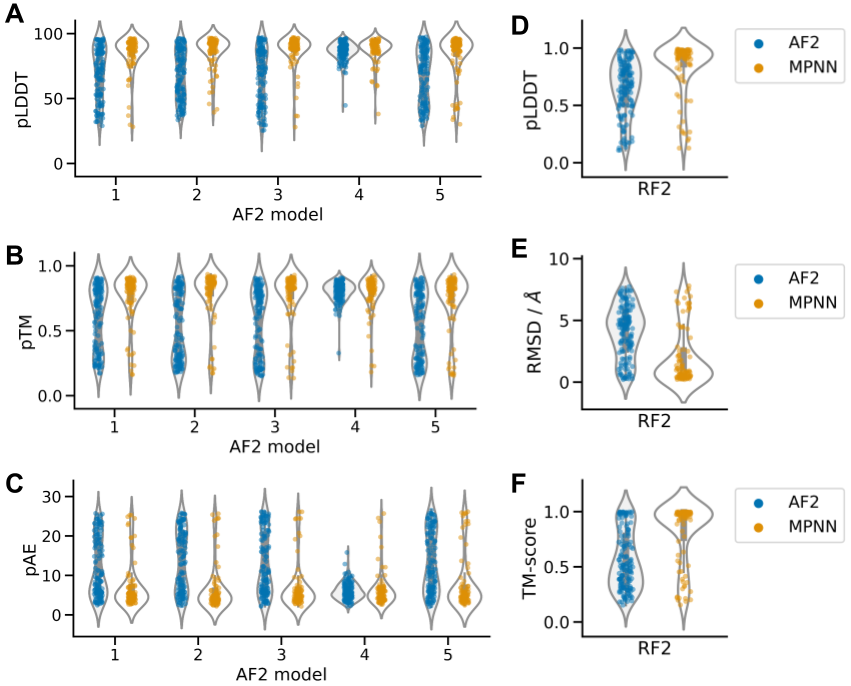


**Fig. S4. AF2-hallucinated sequences are not well cross-validated, unlike ProteinMPNN ones. A**-**C** AF2 predictions of AF2-hallucinated sequences indicate high prediction confidence for the model used during design (model_4_ptm), but low confidence for all other models. In contrast, ProteinMPNN-designed sequences predict better across all five models. **D**-**F** RF2 predictions compared to the designed structure. (**D**) RF2 fails to predict AF2 designed sequences reliably, but confidently predicts ProteinMPNN sequences. **E**-**F** RF2 models of ProteinMPNN sequences are close to their designs, while models predicted from the sequence of AF2 hallucinations fail to recapitulate the designed structure.

**
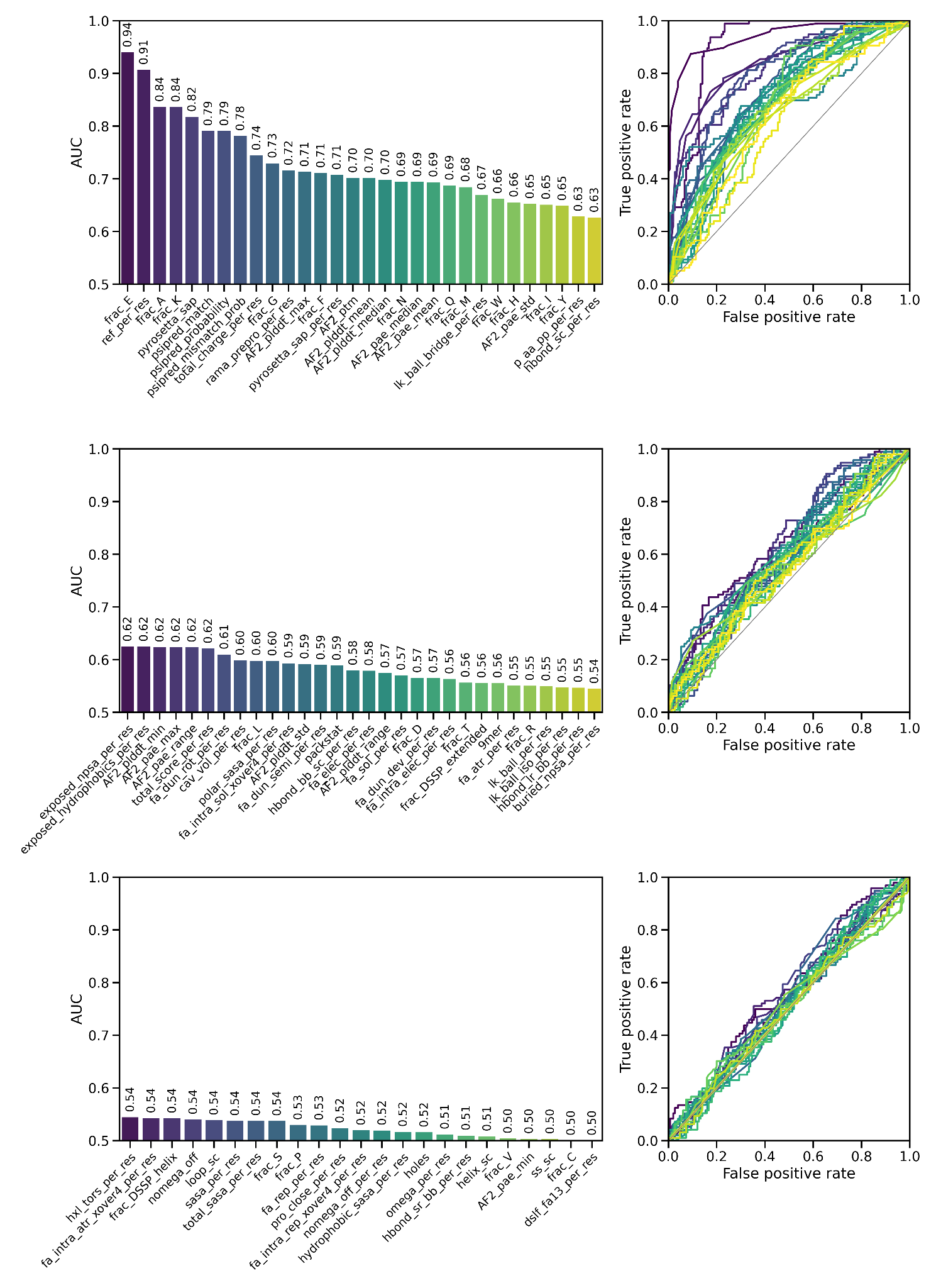
**

**Fig. S5. Features distinguishing AF2-hallucinated designs and ProteinMPNN designs.** Receiver operating characteristic (ROC) curves (right) and area under the curve (AUC) values (left) for 84 features used as single predictors of design class (AF2 hallucination vs. ProteinMPNN). One-dimensional features such as fractional content of charged amino acids, two-dimensional features (match between predicted secondary structure and design), and exposed surface hydrophobics are the strongest differentiating features between the two design classes. Detailed descriptions of the meaning of each score can be found in Supplementary Materials and Methods.


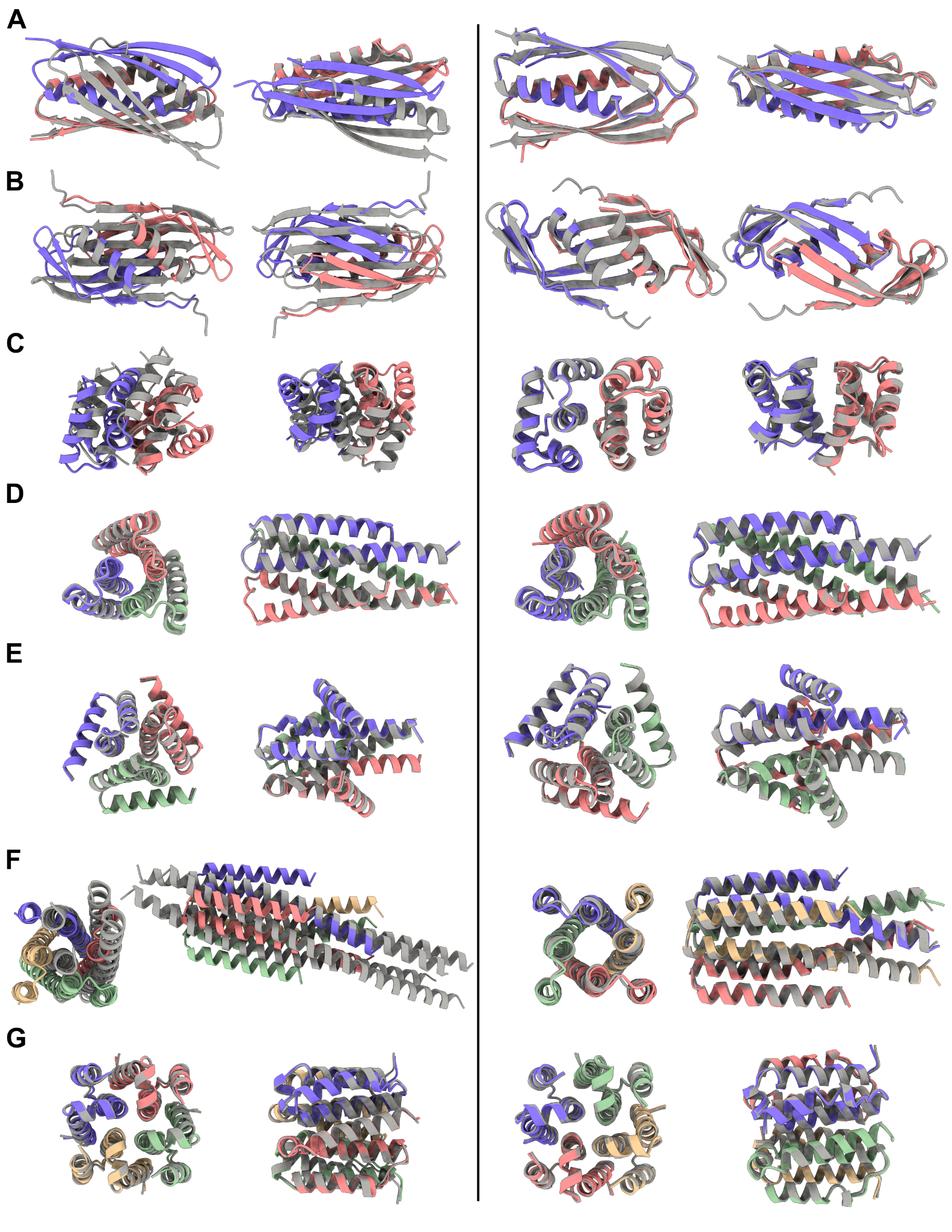


**Fig. S6. RoseTTAFold2 accurately predicts the structures of crystallized HALs.** RoseTTAFold2 predictions of AF2-hallucinated sequence compared to the original AF2 hallucinated structure (left). RoseTTAFold2 predictions of ProteinMPNN-designed sequences for the same backbones compared to their crystal structures (right). (**A**) HALC2_062 (RMSD: 2.75 Å | 0.83 Å ). (**B**) HALC2_065 (RMSD: 4.28 Å | 1.11 Å). (**C**) HALC2_068 (RMSD: 3.91 Å | 0.92 Å). (**D**) HALC3_104 (RMSD: 0.27 Å | 0.42 Å). (**E**) HALC3_109 (RMSD: 0.48 Å | 0.55 Å). (**F**) HALC4_135 (RMSD: 4.08 Å | 0.72 Å). (**G**) HALC4_136 (RMSD: 0.91 Å | 0.37 Å). The AF2/crystal structures are colored by chain, and the RoseTTAFold2 predictions are shown in gray.

**
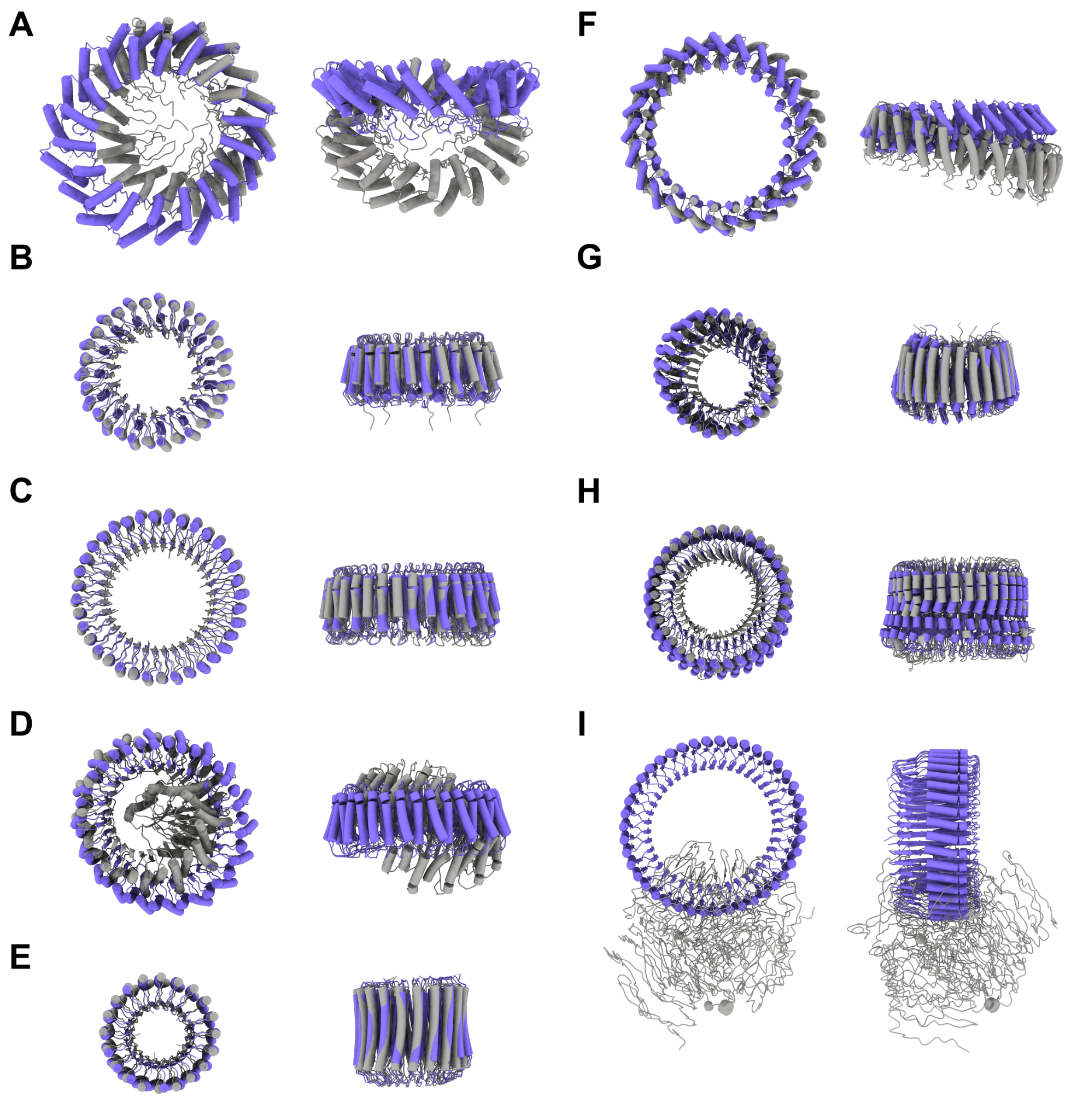
**

**Fig. S7. RoseTTAFold2 predictions aligned to design models of EM-verified HALs.** Design ID (RMSD of AF2 model against RF2 prediction) (**A**) HALC6_220 (3.87 Å). (**B**) HALC15-5_262 (1.33 Å). (**C**) HALC18-6_265 (0.73 Å). (**D**) HALC18-6_278 (3.35 Å). (**E**) HALC20-5_308 (1.18 Å). (**F**) HALC24-6_316 (3.66 Å). (**G**) HALC25-5_341 (3.09 Å). (**H**) HALC33-3_343 (3.67 Å). (**I**) HALC42-7_351 (7.99 Å). RF2 predictions in gray, AF2 design models in purple.


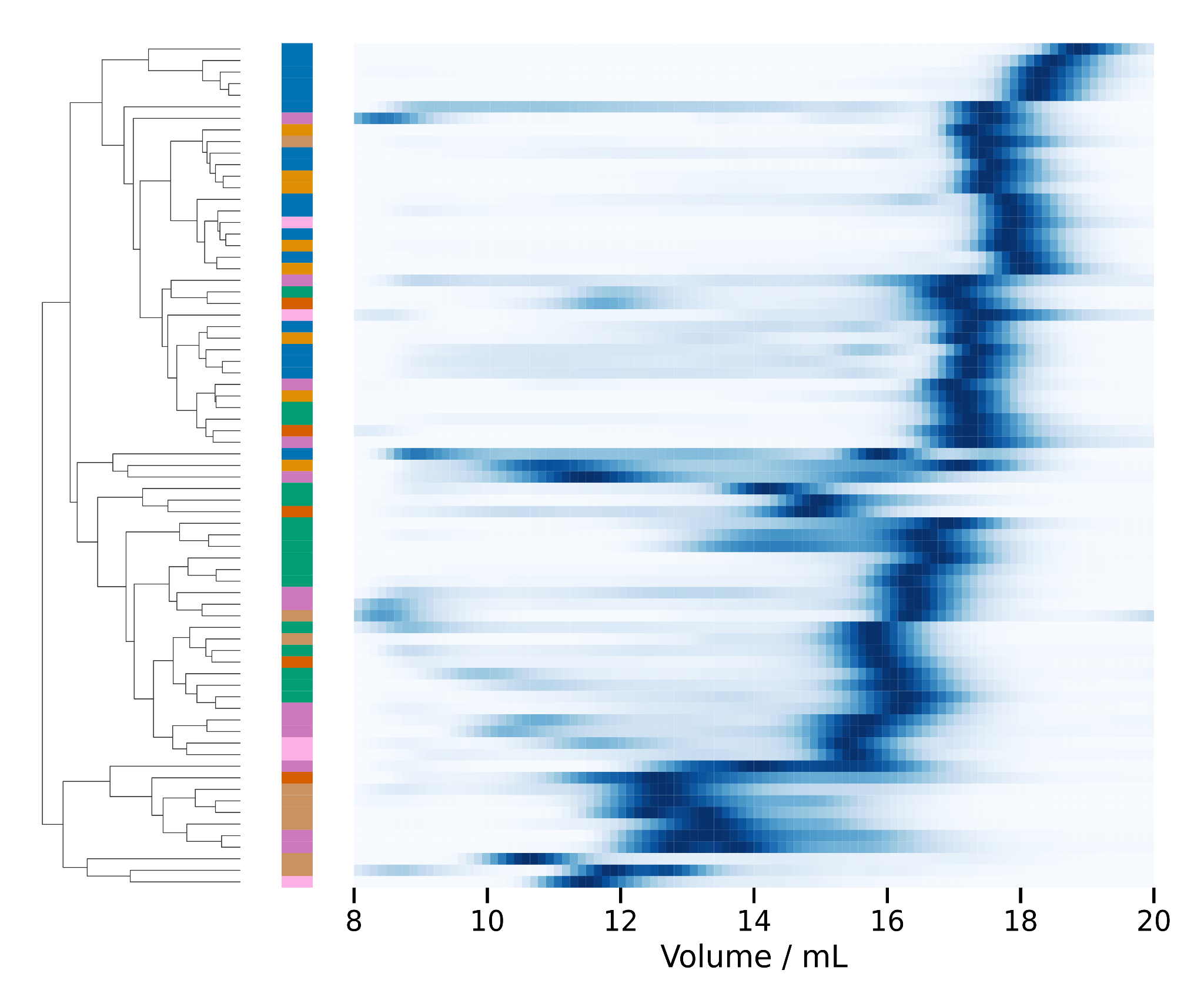


**Fig. S8. Normalized SEC elution profiles of small HALs.** Samples were run on a Superdex 200 increase 10/300 GL following IMAC purification. The results are shown clustered by profile similarities (using Euclidean distances), and for each, the designed oligomeric state is indicated by color (C1; blue, C2; orange, C3; green, C4; red, C5; dark pink, C6; brown, C7; light pink).


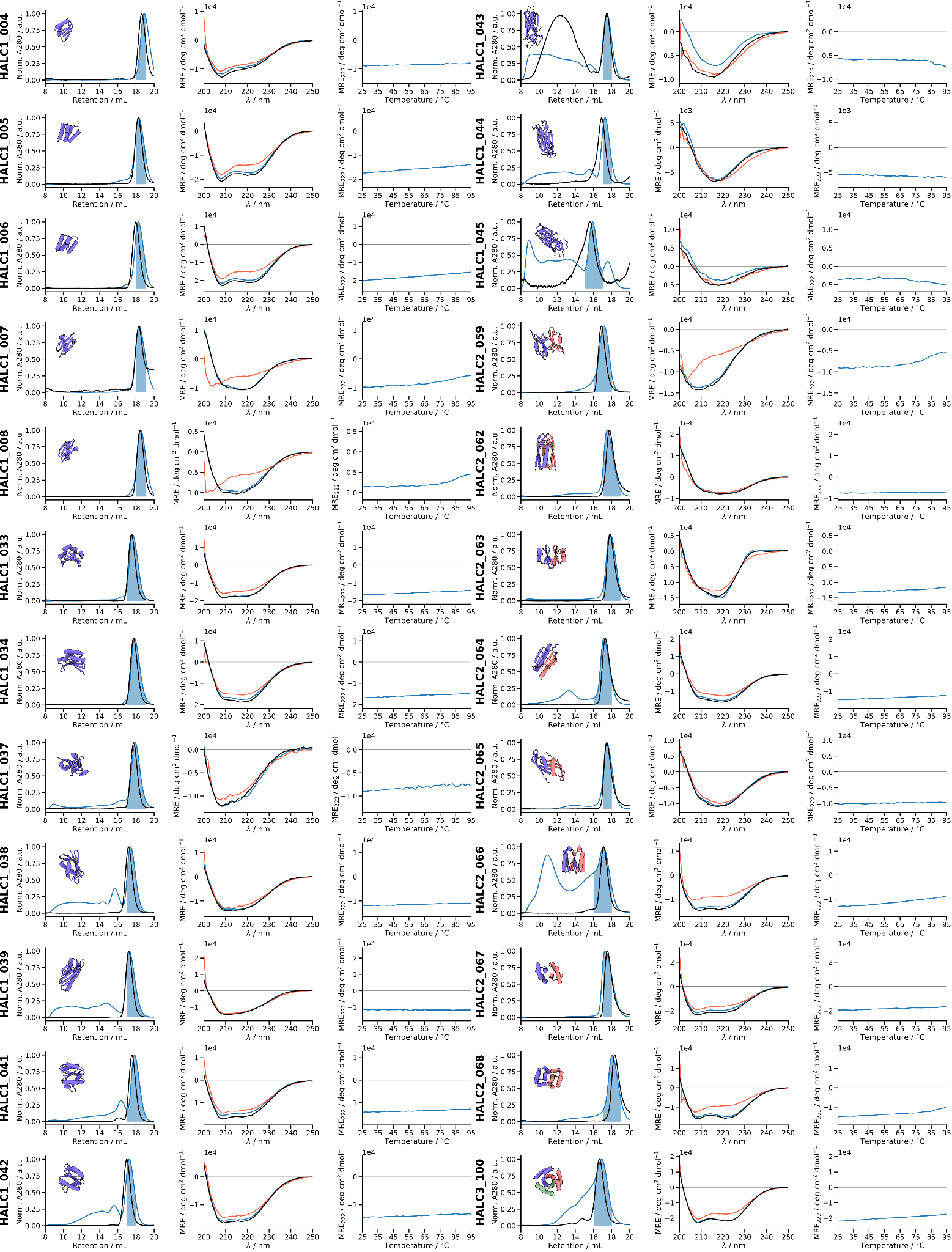


**Fig. S9. Characterization of HALC1_004 to HALC3_100.** The first column shows the SEC elution profile (Superdex 200 increase 10/300 GL) after IMAC (blue, collected fractions indicated by the shaded region), and after heating the sample to 95 °C (black). The second column shows the CD spectra at 25 °C (blue), at 95 °C (red) and after cooling back to 25 °C (black). The third column shows the circular dichroic signal at 222 nm during temperature ramping.


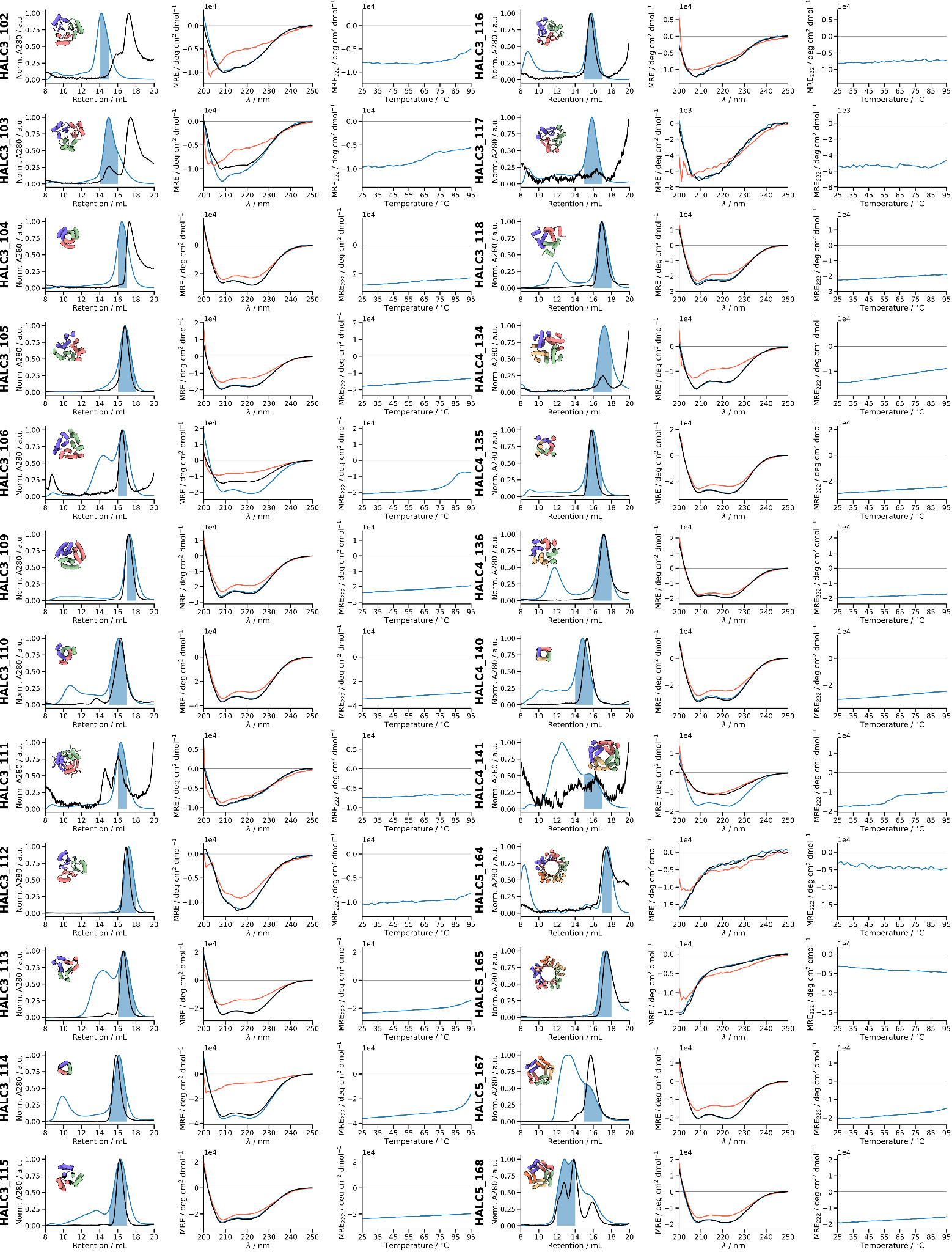


**Fig. S9 (continued). Characterization of HALC3_102 to HALC5_168.** The first column shows the SEC elution profile (Superdex 200 increase 10/300 GL) after IMAC (blue, collected fractions indicated by the shaded region), and after heating the sample to 95 °C (black). The second column shows the CD spectra at 25 °C (blue), at 95 °C (red) and after cooling back to 25 °C (black). The third column shows the circular dichroic signal at 222 nm during temperature ramping.


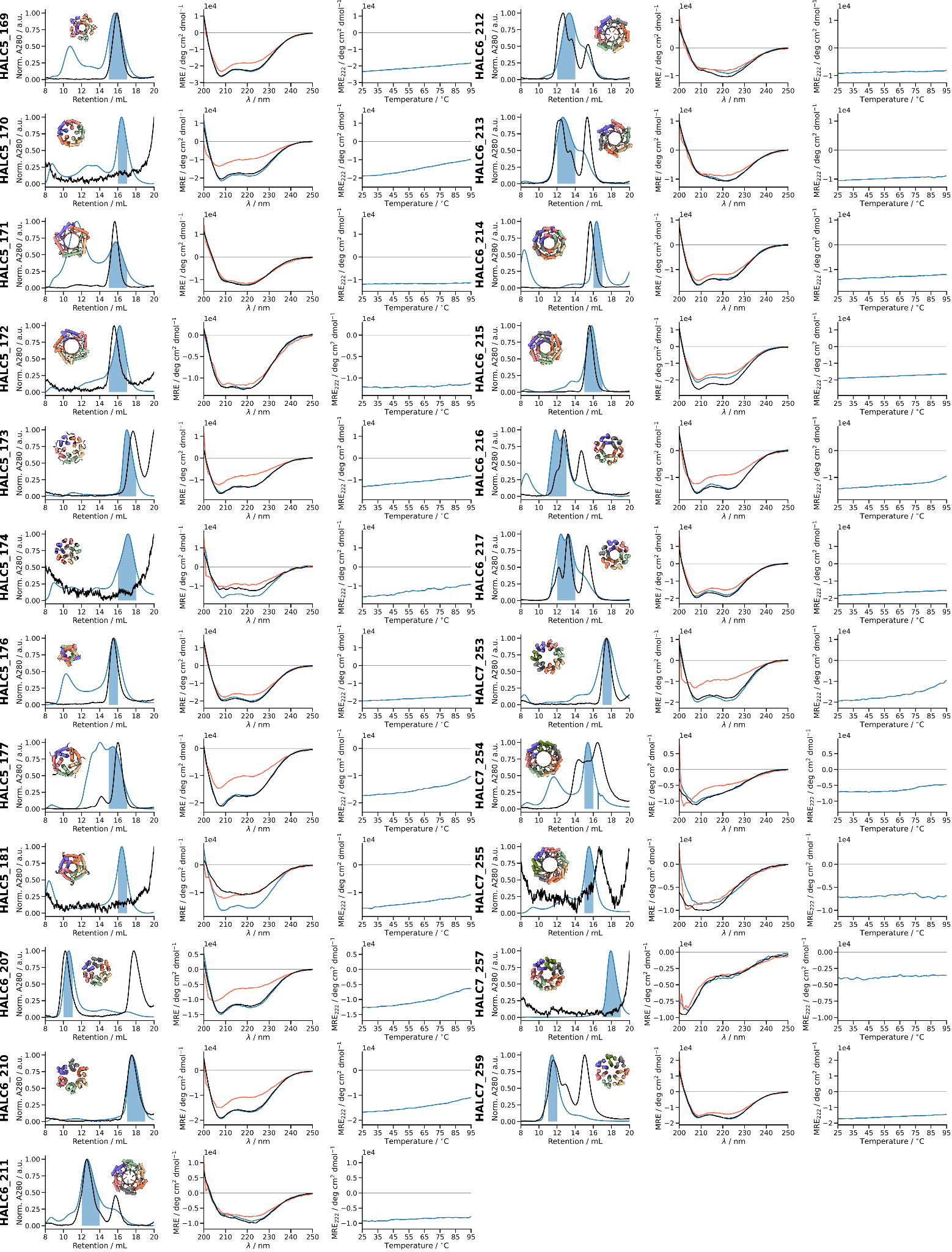


**Fig. S9 (continued). Characterization of HALC5_169 to HALC7_259.** The first column shows the SEC elution profile (Superdex 200 increase 10/300 GL) after IMAC (blue, collected fractions indicated by the shaded region), and after heating the sample to 95 °C (black). The second column shows the CD spectra at 25 °C (blue), at 95 °C (red) and after cooling back to 25 °C (black). The third column shows the circular dichroic signal at 222 nm during temperature ramping.


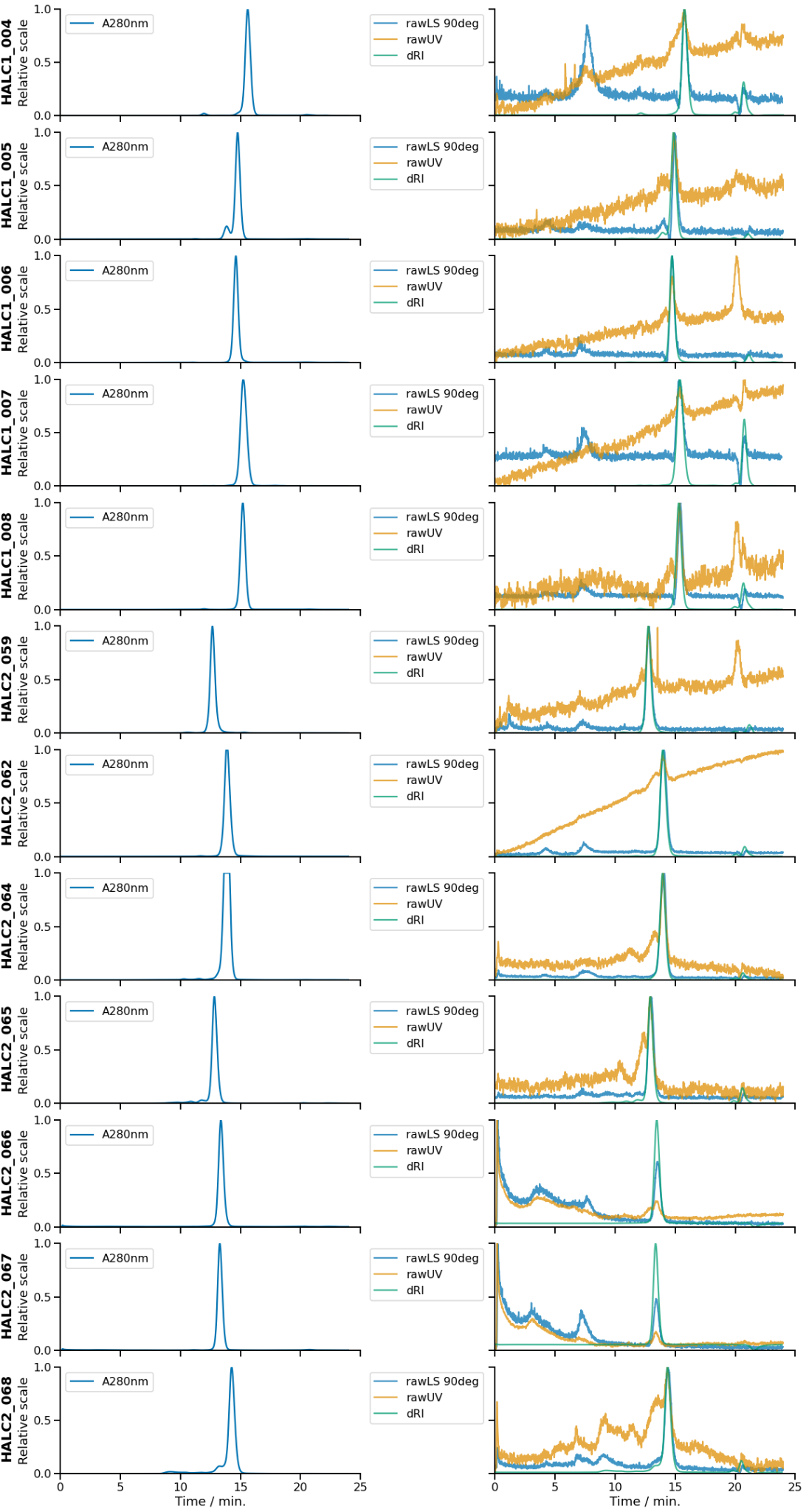


**Fig. S10. Raw SEC-MALS data.** For 24 HALs with sequences designed by ProteinMPNN, absorbance at 215 nm, raw light scattering (LS) signal at a 90 degree angle detector, the raw UV signal, and differential refractive index data (rRI) are shown. The data was collected on a Superdex 75 10/300 column.

**
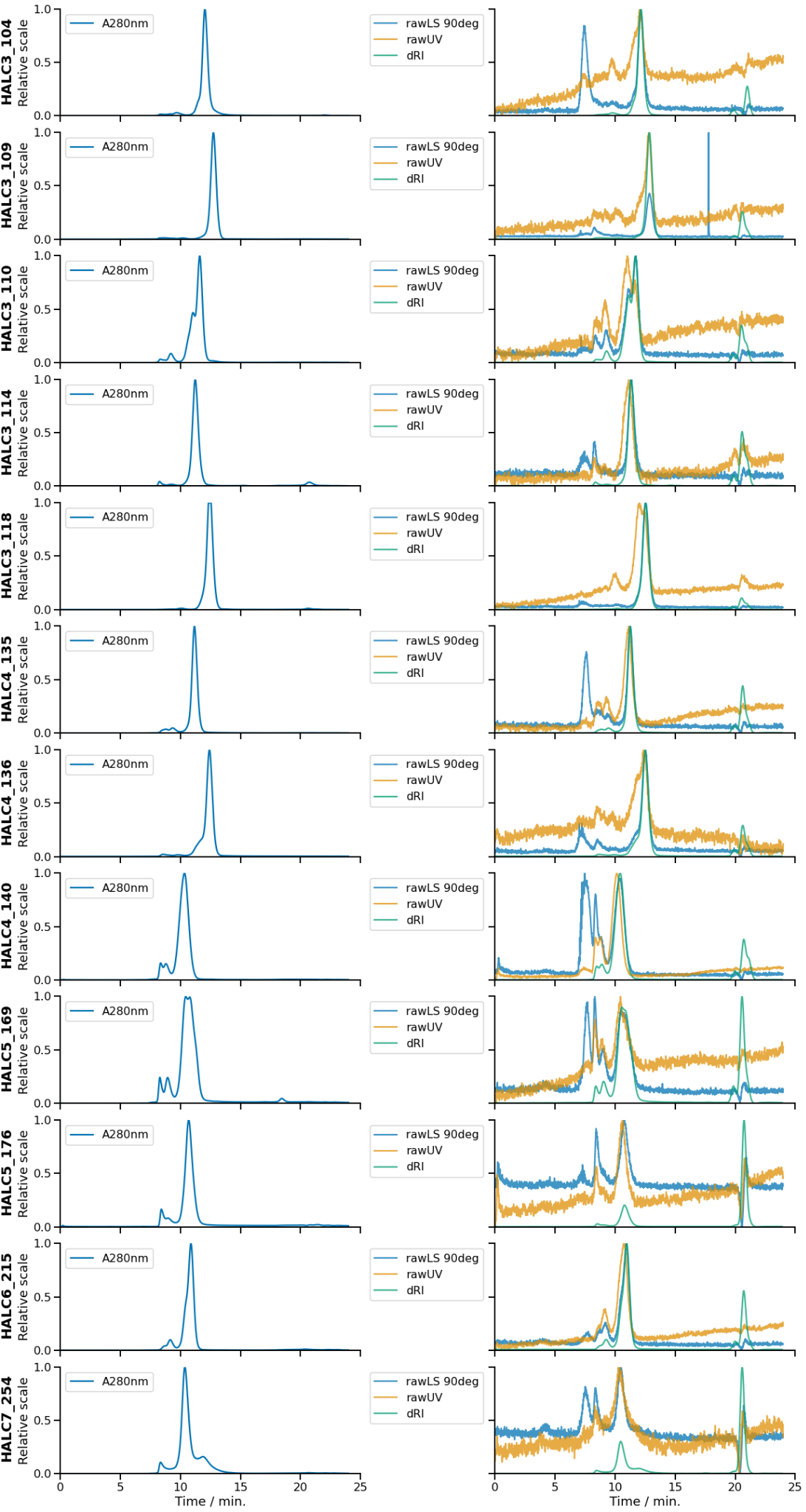
**

**Fig. S10 (continued). Raw SEC-MALS data.** For 24 HALs with sequences designed by ProteinMPNN, absorbance at 215 nm, raw light scattering (LS) signal at a 90 degree angle detector, the raw UV signal, and differential refractive index data (rRI) are shown. The data was collected on a Superdex 75 10/300 column.


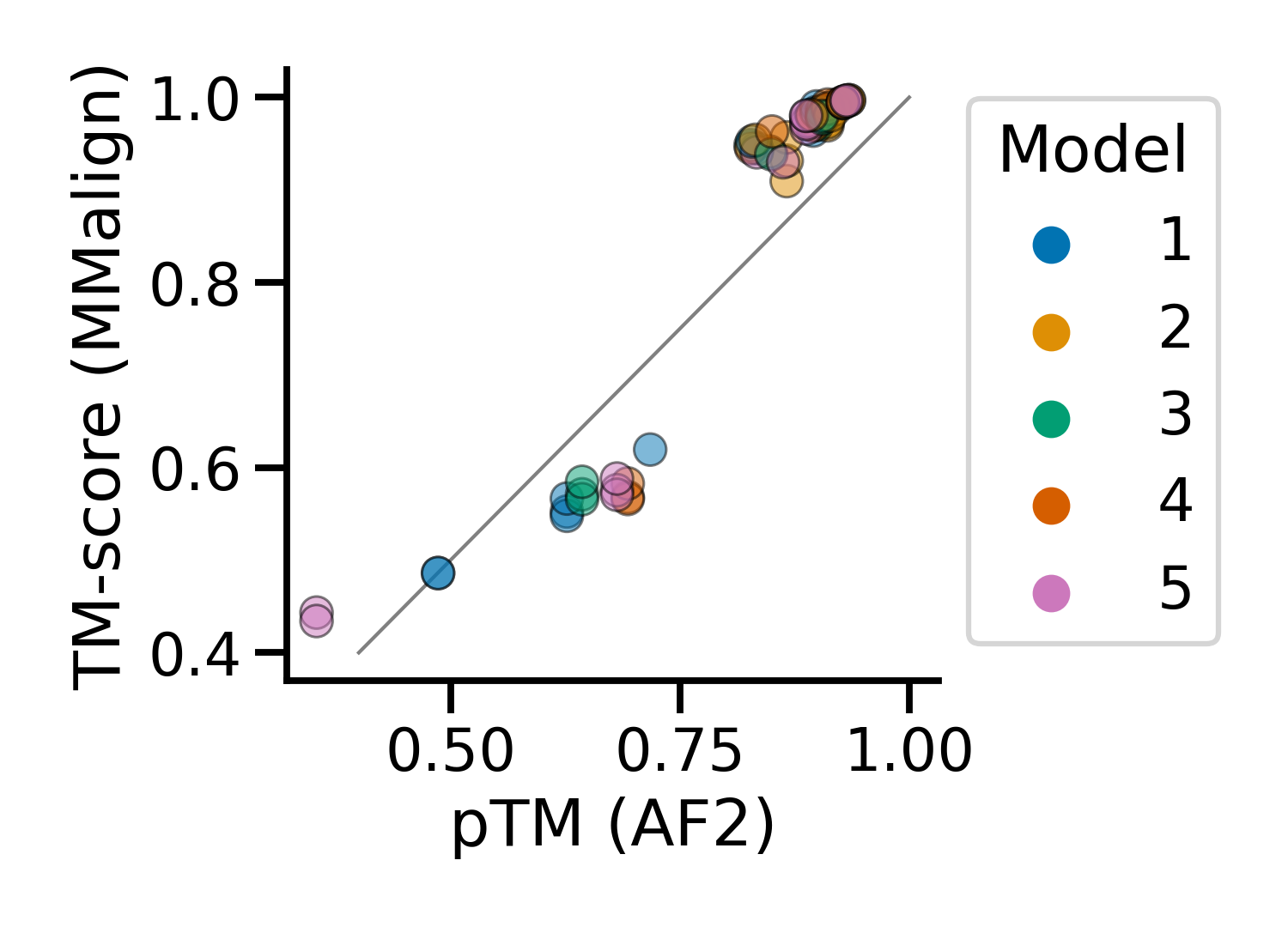

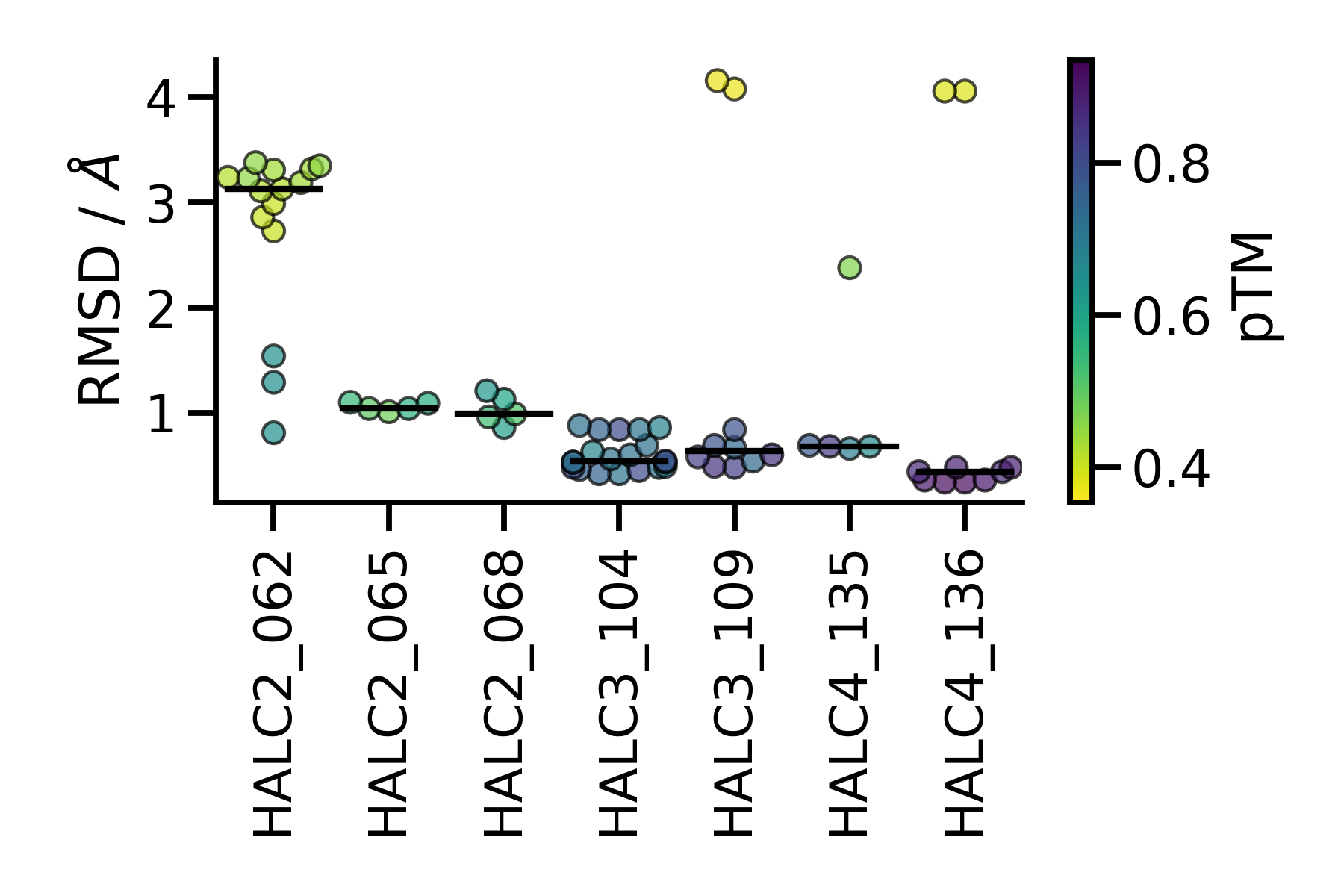


**A**

**B**

**C**


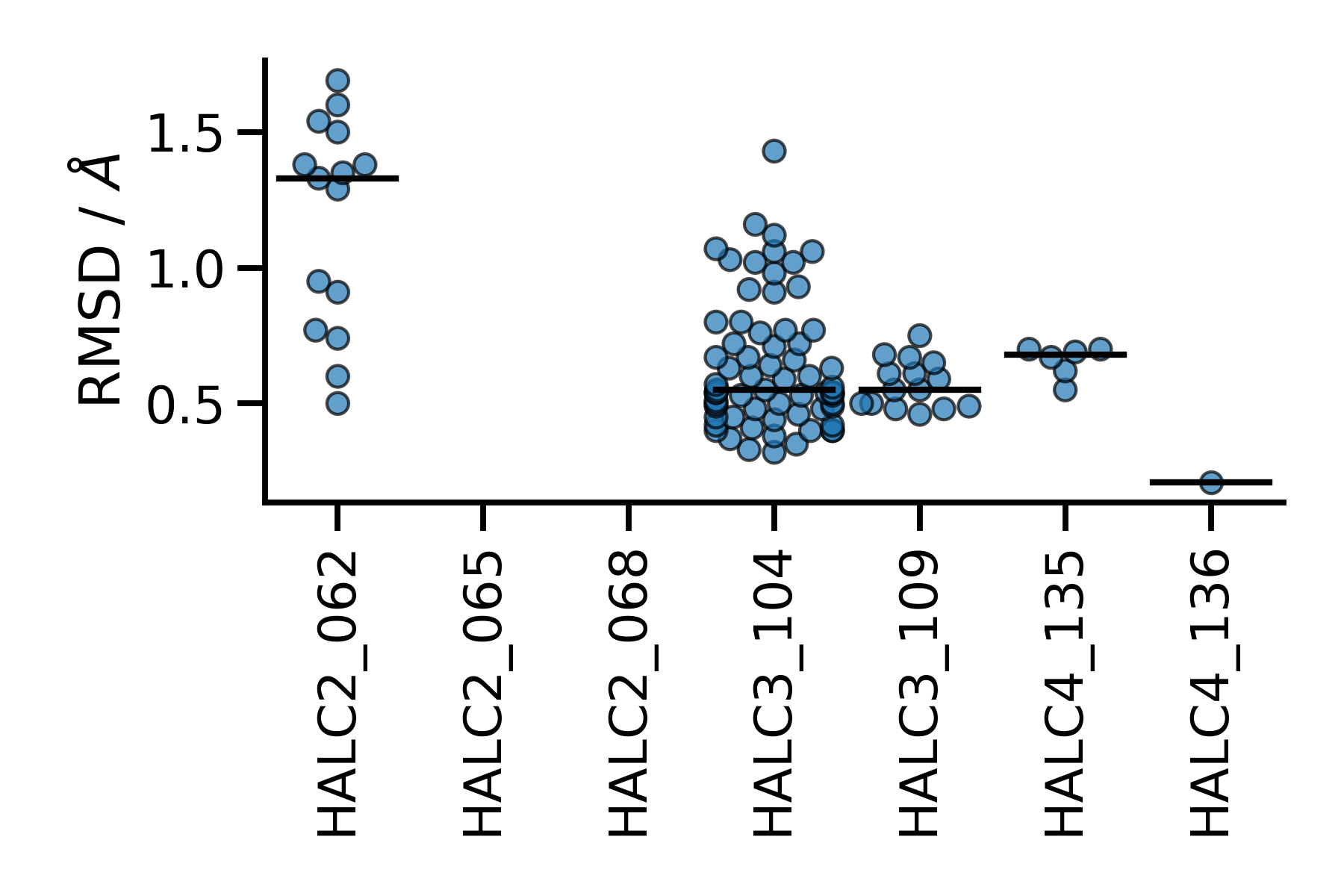


**Fig. S11. Comparison between AF2 models and crystallographic structures.** (**A**) For each design, five models (one for each _ptm model, 10 recycles) were compared to the crystallographic biounit. If multiple biounits were present, alignments against all bionunits are shown. Alignments were generated using MMalign, and the median RMSD for each design is indicated by a horizontal line. Models that were more confidently predicted (higher pTM values) were closer to the experimentally-validated structures as shown by the color bar. (**B**) The pTM value from each AF2 model correlates with the actual TM-score (from MMalign) between design and structures. The parity is indicated by a gray line. (**C**) Structural matching between chains of the asymmetric unit of each design. Pairwise alignments and RMSD values were computed with TMalign, and the median is indicated by a horizontal line. Designs lacking data points only contained one chain in the asymmetric unit.


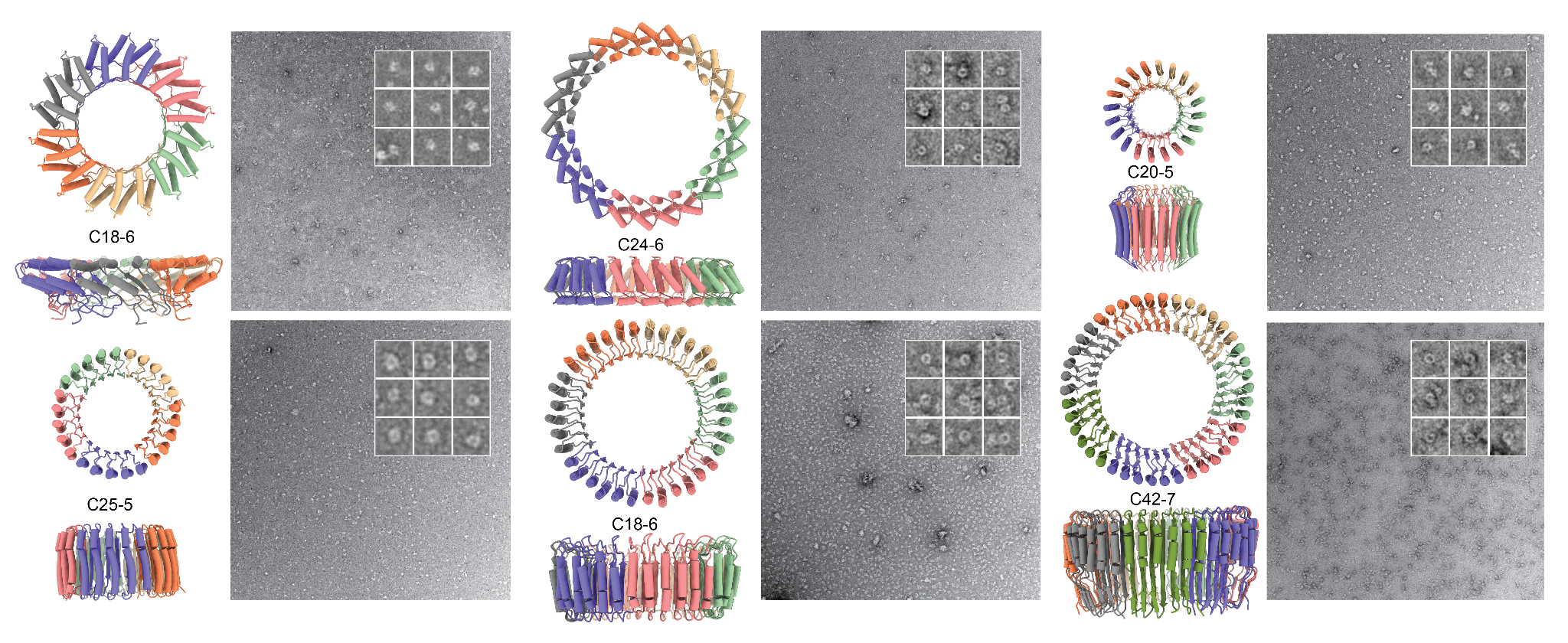


**Fig. S12. Design models and corresponding experimental negative stain electron microscopy analysis of designs shown in Fig. 3A.** A raw micrograph at 57k magnification is shown along with nine example extracted particles that were used for further classification and data processing. From top left to bottom right: HALC6_220, HALC24-6_316, HALC20-5_308, HALC25-5_341, HALC18-6_278 and HALC42-7_351


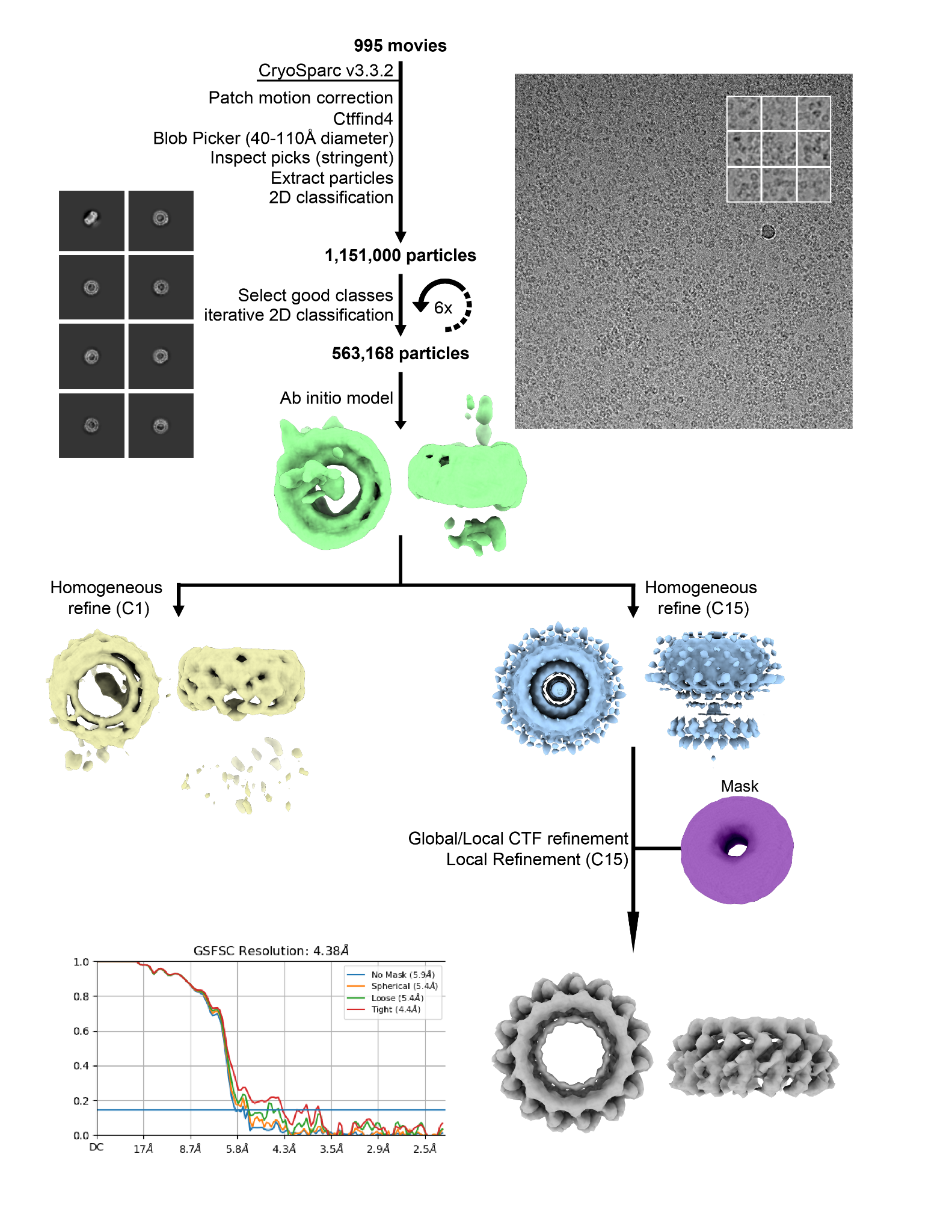


**Fig. S13. Details of cryoEM data processing pipeline used to generate electron density and structures of HALC15-5_262.** 2D class averages and *ab initio* reconstruction followed by a C1 homogeneous refine yielded C15 features corresponding to the size and secondary structure of the design model, which allowed us to further process the design with C15 symmetry imposed here. A representative raw cryoEM micrograph is shown on the right along with nine example extracted particles and characteristic 2D class averages used in the processing pipeline. An FSC validation curve for the final reconstruction is shown along with the electron density map.


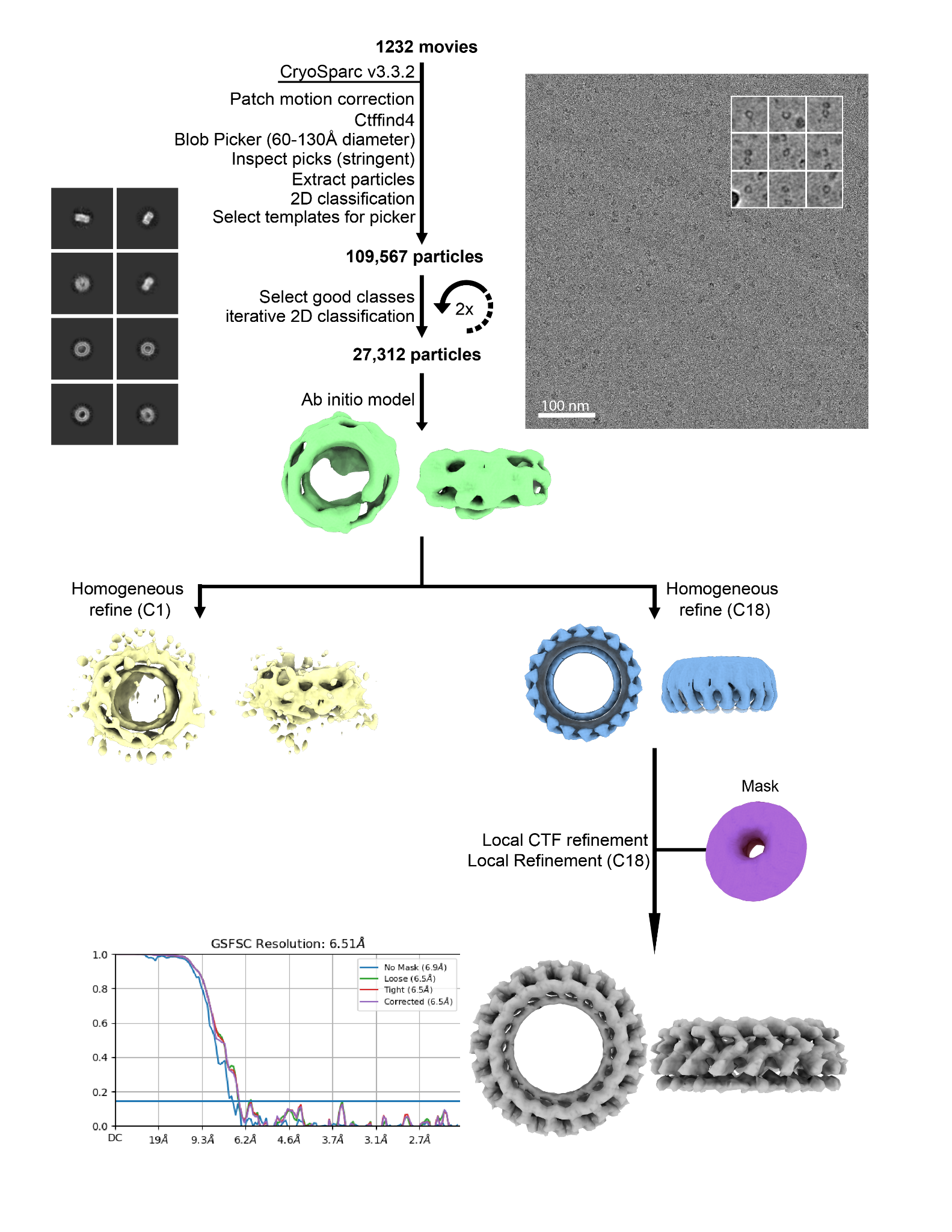


**Fig. S14. Details of cryoEM data processing pipeline used to generate electron density and structures of HALC18-6_265.** 2D class averages and *ab initio* reconstruction followed by a C1 homogeneous refine yielded C18 features corresponding to the size and secondary structure of the design model, which allowed us to further process the design with C18 symmetry imposed here. A representative raw cryoEM micrograph is shown on the right along with nine example extracted particles and characteristic 2D class averages used in the processing pipeline. An FSC validation curve for the final reconstruction is shown along with the electron density map.


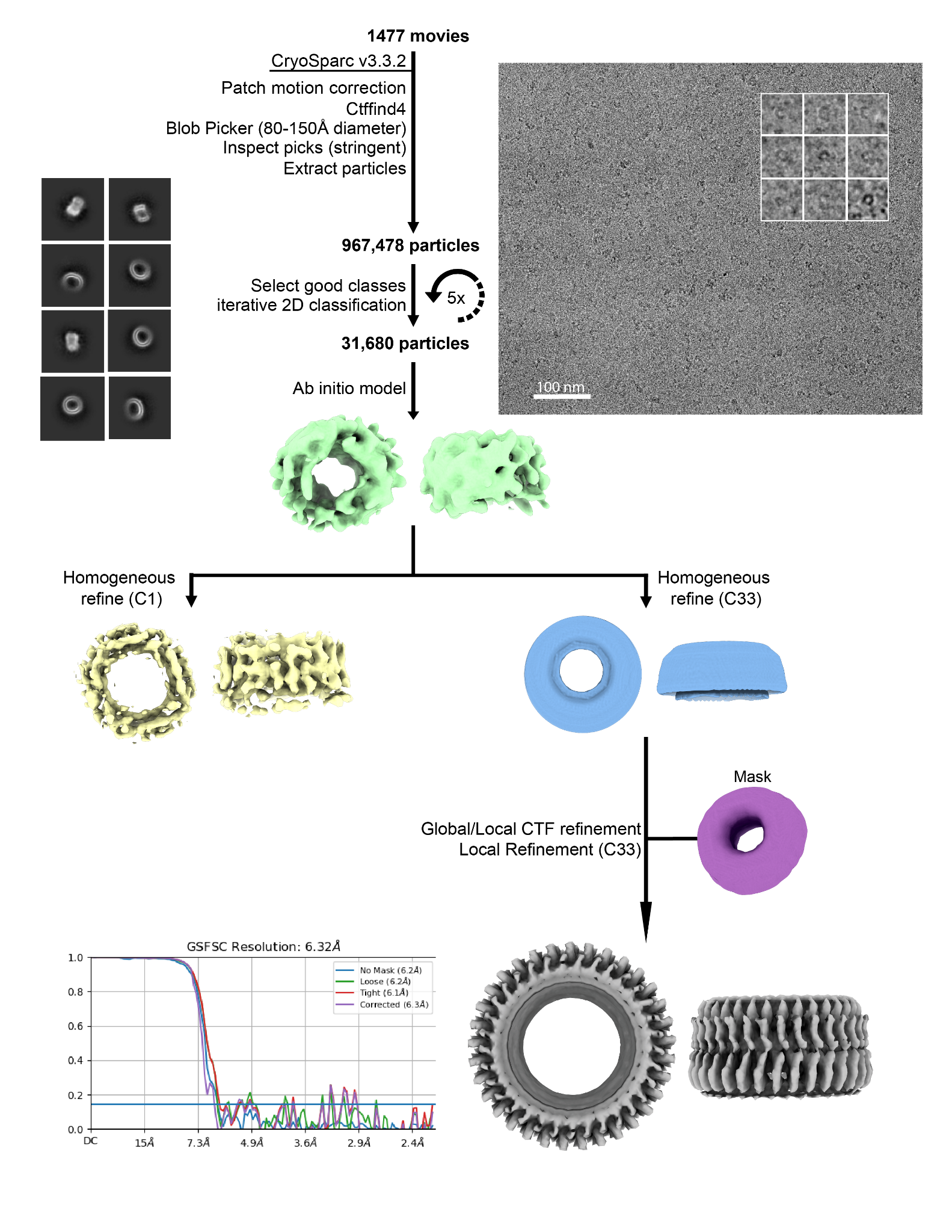


**Fig. S15. Details of cryoEM data processing pipeline used to generate electron density and structures of HALC33-3_343.** 2D class averages and *ab initio* reconstruction followed by a C1 homogeneous refine yielded C33 features corresponding to the size and secondary structure of the design model, which allowed us to further process the design with C33 symmetry imposed here. A representative raw cryoEM micrograph is shown on the right along with nine example extracted particles and characteristic 2D class averages used in the processing pipeline. An FSC validation curve for the final reconstruction is shown along with the electron density map.

**
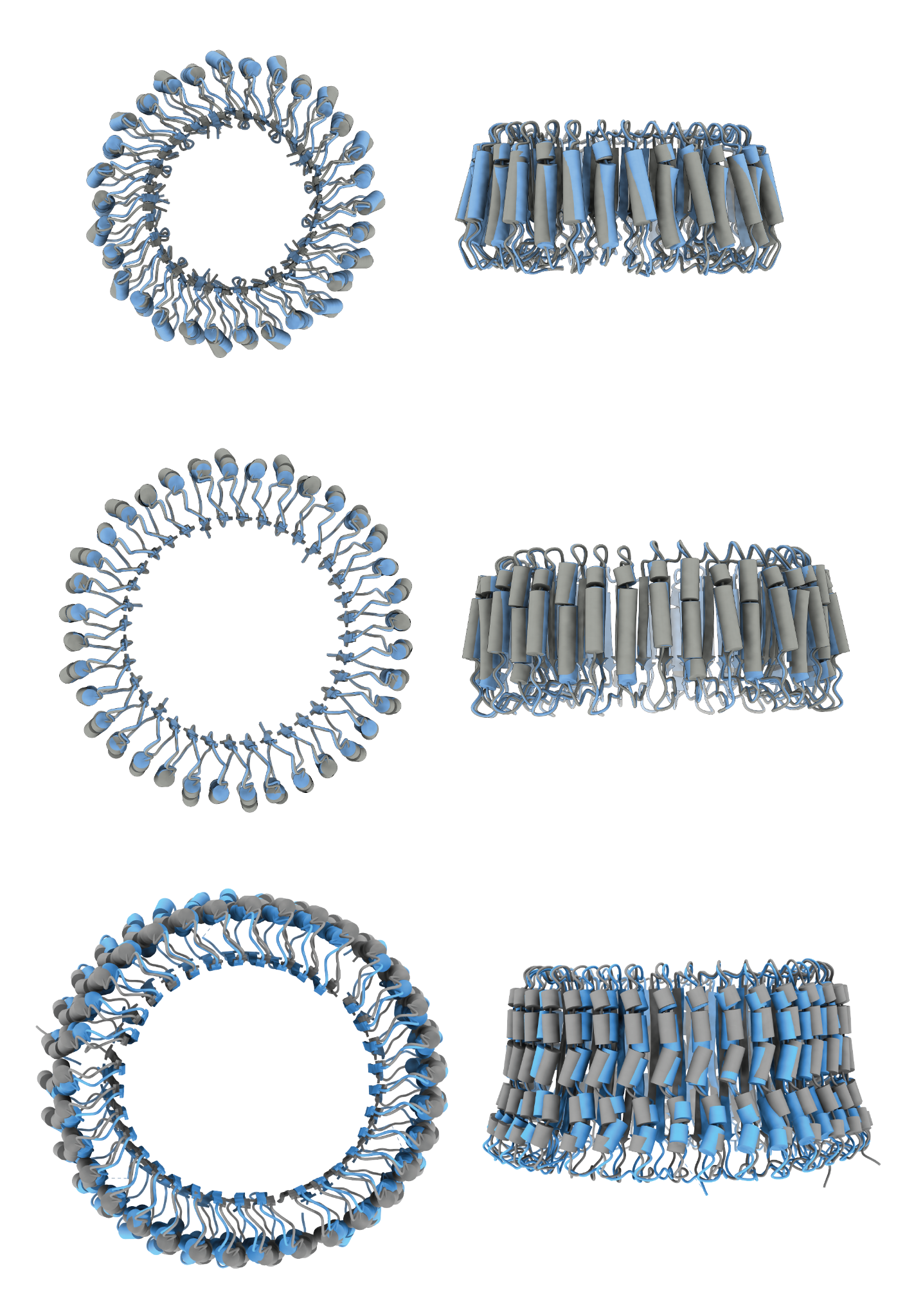
**

**Fig. S16. Detailed comparison of HAL designs versus cryoEM structures.** The designs were relaxed into experimental cryoEM electron densities using Rosetta FastRelax and SetupForDensityScoring. From Top to Bottom: HALC15-5_262, HALC18-6_265, and HALC33-3_343. Superposition of the designed backbone (gray) and backbone relaxed into the experimental electron density (light blue). The computed backbone atom RMSD between the designed and experimental structure are 0.81 Å, 1.69 Å, and 2.30 Å respectively.


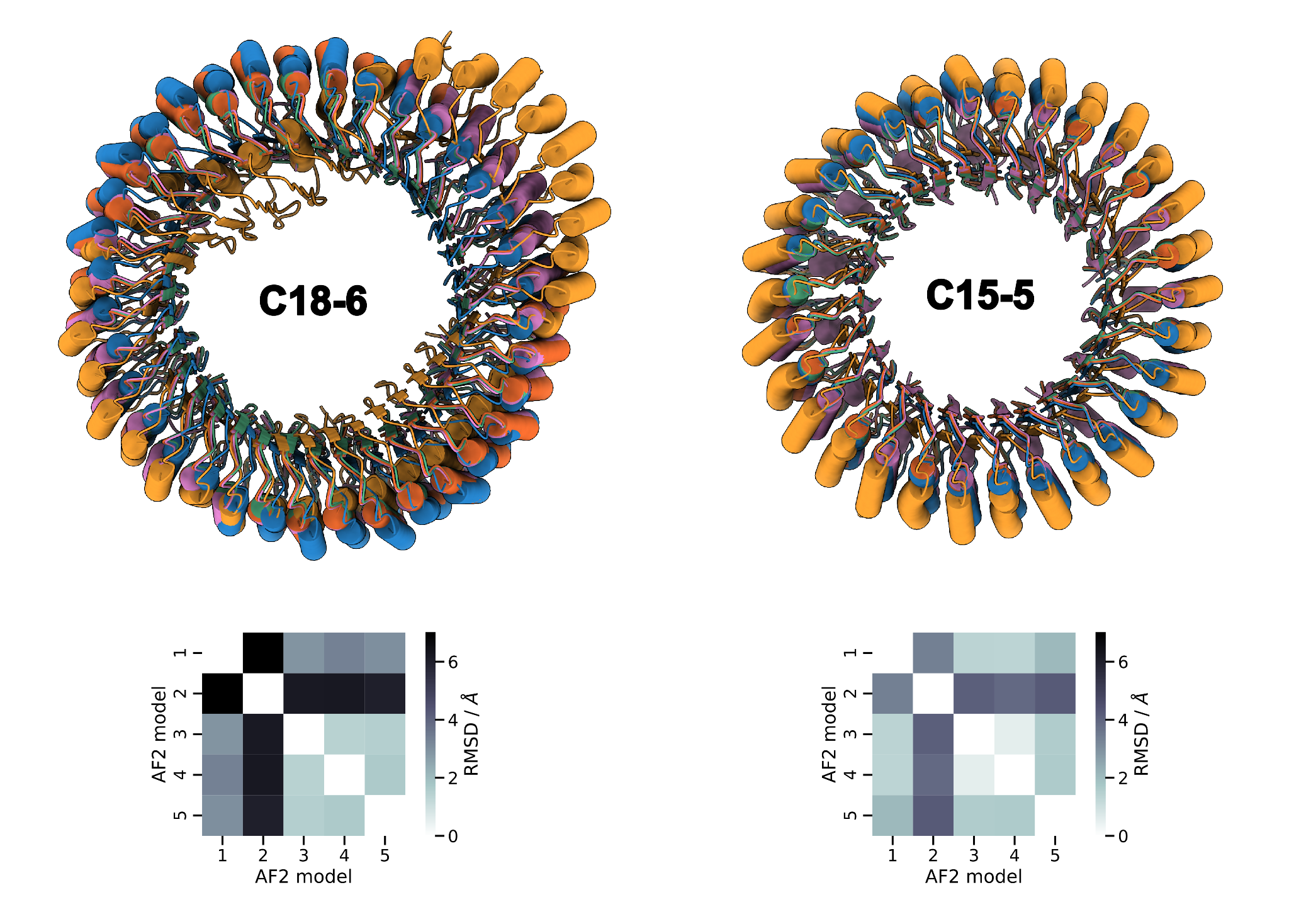


**Fig. S17. AlphaFold2 correctly predicts oligomeric valency.** HALC15-5_262 was originally designed as a homo-hexamer but the cryoEM structure (Fig. 3B) revealed a homo-pentamer. Prediction of the sequence with all five models in both the homo-hexameric and homo-pentameric configurations reveals smaller structural deviations and higher confidence scores for the homo-pentamer. Mean values for homo-hexamer | homo-pentamer; pLDDT: 70.57 | 73.88, pTM: 0.486 | 0.57, pAE: 19.59 | 16.89. Top: structural alignments of all five models (model_1_ptm; blue, model_2_ptm; orange, model_3_ptm; green, model_4_ptm; red, model_5_ptm; pink). Bottom: all by all RMSD values between the five models.

|  | **HALC2_062**  **PDB: 8D04** | **HALC2_065**  **PDB: 8D03** | **HALC2_068**  **PDB: 8D05** | **HALC3_104**  **PDB: 8D06** | **HALC3_109**  **PDB: 8D07** | **HALC4_135**  **PDB: 8D08** | **HALC4_136**  **PDB: 8D09** |
| --- | --- | --- | --- | --- | --- | --- | --- |
| Space group | P 65 | P 42 | P 32 2 1 | P 41 | C 1 2 1 | P 41 21 2 | C 2 2 21 |
| **Cell dimensions** |  |  |  |  |  |  |  |
| a, b, c (Å) | 67.9, 67.9, 228.4 | 50.2, 50.2, 22.1 | 70.6, 70.6, 31.4 | 107.5, 107.5, 111.7 | 136.8, 136.8, 94.2 | 35..9, 35.9, 438.0 | 52.8, 77.9, 52.8 |
| α, β, γ (°) | 90,90,120 | 90, 90, 90 | 90, 90, 120 | 90, 90, 90 | 90, 129.7, 90 | 90, 90, 90 | 90, 90, 90 |
| **Data Collection** |  |  |  |  |  |  |  |
| Resolution (Å)* | 56.95 - 2.11 (2.19 - 2.11) | 50.19 - 2.51 (2.60 - 2.51) | 20.39 - 1.75 (1.81 - 1.75) | 76.01 - 3.40  (3.52 - 3.40) | 72.61 - 2.09 (2.17 - 2.09) | 54.75 - 3.30  (3.41 - 3.30) | 23.62 - 1.90 (1.97 - 1.90) |
| Rmerge | 0.067 (2.197) | 0.311 (1.853) | 0.447 (1.368) | 0.076 (0.641) | 0.089 (0.684) | 0.148 (0.819) | 0.351 (0.884) |
| Rpim | 0.028 (0.878) | 0.089 (0.515) | 0.151 (0.496) | 0.037 (0.344) | 0.050 (0.400) | 0.060 (0.328) | 0.102 (0.244) |
| Mean I/σ(I) | 16.65 (1.17) | 2.85 (0.66) | 8.85 (1.33) | 14.56 (2.61) | 11.77 (2.06) | 9.29 (1.37) | 9.45 (3.95) |
| CC 1/2 | 0.996 (0.559) | 0.987 (0.336) | 0.95 (0.566) | 0.999 (0.748) | 0.995 (0.621) | 0.996 (0.639) | 0.981 (0.832) |
| Completeness (%) | 99.81 (99.47) | 99.90 (100) | 98.89 (89.99) | 99.40 (99.43) | 98.51 (99.27) | 98.67 (95.19) | 99.52 (100) |
| Redundancy | 7.1 (7.2) | 13.2 (14.0) | 9.8 (8.1) | 4.8 (4.3) | 3.8 (3.9) | 6.8 (6.4) | 13.1 (13.6) |
| **Refinement** |  |  |  |  |  |  |  |
| No. unique reflections | 34088 (3405) | 2002 (193) | 9287 (819) | 17541 (1749) | 20957 (2047) | 8009 (435) | 8869 (875) |
| Rwork / Rfree (%) | 23.6 (32.1) / 26.3 (33.4) | 24.2 (41.3) / 26.5 (34.8) | 19.0 (27.4) / 20.5 (26.2) | 28.4 (35.6) / 30.9 (38.3) | 20.6 (27.0) / 26.7 (34.8) | 25.0 (39.8) / 29.8 (43.7) | 23.2 (28.2) / 25.5 (30.5) |
| No. non-hydrogen atoms | 3210 | 469 | 563 | 6344 | 3159 | 2208 | 1056 |
| Macromolecules | 3210 | 469 | 538 | 6344 | 3159 | 2208 | 1020 |
| Solvent | 0 | 0 | 25 | 0 | 0 | 0 | 36 |
| Ramachandran favoured / allowed (%) | 96.52 / 3.48 | 94.83 / 5.17 | 98.41 / 1.59 | 97.33 / 2.67 | 99.20 / 0.80 | 92.58 / 7.42 | 99.21 / 0.79 |
| **R.m.s. deviations** |  |  |  |  |  |  |  |
| Bond lengths (Å) | 0.003 | 0.002 | 0.006 | 0.003 | 0.007 | 0.011 | 0.014 |
| Bond angles (°) | 0.51 | 0.48 | 0.77 | 0.53 | 0.92 | 1.37 | 1.47 |
| **B-factors (Å2)** |  |  |  |  |  |  |  |
| Macromolecules | 76.64 | 74.11 | 35.68 | 139.29 | 54.51 | 134.25 | 37.12 |
| Solvent |  |  | 42.86 |  |  |  | 44.17 |

*Statistics for the highest-resolution shell are shown in parentheses

**Table S1.** Crystallographic statistics and PDB accession numbers for the structures displayed in Fig. 2.

| **Design** | **Protomer TM-score** | **PDB** | **Oligomer TM-score** | **Biounit** |
| --- | --- | --- | --- | --- |
| HALC2_062 | 0.69 | 5J1P | 0.59 | 6IU4_1 |
| HALC2_065 | 0.67 | 5W8O | 0.54 | 1XS0_1 |
| HALC2_068 | 0.67 | 4PD6 | 0.57 | 2MFZ_1 |
| HALC3_104 | 0.87 | 7X8V | 0.88 | 5KA5_1 |
| HALC3_109 | 0.78 | 4AIN | 0.69 | 4MOA_3 |
| HALC4_135 | 0.80 | 7RTN | 0.59 | 5VB2_1 |
| HALC4_136 | 0.80 | 1W99 | 0.71 | 7KUY_1 |
| HALC6_220 | 0.65 | 7DPA | 0.51 | 6NYF_1 |
| HALC15-5_262 | 0.65 | 1YRG | 0.46 | 4I0U_1 |
| HALC18-6_265 | 0.65 | 4K17 | 0.49 | 5LNU_1 |
| HALC18-6_278 | 0.65 | 5IRL | 0.49 | 3FEM_1 |
| HALC20-5_308 | 0.59 | 5K7V | 0.45 | 4I0U_1 |
| HALC24-6_316 | 0.69 | 6VFK | 0.44 | 1HB9_1 |
| HALC25-5_341 | 0.59 | 5K7V | 0.45 | 2IUB_2 |
| HALC42-7_351 | 0.58 | 5AWG | 0.41 | 3J26_1 |
| HALC33-3_343 | 0.48 | 4K17 | 0.41 | 1DAB_2 |

**Table S2.** PDB IDs of the closest matches to the structurally-validated HALs (Fig. 2-3). Structural overlays are shown in Fig. 4.

| **Design** | **Repeat E-value** | **UniRef100 ID** | **Protomer E-value** | **UniRef100 ID** |
| --- | --- | --- | --- | --- |
| HALC2_062 | 3.70E+00 | UPI00131BD06C | 3.70E+00 | UPI00131BD06C |
| HALC2_065 | 5.80E-01 | A0A8I1R8D5 | 5.80E-01 | A0A8I1R8D5 |
| HALC2_068 | 1.40E+00 | A0A6B2M0S8 | 1.40E+00 | A0A6B2M0S8 |
| HALC3_104 | 4.70E-01 | UPI0013B3A05C | 4.70E-01 | UPI0013B3A05C |
| HALC3_109 | 8.20E-01 | UPI000B0DAB1F | 8.20E-01 | UPI000B0DAB1F |
| HALC4_135 | 2.80E-01 | A0A3G1RPF3 | 2.80E-01 | A0A3G1RPF3 |
| HALC4_136 | 6.50E+00 | A7ANS2 | 6.50E+00 | A7ANS2 |
| HALC6_220 | 2.00E-02 | A0A434I672 | 2.00E-02 | A0A434I672 |
| HALC15-5_262 | 5.70E-02 | I7LU18 | 3.50E-17 | A0A7S2JY04 |
| HALC18-6_265 | 8.00E-03 | W2S5F8 | 3.17E-16 | A0A7S2JY04 |
| HALC18-6_278 | 5.00E-01 | A0A7E5WBQ0 | 2.99E-08 | A0A819R934 |
| HALC20-5_308 | 9.60E+00 | A0A1F4XIB2 | 1.13E-05 | UPI001CF37084 |
| HALC24-6_316 | 1.00E+01 | UPI0019D624AA | 3.00E-03 | A0A7G8BM39 |
| HALC25-5_341 | 2.60E+01 | A0A6N1YEJ1 | 1.86E-09 | A0A2B4S1A5 |
| HALC33-3_343 | 8.80E-01 | D7MIU3 | 1.62E-35 | A0A2I0HQ60 |
| HALC42-7_351 | 1.40E+01 | A0A7L1D0M5 | 1.35E-14 | B4SHG6 |

**Table S3.** UniRef100 IDs of the best hits for structurally-validated HALs (Fig. 2-3).

| **Design ID** | **Seq. from** | **Protomer sequence** |
| --- | --- | --- |
| HALC1_001 | AF2 | ALVEHRTYQGGLHIVKLRSSQDLGNFIDVLHEYGALVELRNEPFSIDILAASDIVNRAFQNVQFD |
| HALC1_002 | AF2 | DPTAFYLRLLDYADADPTPRNIYGLLWLLDDSDLLIWEQITRKYNKAPVHTLFYKLIAELLRRND |
| HALC1_003 | AF2 | QLSPERLLFNLYGKLKNEFGVQVPEYMLRSVEATHQSVNLTWSKGAGRTGTAFWSDYGQFQAEDL |
| HALC1_004 | MPNN | MIVSLEKHPGGVHIITLSSEENLENFVKELKKLGAEVERLPEPNTVRVRAPEEVVEEALKNTKFK |
| HALC1_005 | MPNN | NEKEFLLQLKEELDKDPSEENVLSLIKTLNEEQKKILEEIKKKYPNLPLSKIFELLIDELLERLE |
| HALC1_006 | MPNN | DKIAFFKRLKEELEKDPSDENVEKLIETLNEEEKKILEEIKKEYPNEPLSEIFYKLIEKLLELSE |
| HALC1_007 | MPNN | MLSPEELLEKLKKYLKEKYNVVVPEERIVSVEATDSSVKLTWSRGDGREGTAYYSDEGEVRVEDP |
| HALC1_008 | MPNN | MLTPEELLERLRRHLEEEHGVVVPEERILSVEATPTEVTLTWSRGDGRTGTARYTSDGRFEVEDP |
| HALC1_009 | AF2 | KIVRALAALDVPRFLLISFPEVFGNKQFFLPIIIRPDLTEWSLKVELRLVEPGIDKITRLISVHEDAQDSVIVVLTLFVDVSVHRKIFQITDVFRFSTEL |
| HALC1_010 | AF2 | EPFYITGISNAGGFMFLVYEMGESPLNAIAEETKVQTGDTRLRIKDGLAEIKVKDNQMEILAVRGDNGAQFKISERAATQQLGSAGDRARSTVLINRIEK |
| HALC1_011 | MPNN | MDFYVTFTVNENGFTVKYHEEGEDPENYTSELVGLTEGDYTLEYPGAKIHLHVENNKFTVKSVEFSDNKKVKIDPEQRTQDVGKPGTDSKQTIHFHEILK |
| HALC1_012 | AF2 | EYIELNITGYLKVDEVNNTLYINYTELEELGLEYTTKVNLTNNTITITLKFNELEFETEITAKIEITDENGTVFTETTETTETTNTTDITITNTNIEAGNVTIEKSIEITDKNGTLNYTSSITTHIESGE |
| HALC1_013 | AF2 | EMSTFRIYKDTRRDALEAFYWEALFATKKESYQVVVRNKQLAKDIAMGISTIGHLAQGELHELLILYEVAMNVDLDSVSIMIVAESSGLVISVGVFNSLGLEAAIVLKLSNYADFVKFFEALSHNVTPTR |
| HALC1_014 | AF2 | GPIFRWPALEVDQLYGFKIFLKDIGSVQVEIEPNVDILVGLYYLREEEMIVDNTQFARKGMMVHLDPATDISFYPATIIHGSIIGEEIQGLDVELSLKVKVGYTHLFIFMSGQLYGAREELVFTIRVRNG |
| HALC1_015 | AF2 | HYRLEVYIREPAANNDRWELMIFTRSLSSSVPSRKEATEFGLVFAIKLTNMLEYSFTDTRFYLYQVKGYQQSVPRGGEVINFAVVEVKYLIDIHNIKTEHIPYLLLSVVRSVGDAEGLEIDGFKLLAPFE |
| HALC1_016 | AF2 | EHYAYLLIVVLTDHALDATAAYAKIAAESGGQELSFYYGGKMFGKIDFKKHSIALQGSTWAGQIARGNIDLTIVLAVRVMADKVESAISQVIYDEKIFEKLFIPWDGTAMIGELYTNDKLWGKLLIIKGV |
| HALC1_017 | AF2 | YASITLLQKPNGGFKNFGEDAEKVHQNKNRSVPFSINIVKATPNDAEANPVVGAHHNLSEKASNQPVEQAWDVFEHLDVVSELDAGEFSSQEERAAAIRYLAANIRDLKTPEMRSLVLVAFEGVYKQMTE |
| HALC1_018 | AF2 | KGFDYAFWIDSYKISTIPKTGSKYGFASRTRNEISAFIEMKFPDMPLLRIQVQQYSDYHMRIEAELDPPGSQADAMFDPKKTEKGAFAKGKFKMKWRGDRKEFRVLWSINPAPKIRSIVILVHALTRGGK |
| HALC1_019 | AF2 | IFRVQGQVRVVVPLLPQHRGYLDFSLQHQDTVETPPFFASIQNSWSSIEKVVSELKGKVIARVARLEGEEAITTDIANNIHRSFAQMRVDLAAYEEVYKGQPEPSEIYFKYVTPGLDIFELSLGVFEVDK |
| HALC1_020 | AF2 | TGLTIDFVGHFTPAHFSIVVGIREYQGNQKTAYRSEAANIVSHPDNNKIAIKAEGLETAFMPPNSIFVLDEANFFEVIQRGMNGQQVEQAPEPVEDRNGYIVFKETDVLLVDGDGQQRPATHAHLYFTFS |
| HALC1_021 | AF2 | QVIVVALDWYFVTNKPGFASVNLYMVWNDRERTFTILVLIYLRDIESKRGTFYMKLKIYGQRANDIFLWLVADRPIEIHLSLQVEYVEEDMDYHLFMRWKAFPRFLTVARVHTDHWEITPITTIRLEQLP |
| HALC1_022 | AF2 | RFRDSANVESGNASAEGDVNREFVLTLNNKLVAAVAANYRFMLGIRTIPRVGRKTTDQIDIRAPLLAHQKTKKPQDVGIMNEKYVAPGAIRSVTIVLNGGVSAVLAYIFVEGANHNLVGMALGTAHYTGE |
| HALC1_023 | AF2 | EIVLEFKIEWYLIGHQQRFIILMYYKLRHPAGFDYHHYLTAWEFFRLKKVPAVIIEWLVQLSETLGMVIDQESMLRFASFVQMKLAVVLKVASEEFLLVLDNYHAQAGFKIKVDGLDEIDIVVTVYASSL |
| HALC1_024 | AF2 | DAKVLAIEVKDDDGEMRTSNGEMLGGDTRQGIWASVAIANVGNGGPNRKHYALAIALQGVGIPLGQNKVLLVIIFMDNMDNVMTLVILELSKEGRIKYLSNQGAATTIEHVEGREFVSFDSKLVRIWQII |
| HALC1_025 | AF2 | RIEGAFQLDEVREVGRAGDYLLVFQFVVFHDTFLHRYLVIILVRGVQNRDAMLWFHASDGEGGNKVFVTLQAEDGQQLVVAMVTVVGVGADVQSVEVIQPANPKGNLGIEGVIALNKDNKTPKDSGRVVV |
| HALC1_026 | AF2 | PNEALVILIEFEVESQRLHWRPLYNGVGKYELRTSKPDRKLFVNWNVLPSPQAKMLFELIAYQDRVSKDQTLTAIRIVIQVAVEDSVFGESAVDIKQPGKIKKVHVKVGEDGMEDTERLNAIYVFIVHQF |
| HALC1_027 | AF2 | PRGKVLLALNRSVDDLLDGEDNLLYNLLQIGFRDFGYVAIRYRANLGEHARLNQALFEFVSAKDGLWNHLYINAMHGESVMRQTKESIGVQLDPLRGFIVDIYGADVFQLREAVDVLAIIPSWRFIILAT |
| HALC1_028 | AF2 | EGHVVMLDINTTTLAYRRAELIVFLYNWAFWAQASELDLATVTINIMGAEEDRLTFSIAISGNKSADAAQALILFDRELYLFDIVARRQSYPSLVISGIAAVEVSAENAKRVLSRFVAAGKRVRLGTAKA |
| HALC1_029 | AF2 | FTALFIVGLPKDSLSRDIPNPGYAMAIGLYTIAEVSETPEPGIEKAQITIDFMYSDPRLFGNVEIRLIGVTKEDERGRFSTQVYITITRYFADGKSLGAKEDLRPHSFKTGEANLVSLGGDFITLIGFGF |
| HALC1_030 | AF2 | VDEVAYNEIMKIHQKAKAADIGVPKEGEGKRINGTHEQSSAEFYINGYLLNPFIFMGVVYDNPGEYDLYKVEWDMENHWDVTTVRYNHDKNDRSVATYQAEHIKIQLGTELLKDKVLIFAVMITLKDRVN |
| HALC1_031 | AF2 | KRYWISLRNPQQPRTNPIVGTRRIDLMNEGTQRMLFLTVRVLQYNGLVPDNKRIDLLVSATKHSFIFADIRSTLTPQVWFRVTLFAQGQGLRGAKIMEAFSIPNQLRVFQFQSWNIPNFTILNSYLDDSI |
| HALC1_032 | AF2 | TRDQFAVQVGYGGVHRPDVGNLILTAVESKLPFKLGSAKIREGVVALTRKILSGSNVKDDAERQGHIIGFVKGSYRRSDAFVIKINEKEIIIIAIHEQRDSVRHMLHMLDSIAYGAINTLFSKVKIVVVK |
| HALC1_033 | MPNN | MVTTHTIVESTKEDALSALKESSRRVTEQERYVVRVTNSKLAREIALAIAETGHLADADPAELEKLREVAENEELTSVEILIERTPEGIRLTVKIYNEKGLLAEITLFLRDEESFDRFFEALKKAVKEIE |
| HALC1_034 | MPNN | MITTRYITTSTKDESLSALRDSSKTVTPETRYVVRVTNPRLAREIALAIAEHGHLADADPEKLEKLKEVAETKDLTSVEIEIQRKPEGIELTVSVYDEHGKLAEVTLFLRNQEQFDTFYDALEQAVKELS |
| HALC1_035 | MPNN | MKKLYEYTVTTLDEFLEKLKEFILNTSKDKIYKLTITNPKLIKDIGKAIAKAAEIADVDPKEIEEMIKAVEENELTKLVITIEQTDDKYVIKVELENEDGLVHSFEIYFKNKEEMEKFLELLEKLISKLS |
| HALC1_036 | MPNN | MEMRYLLIAVVSKKKLDSTQAFEKLKKLSGGKELEIYRDGKLIGKIDLDKRSITLFDTPLEERQKRGEIYVDLVKVEEVPKEKIDERIEKVINDQSIWDELKQKVNGQYMVGEVYIDGKEFGKLLLVLAE |
| HALC1_037 | MPNN | MEMRLLLVVVASKRKLDSDEAFEKLEKLSKGKELEIYKDGKLIGKIDLKKKSITLYDTPLEERIKRGEIHLDIVKVEEVPREEVEEKIERILSDKEFWKELNLEIDGTYLVGELYIDGKLFGKMLLLLAK |
| HALC1_038 | MPNN | MDKIDITFDFTWIGKDNKFKLKMTVKVTKSDGTVVTGFEREYDFDSLEEVPEAIEEFLRDVAEHLNLEIDPKSVKEFAEKVTSILKEMLKEMEENDPEKSKNIIRKYKFTLELKGQEPVTVDVTLKATLN |
| HALC1_039 | MPNN | MNDIHITFDFTWSGYDDKFSVNMTVEVKKEDGSVVTGFSQSYSLSSLDELESKIEDFLKSVADYLNLEIDPESVKELAKYIKDKLLEMLKKMKENDPEKSSSLKEKYEFTLKIKGEEPVTVDIDLKATIS |
| HALC1_040 | MPNN | MFEGEFRLSDVEEVGEPGDYLIELEFTEKVGTTEYKYKLRILVSDVKDLNSVLHVSVSLGEGGTTVYVTLEASPGQKITVREVRVEGIGANVEKVEVIIPKNPKGELTVERVVALNKDNEVPENSGEVIK |
| HALC1_041 | MPNN | MKPVKIVHLDKTVEELLSGDNPVLKQIIDEGLEKNGIIAIRLRANDEELETLVDWVKEYLDSKNNKTDLMRIDARDGRSVREQAEEWLKKNLDENKSYIVVARGADDKQLEELVEVLSKYEKLKFVVIAT |
| HALC1_042 | MPNN | MKPVRIIELDKSVEELLSGEEPLIDQLLDEALEENGIVAIRLRANEEEIETLVEYIKTYLDSKNGKTDLMEIDATDGVSVTTQAREWLAQHLDPDKSYIVVVRGADSEQLRELVEVLSQYEKLTFVVIAT |
| HALC1_043 | MPNN | MRVTVNVYIPSSAFPSSLPDPGDAVATGVVTLDEVNEERQEGKVVDTLTIDFTYDNPRLYGKVTVTIIDVVRLDDEGRYELWVKITVETYLSDGTSLGSITNDTPQKVRSGETVTVDLNGIKVDLQAEEE |
| HALC1_044 | MPNN | MKLTVTVNIPSSAFPSSIPDPGDATATGTITLEEVSDEPKEGVVKATLTIPFTMDNPDIYGTVTVTVIDVKKLDEDGTYELKVKVSVTTYLKDGTSLGTITNDTPQTVKSGETVTVDLDGITINLKAEEE |
| HALC1_045 | MPNN | MKLTVTVSIPSSSFPSSLPDPGDATATGVHTLEKVDDERKAGEVKDVLTIPFTFDNPRIYGTVTVTVIDVQRLDENGNYELSVKVTIEVYLSDGTSLGSFTNSTPQTVRSGETKTVDINGIKVDLVAESE |
| HALC2_046 | AF2 | ADLVKILVPKIAFKEAQPIVLEFFRRRRMERHIMEFYAHFDHTHPNEEVNNVYDVSGEIITTNLE |
| HALC2_047 | AF2 | QFILITRQTVIAHARAASAHLEVGPAEQIVPENRIYLIYFYRGRYLVIGIVIFDNARPIVWRLEI |
| HALC2_048 | AF2 | VKRLRVEVHNVAEFKLTIIDNGIGTRWLEKAELAIFGAIAVFGQEALLPWEVQLVLDVFVRKRTG |
| HALC2_049 | AF2 | KGVLQYTYERANSNRFTLFFYTQYFVVGSSEAEQLADTLIGEWVRALSELDFVDVRHETRRTNIK |
| HALC2_050 | AF2 | MLTFPAGSLTPENTRSQYGASFPAYIVRDVLRAINQISISREKFPVQYKMNDNSMFVIFKYSPRL |
| HALC2_051 | AF2 | VEVYDYQALRGNELIAPIALVESEPTVTFQIVTPVPFNPMDILLAWLVFLFNTGGDRRELPTLQS |
| HALC2_052 | AF2 | PMIYHAPYTHTGQEIVVVIGSTNLFIRVGDDAIVFDNRRYSFRETVTEEDFYNAETHEGIISLAK |
| HALC2_053 | AF2 | AHFKATLSRLDSIAADGFSYMSKIAANDPGLLSTTRFPGEIILTYHNTKVGFDLIISIAATETHF |
| HALC2_054 | AF2 | EKSTGPVSIRLDKEIEKNGEHVRLEEVVISADSRTARIEIAIRHSGVQITTNHLLEAFIQAAAVL |
| HALC2_055 | AF2 | KLKLIGEIETTDAFFFARDLAARTNISTDSLLGFGKGFQNFSVKFKYAGPQFLLVEVRLLGVIDL |
| HALC2_056 | AF2 | VLNLDETDLMMNMIMDEILDEPKDAISPVIIIYVGSAPFLGAMDKTLNRLGDAYGNIKFWMVFHS |
| HALC2_057 | AF2 | LLHVNIIAHAIIMFVEQYTVGSSDPSIYAEAAYTMGTRFREAPWIKKEELRLGLKNSLDYIVLFI |
| HALC2_058 | AF2 | PLQRPEDIGIIGQNLTNHGLGSDRLLEKGSDLIPSIRISHLMIIEMKRRMDIAPEFLFKKLKQAL |
| HALC2_059 | MPNN | MVKPITEEDVREAATAASPDYEVGEAKLIDEENNLWFVTLYKGDQKIYALIEDKNGEFTVHQIEL |
| HALC2_060 | MPNN | MKRLEVEVRDVRERRVEVRDDGVGEEVLELARQAMERAVEIFAPLAQQGLRINLRLEVEVEPVVP |
| HALC2_061 | MPNN | MATVEYSYEQIDETHYRLTFHVRYEYKKSKESKELAESLVSGFVDALSSLPFIEVHYSVEEVEVE |
| HALC2_062 | MPNN | MARVEYSYEKLNDTHYKLKLKVTYEYRKSPEARRLAEDLVQAFVDALSSLPFITVEYEVEEVEVE |
| HALC2_063 | MPNN | MKVYEFPYPETGKKIIVIQGEKNIVIVVGNTAVVYYEGKWTYKENVTEEDIEKAKTEEGAKELAK |
| HALC2_064 | MPNN | SKLKEQEELIDEISEKAKEFLLEIKEKYPGELSEERYPGRVVLTYVNEEKGFSITVTIELLNKEK |
| HALC2_065 | MPNN | SEEEKPIVIDLNKTIERDGRKVKLVRATITVDPETNTITIDIEYEGGPITKEDLLEAFKLAASKL |
| HALC2_066 | MPNN | PLNLEETKEKVDEITDEILSRPREEVTPVVVLFVGKPEYREAVERFIERLTERFKNVLFYEVEVG |
| HALC2_067 | MPNN | DKLVRVLSSSMIYYAERMTKGSTDPSDYDKALDDFYNYFLEQPFVDKETLEKAYELARKRLEELL |
| HALC2_068 | MPNN | MIKVPEDLERIGRELRARGLDTKRLLEEGPKLYPELSIPDLMAIALYDHLNLDPEFLYRLLQQSR |
| HALC3_069 | AF2 | IRELEGKIAKIERTLGVLEATVVYLLTLFSQETLINIFMNTWADPSVDKAELNEFIHRVLERKDK |
| HALC3_070 | AF2 | GAHGEILVYILRVLGIAFTHFFIPEFENLGLHLLGLLMKDSPGSFEAALQHNDYSRYLYEKAKSA |
| HALC3_071 | AF2 | IKRKIAQTTSEFLGMENVGKDRTFITFQLVDEHLFFITGKQDAWLYNQFIALFERQGVLSRELIE |
| HALC3_072 | AF2 | ELQPLIANIEVFMLTFEDKRNHFIQEGKLKVLTLYLGLVARKLAKFILQLSYLLERAYQKIDALQ |
| HALC3_073 | AF2 | KLGKILQSISSDPVLLYIYIDKLLYDIEAIGGGTEGIEVFLQTVIPSSIKSHAYKVHQVRSEIYR |
| HALC3_074 | AF2 | LWTEIDRLVSSIVHEMTDSLGRTLALQFYELYVLMRYQRFPSQDKASVAINYPDGRTLRNVIRDQ |
| HALC3_075 | AF2 | NKVFHILNELRYIRQYIYFNIKLLSTLLNLLTLVAAGQPDSDSYDQGLSNAQSYLRQASGAARKI |
| HALC3_076 | AF2 | LMSVINALRDRVARLPLSEVAENDSLDEDLAGVLSSYFDNDLRDLNMIVRRWRRTDPVMIFAFLN |
| HALC3_077 | AF2 | SLSGVKGRMENDLLHIYEGLFPGSEDRARHGFRRMEAEDNAWLVGDEILKLVSGLYDFLIYPMLK |
| HALC3_078 | AF2 | IAPSELYRLKMYFNIFKNDLAKPLGLTLILLLAIFDRNNFKKTTKLLEMIYWEKGHHRIGAELSR |
| HALC3_079 | AF2 | TRNLLERAAPHLNAASLVQEKISSNKATSAMLFSAVHNIIKIIKFLYEAAGALHNSIRTLQKQLA |
| HALC3_080 | AF2 | VAATYGLILFAIARSYPQASRNLGRILLAYGYDDSLVVGKEMLELVNSRSATIERIGRIVVPYLD |
| HALC3_081 | AF2 | MLLKKYRVGIDELARSSASLRRENGLGKTRLFKSREGYKVMGDFEKLHFFVALVARRLLTLEEDM |
| HALC3_082 | AF2 | DNIGEFREIDGTSLLIILYSDGLDEERRTTAIKPMIELVHSERRLAFGVLDRTGRLERLIVRTLG |
| HALC3_083 | AF2 | KKVIKEAVLKSEIRTIGVMHLAFESVANHEPLKELYGKFLSAVRKAYSSKQRKDVRSQKMRIKSS |
| HALC3_084 | AF2 | SMMGAYFLLLVEWAELLPYLARDYVQPKLTPYLIGIQKHYIALLNGSKDNPAYTNIFGKDRAFTV |
| HALC3_085 | AF2 | IKLKDAGIAYAGVHFFFREPKNDNVIYEAGYRQDASQLVVHKRSRGQINKLFGNMWQNVKSLPDN |
| HALC3_086 | AF2 | LTWIYLNLRRLWEEQEQIPGAEILARALELGILVTEALMNIYKAVKDINQQILAIRITIKTIEVA |
| HALC3_087 | AF2 | NDEAKRLYHRATNGVQVSEIELIIFASNIQRKLDDFEKEVMQLIGGLRYALERVWTLTLEILLNN |
| HALC3_088 | AF2 | QGRYLPGMTAWEEEDANIISELLPGLEFMKVLRKGGKRIFLANHQASLIVVAFDWITNAKLRSEV |
| HALC3_089 | AF2 | DKPKAQENEPIAGSVGSGKLDDMMYLKVRGWNTVFLAIDPNAIPFVQGLFSEEDWAERVDMAPLN |
| HALC3_090 | AF2 | MTTLKKWAWDRETATLFIELELDTTAYMTLFLYTSENDEALEMLEAREKEAATDPAITLIFGYFS |
| HALC3_091 | AF2 | GELRLKQEIFFLYDTIARLLQRLLTYLFGNESFWREKVDMIITLRGRDSQARRLLLTRLVEKIPV |
| HALC3_092 | AF2 | IDKKFYIADIKIINMQLKEIWRRVQQIEAEVQRVYLESMTAITILYLEFRVAYSELLSLLATLRA |
| HALC3_093 | AF2 | STAFEGAVARGIAFSAIGLLPLFGTQSQYILSHIKLSMFRPGSREKVAKDLYDLDDVIYELIHTM |
| HALC3_094 | AF2 | AFDWQSPENIEVIFDLAVIGVGAHFPDENVLREFYAGVERVSLEGWVLHRNLYNEDPMGGAQLAK |
| HALC3_095 | AF2 | PILNYQQFMSDAISEVQKRHGQKAVEQLSTGEASSDVLISLVYSVAARLVGYLNFLSGNLQTIGN |
| HALC3_096 | AF2 | LNKPKLAAIAKPKSKGFYGDIVLLKESDNQSEMLQIIYVPDIFLDWLLLRLEDDNWQNFNDGPEQ |
| HALC3_097 | AF2 | DLNELSERVISLEDNIDQMVHAIIFLGANLAGIIKASEAGLQKLNDLEHQRELRRLYNLLNQFWS |
| HALC3_098 | AF2 | GTVVDRYKDRGVNIYDVLREEVNLIVEISKQRIDGSGKKAVDYSMKLLFLIEHIITMMVEVLSRS |
| HALC3_099 | AF2 | HQFELAVANDIMAKAFFSIEVALKAAYLLGQLLYPLLGRQFMERIRYMITLIKSEVYEAAARGDM |
| HALC3_100 | MPNN | LEELKERVEQLEKRLSVVESTLTHLLTTFSDETLKWIYDNTRADPSVDKETLDEFWKRVEEEKKK |
| HALC3_101 | MPNN | RVQDIAQAISEFLGMEGVGKDNTKITFILHRDTLYLVTSKEDPELRNRFIQLFVDKKVLSESQIK |
| HALC3_102 | MPNN | KVDKIAESVSEFLGMPGVGDDNTKITFILHKDTLYFVTSKKDQELYDRFVDLMVEKKVLSREQIK |
| HALC3_103 | MPNN | KEEDIAKTVSEFLGLEGVGKDNTKITFLLHKDKLYFITSKEDEELRNRFINLFKEKNVLSEEQII |
| HALC3_104 | MPNN | KRIDEIESKLKHLEEFTTHLIKLMETMLELLKLVSDGKSDSEEYKELLEKAEEYLKQATEAAKKI |
| HALC3_105 | MPNN | MEPEELERLRELYEVFKDKLDEPIGLYLLTLLAIYDPERREEYLEKLRDIFEKQGETDIAERLKE |
| HALC3_106 | MPNN | MAAVFGLLLFSISSSHPEMAENLSEILLSHGFEESVEVGRKMVELVNSNEPTKEKISEIVVPHLE |
| HALC3_107 | MPNN | STLEEYRVGIEMLERDAKEYLKENGHDSEENFRTEEGYKKLSREARLMYFTALTSSELLRLRQEL |
| HALC3_108 | MPNN | SELEKYKVGIDQLYERSKEYLKENGFESEENFQTKEGFEKLSDEARLMYFTALTSRTLLKLQQEL |
| HALC3_109 | MPNN | REEIEEAVKEAELKVLAIVLVALRSVSHYEPLSRLYESFLDALKKALSEEELKEVEKEAERIEKK |
| HALC3_110 | MPNN | LEQILEELTELLERVDEIPLREALKRMLELLVRVTQELKEVKDKVESLEKHLEELDKRVEEIEKK |
| HALC3_111 | MPNN | MAKLLPGLSEEEKRLTDILDKLLPGLEVLDVLREDGLVVFLARHGDHLLVASFTRFKDPELQSKV |
| HALC3_112 | MPNN | MTKLVEYHYDEETQLLYIKLQLNENEYLVLFLYSKEDEESLKKLKELEEEAASDPSLHLVKGFFK |
| HALC3_113 | MPNN | HMEEMKKQIESLYNLLADLLRRLLTYLQGDEEHYQKLVDEIHSLEGKDWEKREKLLTELIDQIPV |
| HALC3_114 | MPNN | VDEKEVKERFEEIESRLEELESKVREVEKKVEEVKKESDEKIDQLKTEFETKYNQINNEINTLKN |
| HALC3_115 | MPNN | MLIDFDKFWEEAIERVREELGDEAVEQIESGNATSKTIIALVKAVSELLVKYLNEMTKELEQVKK |
| HALC3_116 | MPNN | VAKPQLYAIATPKKEGIDGRLVLLKIETPNETLLQLVWVPDVFLEWLLELLEDDDLSKFTEGPKE |
| HALC3_117 | MPNN | VVKPKLYSVAKPKKEGIDGNLVLLELKTPTETLLQFVYVPDYFLDWLKKMLEDDDLSKFTEGPEE |
| HALC3_118 | MPNN | MTRLEQLLAQGVDPFEVLREKIEKLKEIWKKYEEAKGEEKERYRDELLKLMMEVLELMVELLSRR |
| HALC4_119 | AF2 | MLRAIRTAGKPDVKEVNGYLKSLWATVYKRKDTFWILAMFIKAFHSYLAKLKLDKFDVLTDIITN |
| HALC4_120 | AF2 | AKDYEINLIRKVKAVILQVATDVLSYLKKWFLVLAAVVVTKSWDYVAELEKAVSVSINELKGKAT |
| HALC4_121 | AF2 | EGAFHDAIEITTVLLEDLLGNDDLLGKNLGGVAVLIAQLAFVRANEGTLDILSIIDHINLFKDGM |
| HALC4_122 | AF2 | SMQDVALFARIDTLTRLVYNGVTDILDMLAYLDKQIKSVYKYTVAMFALLIILLVLTIIEFLAWY |
| HALC4_123 | AF2 | QDYSVTALTKHVFTRLTDLNQRIAMGELKILTQLEKLTGFILRLIRMLEEAYLLLVTFYEDFING |
| HALC4_124 | AF2 | SLEGRLVDIYSTMSYIFFRIKAMLQQITKRIDLIGKREVGLENYFLNEALGQPEKAIDVALSWKL |
| HALC4_125 | AF2 | AVRAIVKGANYGTWVKDEREALRNNVKSLAGADDQAVPPVGEINFGQIHELVFAFIAALVVRYEK |
| HALC4_126 | AF2 | QGLPVLIAVSDLILGRATLKYQWKMSTWQTYDKSFSRKKQEFLHEKSLKEMENAISKFARNAQQQ |
| HALC4_127 | AF2 | GMFEDIGIKNGTRLGITGEDLADKIARMATNFYGFPKVADVIKGSGWTGEALYSSVPQILLNLAV |
| HALC4_128 | AF2 | VEYSLFVIYHGARQTLLNLPVHQSKRLRYSVHLHEFPYNLEGIVADMEAFDMYSVYLDRRSRAFL |
| HALC4_129 | AF2 | VLPESIQTIDKDVRLTLLMGLMYLSGVSLVQGHVALVIIAVTLRLTTSDLHFLVATHHEIAKTHV |
| HALC4_130 | AF2 | VMNTIQTAHAEITDKLNEVEANVLRRLHQIIYYILRILELLDNILDLLVDILTLTLFLKNQAEHN |
| HALC4_131 | AF2 | MDVNKLVEIDYQDVKGTGMVRKMGLEGFRSKVDLLLSYINKFVPFADHKVGLFVLVDILIMLVYD |
| HALC4_132 | AF2 | PALENSLWILAVLWQVMLEEDNQRRAKVLGKALMEPVARRKDPKAAMLITEGEKRNPTIIFPGIE |
| HALC4_133 | AF2 | GVPRSVDLIRTHDSAKTDLQEGIEYSEAHQNHHGLYGSITLMARGIRRIGDYLQNGFSLLLPRLG |
| HALC4_134 | MPNN | FLREIRKRGKPDVKLVKDYLVELQKKTNEDKNEQWKLDLFLLALSAFLKELKLDEFEVLSEIIEE |
| HALC4_135 | MPNN | MEKFKEQLLEEVKKIVLETMTKVMEHLEKWFVTLAEIIITKSEEKLEELKETMEKSIEELRKEAE |
| HALC4_136 | MPNN | MSPYKKAIEITKRLLELLLSNPELAKKNLGGIATLISLLALISALDGTLDEKDIEPYIKKLEESL |
| HALC4_137 | MPNN | SHTHHVIISMLQELHDEVHSGHQEILDELRRLEEKVDEVLRVTKENLELLREMDSLLRELHEKLE |
| HALC4_138 | MPNN | ATRAIARAASKKEFDREDKRELVEATRKLGKLSEEDVPPVEEVDKEQFHLLTMALILSTVENLKK |
| HALC4_139 | MPNN | DLVPLIIAETALITGKAVMDFVYELTKSLTFDPDFSLEKLEELHEKSIKKLEDDLTLFAILALLL |
| HALC4_140 | MPNN | MEEVVLTSHNELHKKLDEVHDKIMSKLDEIHEKLDEIISKLDEIESKLHEILNIVKEIKEILEKK |
| HALC4_141 | MPNN | VDVREVVKINYEDIKDTPLVKKLGVEGFKARVDRLLDLVEKYIPDADEEDGRRLLLLILFRLEFG |
| HALC5_142 | AF2 | VGELLYESAKPFGSAQDGYRPQLIVKLTLEVIISLIVNNDINIPAATRIVKFIFNIEDELLQNPF |
| HALC5_143 | AF2 | LEYALRNPAVRELVTVDASILHYIRKTVRIGVLDRVYFTVIHPITAKPNKAGWIMISRLTPAVAE |
| HALC5_144 | AF2 | QAININLLIWRFQLVTLVKGTRYVQRVKEYIELAFRLLTTWVDPDSAQFMAMMLFIDALRTVIAA |
| HALC5_145 | AF2 | SGHAAAEISTYLEKLVQIVHQFSAPMVAYNIDIVNNLELVQSGIGSLADRVKKLNDTVSNKFTSL |
| HALC5_146 | AF2 | QLMKILKNDLDIREADLIRKAKAQLGGSHTVIGTIGFLLYIKRLQEYIVLIYTNIKVALFFHAEE |
| HALC5_147 | AF2 | IVKILQDLADVFPADLNRGKFKIVDLGVVIELIVNIEHRTNKASDLQDALDVFDLFLSAIADKYN |
| HALC5_148 | AF2 | TKMMAITLEVMLMGDLSQGWIYQRLYDQASESITRIRKDVPRHRMEAFAPQLTLKIVLWRSVRNE |
| HALC5_149 | AF2 | EPYAIDMIADINDDATAFSFSNAREVMALILTQLNLSDGTVRTIINSFFVLAYYENSGIGMDPPQ |
| HALC5_150 | AF2 | SDAVIVVNAVFFVLWLMIRVLSLALLFDEIEYFTAVTVIMFLPERDILRDPSFDLRDKTVHEAYQ |
| HALC5_151 | AF2 | TPTIKFVREFFRMQGKEMKKGNRDGFMQDHLAAYIGIAVIRSRRGDRIVNSLRDDYKHVAKLYFF |
| HALC5_152 | AF2 | GGELNKVLLALLNEFLNVLLPHYNQLIRLTREFLNEFVAMRTQLNIMVAKAFMLLKKLLSASLKA |
| HALC5_153 | AF2 | EALDPVLAAAISGFSFILYNLPNFLPAAHDHSRAAIETTIKLLNNWRIQNYYFVGNLGSLLYGPY |
| HALC5_154 | AF2 | DWRNLIKSYYEFEAFISATMVYYQNILNEVSNILKAVHTVLLANNAYLRSIEKQVSEINQGIGNL |
| HALC5_155 | AF2 | GDGHELYLLDKYSFIDKAVVLAAENITMANHGWGVNRVKDADRGYDLEPGASSLPPLTQALALDG |
| HALC5_156 | AF2 | EWLIVLSILAKFFNANQFATAADDFIPLYLNSRNPSVNRVTDEQFPKSLGSAIARLHALMIDKAN |
| HALC5_157 | AF2 | GIGEGFLKQSQNFSSRPDQVIKYRGQSLAALAIAERPNGQNTNLLLNIYFNEIVGLSREYPEREA |
| HALC5_158 | AF2 | VDATRLHEQAYTNIIELPQLLRQLTNEIMAHLTIRIELLLWVKRVVYDPLIPVIYILTDRMDDNR |
| HALC5_159 | AF2 | YESKTSYLYGLMKTIVYEAQMFGIDVLLDLYYYGIISIEIQSLLFDVEYYEFAMTVVTEVTKKRR |
| HALC5_160 | AF2 | PLRINLILATERRVFTLKLIKDLIDIAVRIEDPLLFIYITFGSLLKIGSIFEAVTAFEELMNAYY |
| HALC5_161 | AF2 | LKQQRSLWYDKVGHLLIFWEAYILLLWYYGERAPEIMNDLTVFEEDLVTDIYSEALQLGITLDDL |
| HALC5_162 | AF2 | RDVAVFMWEVFDITTRDRSIDPIFPSMVLSNYLALSLDVIYDSPGALEEEMNALKVFFFKANQQA |
| HALC5_163 | AF2 | VSEDDNKSFFFATLLHSEALALGILHLKGYALFGRSQGYPNPDLVETAQANNAKLKAMLAYISAF |
| HALC5_164 | MPNN | LSEKLKDPDTQTSVEVSYNIVEYIKETVPEEVLKNVLFTVEKPIKAKPDIEGWVKISNLTFEKAL |
| HALC5_165 | MPNN | LSEKLKNPETHSKVKVSANIIDYIKEEVPEEVLKKVKFTVRKPIQAKPDIKGWIEIENLTFEEAL |
| HALC5_166 | MPNN | LEEKLKNPRTWKKVTVSGNIIDYIKEKVSKEVLEKVLFTVTLPIQAKPDVSGYVTIENLTFEEAL |
| HALC5_167 | MPNN | SSLKEWLERWREKLVEAVKGTPEEEKVEKYLDLALESLEEMPDKKLAERIASRLFTEAVKTVVEA |
| HALC5_168 | MPNN | SELEDDLERFREELVARVRGTPHEERVEQYLDFALKLLKEMKDKEVAERIASRLFTEAIREVVEA |
| HALC5_169 | MPNN | LLLEVMEKVFDEEQLKLIKEAAEREGNSPVVISSIATLLLLERIEKIVKEIHDEVKKNNEKQEKK |
| HALC5_170 | MPNN | MVQILKQIDEVFKTRLNNGSFTVMDLGTVITILLHIKENSSDKEEVQEAEEILQTFLDAIKKKEE |
| HALC5_171 | MPNN | KPPLPIRLRIRIRADLRDPESLLRAYEEAEESLRRLEELLPPEVLERFLPHLEVEIELYREIRPE |
| HALC5_172 | MPNN | RPRPPIRLEVLIEADLSDPDSLLRAIEEAERTLERLERDLPPEVLERFRPHLRLEILLKKDIKPE |
| HALC5_173 | MPNN | EEWSKERLDALTKHREEYSYDNAMRVIELILKTEDLSEELKKKMIDVFYRLAYEKTAGVGVEPPE |
| HALC5_174 | MPNN | SSTVDFIKELFDKIEERLKKGDKSKSFESILSALIYIAAVKSDNWEEIVEELKPHFLKVLDLYSK |
| HALC5_175 | MPNN | PPGVTIEVESRLSLSEKAITLALTSIYLSSQGRTISEVRDSDTGKVLPPEDAKLPPVQQARLLSG |
| HALC5_176 | MPNN | MDPKELEREALKNIIKLPKLIQDFKDSVMKELNKIIELLEERRREIDEPLLPIIRKLQEELQKKE |
| HALC5_177 | MPNN | MKTKEERLRELTDKIVKLAKEKGFDVLVRLKEAGILNEEVQRLLYSPEYFDFTLEVVEKLTEELK |
| HALC5_178 | MPNN | KLTVNIKLYSKKKIFTKELIKEILDIAKETKDPNLEVEIYFETLLKIGTLEEALETLREILEYTK |
| HALC5_179 | MPNN | KLTVDLYVYSDRKVFTKELIKEIIDIAKETEDPDLKVTISFDTLLKIGSLEEAVETLKEILEYTK |
| HALC5_180 | MPNN | KVTLDLLIESRERVYTKELIKEIIDIAKKSKDPNLKITISFETLLKIGTVEEAIKTLEDLLNYNK |
| HALC5_181 | MPNN | SEEEFLRFSRLVTSLMVFIKTYLELRREEGERADEILKPYKSFYDRLLKEIKERAKKLGISEEEL |
| HALC5_182 | MPNN | NVLYKFVVFLFSEPHFRSRPEGVVLQSILIKVVRAFILYAAKEQMPLWYSFWSAAALVFHAGYYLRSGSVIRRGLDEIFDLAVSIEEESGQLETLRLIGLLSQVMLTALSIFDKSNEDER |
| HALC5_183 | MPNN | DIEKRIKDVRNSVPAKEEKHLGFRSDSDVFDIIWHQVHLDWINLTMNELDSASGADEAQRMAIYVALLDLVDYSRDIKTFAAIDEKLSDWMWNLYLAGKGSANQPRVLTMLVTASYALMRIVRGFSEFLKYHNIDWKGEL |
| HALC6_184 | AF2 | GDDAVAIRQRGLDFNGELQRFIGHIEEGMSIVDLFFIGHFSRAYQEVLLGVFLPSEEAGVFVDLL |
| HALC6_185 | AF2 | GGPEYKDPLYHFQRIREQLELILTETADYGQWVLGALDQPPNNLMTQQVIAKLIDAMIVNVLYYD |
| HALC6_186 | AF2 | KDRDYGGVHETVLVFLEHMNNNLSMESGGDKVIHLFAALDILLYQGWSFRLDLSGKKPLQESGRG |
| HALC6_187 | AF2 | PTVEAQKSLAMEVLKTWFVGDVFLGLLNYPHYVNVRSSDLEDAAMKLQNKVGVPEHFLYLRPNIS |
| HALC6_188 | AF2 | PDTPMAHNEAQKLPALVGSGVKLQLIIETMTNALNNPGLHDTDIKVYASQYIQLALVAAIITQGP |
| HALC6_189 | AF2 | DPAYTTDFKKFALRVSEQGLDTLTKTVDALERILVMLQQDLELFSYYHSLFLDMAVRVVSNAGGS |
| HALC6_190 | AF2 | LSKDLSFTLLWLLAAWEHSEDGKLAILMLEIGISGMNKAEYFINKNEPVSPKENEVIKAVFRVSS |
| HALC6_191 | AF2 | QQIPADDNHHYGLFVFFTIENNPDELGFLERLDLIQDMFRNGHWAVLSLRRLRDTKQKDLAVHRI |
| HALC6_192 | AF2 | FSADAVQDPEWLNTLALELYQDQTRDYVAGGAIAVALIYNANVLNRGIHALLFEMRNMIEIMHSV |
| HALC6_193 | AF2 | VFYLQTTVKAQLNLAVKYARVAKEQNAPVLVQHLLHVNSYIVGEKYRVGVDDESLKFMEDTILGR |
| HALC6_194 | AF2 | HTAQTFILSLMWIAVYVQANLYLVLATGMGQLMILVRLLGLELALRRTTEYIHKIYRDHADGKLR |
| HALC6_195 | AF2 | YKDWSFIKQFYARIEDYDIAYITSLFFSVSDPDYFAPLSILADKLTSIEPLKRSINIVYLIGNEV |
| HALC6_196 | AF2 | NSYEQVINLASDIIEVPQGLHARSVWFFELILWNVAKTVFKPLAADKIVDIAAILMAAHNLKFQT |
| HALC6_197 | AF2 | SIFPHYAEAMGMDPDNPAVLHLEHDVTSTLVDLRLQSLPHTKYDNTQELAHSLMDLVALIHDIRE |
| HALC6_198 | AF2 | IHLEEQPETVKTLEASDNPTDRLANWFINNVFTSWKKEAIITSINVLLGRMSIATPILPALNYLD |
| HALC6_199 | AF2 | NFDSVHLTFLSMLALKLMHYLEGQSILSFADNWLHAWDAMKELPADKRAEVASWTFRHLLTKVNI |
| HALC6_200 | AF2 | MGLDKKSGNPNASFDSELESKFRTLHTSYSKRAFALIFSILANTYGTPEFPFDTEAILEFIDLVT |
| HALC6_201 | AF2 | NEDRLRAVGYKKFALEYLVDFIDDTKDNRLSVEANSLILQLAQYLESFSSLWQFEVALPVILGLV |
| HALC6_202 | AF2 | WGTFVAIELLRRSIMRMFYHFLSYVKRIVHTLDSSLHIISKLANAVITRQKASLEFLAELERNIG |
| HALC6_203 | AF2 | PSKDLKAVHKYERIQEAVKGEEERESDARTHPSRILAGFVLIMANFLDFYTEHIVERRLDDNARG |
| HALC6_204 | MPNN | DTPEDRDPLYFFQKLLEKTREILESLGLWGKQILDALTQPPTDLLTQLVIKELLKALLRTVEKLL |
| HALC6_205 | MPNN | EPKDYSGILNTLKKILEWLNKNYTFDSNSGMWKILLDALDLLLRLGRDYDLDLTGKKPLKDAGVG |
| HALC6_206 | MPNN | MTVEQVKELSLELLRIQTISDIFLSLLTHPHTVDVEDETIRDLAKLLKDEIGIPERITDLRPKRK |
| HALC6_207 | MPNN | EEKEETRKHLQKIPELIASGFDIEKIVEQMTKDLNNEELSEEDKIKLADLYIRLALVGAVKEKGK |
| HALC6_208 | MPNN | RKIPYDPNRDLYITITLTVRNNPDQKSFLQSIDLLIKLLEQGYRVTINLVDFNTKEEKEQALQQL |
| HALC6_209 | MPNN | QKVPYDPNKDLKITITLTVRNRPDQLAFLDDIDKLQELLEQGYNVTINLVDLDTKEQKLEALNRL |
| HALC6_210 | MPNN | DEDLRESMKARLDLALELAKYAKEENREDLIRHLLDLLDELVSLKHRVGLDDDKLQFIKDTILGK |
| HALC6_211 | MPNN | LESDSFVEEFFDIIEKNNIRHIDEIYLNVRDPRQLEPLMKLVQRLKSIEPLRDRLRVVLFIGENF |
| HALC6_212 | MPNN | MKSDEFVEEFMKIIKEENIQHIDELHLQIRDPEELKPLLKLAQELKSIEPLKDRLRVVLYLGDTF |
| HALC6_213 | MPNN | MESEEFVEEFMKIVEELNIKHIDEIVLDIRDPRELGPLMKLVDYLKSIEPLKDKLKVVINIGSTV |
| HALC6_214 | MPNN | DVFSEFAKRMGLSPEDPLVLHIERSVFSTLVDLYLKSLPKEVLEDVEKLTEAIRRLLDLLRSIRE |
| HALC6_215 | MPNN | NVFEQFAKDMGLSPEDPLIQHIKHSVVTTLVRLYLESLPQETLKDVEKLTEAIRELLKLIKSLLE |
| HALC6_216 | MPNN | SFNNVLLKYLSDLSLHLMTKLTGEDIKTFARDFTEMLEELLELPEEERAREYTRRFLELLDRIDL |
| HALC6_217 | MPNN | SFEEVLLKFLSDATLHLSTHLTGEDRKSFLKKWTQMLLELRSLPEEERAERAAQQFLELLDEMDL |
| HALC6_218 | MPNN | KREEEELKKKKKEDLQKIVDYLKSHKSKKLSIEEKQLVLRIAEFISKLKTLEEYEIFRPVVELLL |
| HALC6_219 | MPNN | STNPSPSFSLNISPGFLDIVKLILDLYPNDKEVRERLINNLISQFKPSDNFPPTTVSLSISPSALELFKFVLKEFPNNEKVKDNLITNLINSLRFSTPKPPDTFVLNVSPEFIELLKFILEEFPDDEEVKQLLLDSIINLFGSS |
| HALC6_220 | MPNN | PPIPPPSFKLEISPAFLELVQLVIDLHPNDEEVRKELIENLISRIGKSDNVPPETISLDISEAALELFEWIFEKFPDDEDVHRRLIESFINKRKFSSSSPLDTPSLDISERFIELVKYILEKYPEDEEIKQKLIDSLLNLLGSY |
| HALC6_221 | MPNN | SLNPSSSFELNVSPGFLEVVNLVLELNPDDEEVRRRLLDNLISRIREGSNQPPDTISLSISPAALDLFKWILEKFPEDKKVKDRFIENLINRRNFSKPPPPDTFELNVSKSFIDLIRFILDLYPEDESIRQKLIDNIVNLLSSS |
| HALC6_222 | MPNN | NFSSSNLPLSTNNTLYLYLDFKLKLELNDDDLPVEPLAEALSNLNINFNFSSSNLPLSTNNTLYLYLDFKLKLELNDDPLPVEPLAEALSNLNINFNVSSSNLPLSTNNTLYLYLDFKLKLELNENPSSVEPLAEALSNLNINFNVSSSNLPLSTNNTLYLYLDFKLKLELNENDSSVEPLAEALSNLNFNLNVSSSNLPLSTNNTLYLYLDFKLKLELNDNDSSVEPLAEALSNLNFNL |
| HALC6_223 | MPNN | GRNSVGRLLLIITNQDVRRLFDPSILEETLRWAAHTLGPIRMGSAIELLEEITNNPVAQELLRGDTRHVEIVAVLSFSEEEEEAVLKESAVKLARLALRTAEVLEASGRPFRFTLVLVLTAHNYRFFAVVVVSNIIDISLTDPSIALLVRKLAEQLDVGTNVRPLVEILETILKDPLLQRFLQKKARTLQIHVVVAVSSEEEAAQAREAVKALLELAAEAAKLMRASGKPFVFNLILSLL |
| HALC6_224 | MPNN | GKNMVGELLLIVTNEDVKRLFNPEIIKETLEYAAFHEGPIRPEYAIKLLEELTNDPVVQKALRGNTRHVHIAVVLAFSREEEKEVLHEAAVRAVRLALRLAEVLAASGKPFRFTLVLLLTAHNYRFFAGVVVTNIIDYELLNPELYLEVRKLAEQLDVGTDVSPLVKILETILKDPLLQRFLTEYARTLQIFVVVAVSSEEEAEQARKAVKALIRLAVKAGELMRASGKPFRFVLILALL |
| HALC6_225 | MPNN | GRNSVYRFLLSVTNLPVRRLFDPSIIEEELRWAANHLGPLRPEYAIELLRRLTEDPVFQELLRGNTRHVHIFVALSYSYDDEKEVLTEAALEAARLALRTAKILEASGKPFHFTFVLLLTAHNKRFFVGLVVTNLIDFELLNPRISLLVRELALRLDVGTDVSPVVTILETILKDELFQRFLREHARTVQLHVHVAVSSDEEAEQAAEAVLALLKLAAEAAKLMAAGGKPFVFRLSLSLL |
| HALC6_226 | MPNN | GTVFTFIMLLIITNDDIFGASGSSMVEAIKAYSATMSGKANASFALEAVRALLANPKVQAFINTNTRFIYMEMFLTYNEDSQRKLVVEQALEFAELTLEAAERLAAKGEPFNVHMSFVLLSHTKRVQFLVVLTSADSVSYFGSSKVVDFAKELATVKGGIDVRPALELIERVAANERLQELLRTHVRTVVAVFVFQVSSESEFGYAIEATVKVAEQAIRVAEIMYNSGKPFGFTLQIFML |
| HALC6_227 | MPNN | TTFNLPSPSVENLFRGLARGLKVLKRIEHLELNLNFNLNLTNVNTFTLPDPSVENLFRGLAEGLKVLKKIEHLELNLNLNLNLTNVTTFTLPDPSVEELFRGLAEGLKVLKKIEHLTLNLNFNLNLTNVNTLKLPDPSLENLLRGLAEGLRVLKKIEHLELNLNFNLNLTNVNTFKLESPSLENLLRGLANGLRVLKKIEHLTLNLNFNLNLTNVNTFKLESPSVENLFRGLANGLKVLKRIEHLELNLNFNLNLTNV |
| HALC6_228 | MPNN | LNLDLVNPNLTELLQGLANGLKNLKNINNLNLELELTLTLSNVLNLDLVNPNLTELLQGLANGLKSLKNVNNLNLSLELTLTLSNVLNLDLVNPNDTEVLKGLANGLQSLKNVNNLTLELELTLTLSNVLNFDLVNPNDTEVLKGLAEGLQVLTHVNNLNLSLELTLTLTNVLNFSLTNPNPTLVLQGLAEGLKNLKHINNLNLELELTLTLSNVLNLSLTNPNPTLVLQALAEGLKNLKNINNLNLSLELTLTLSNV |
| HALC6_229 | MPNN | TSSPYIGIGLAEAFKAIGEGLKNLKYVEKLQLVITIPLDDIVPTSSPYIGIGLAEAFKAIGEGLKNLKYVEKLQLVITLPLDDIVPTSSPYIGIGLAEAFKAIGEGLQNLKYVEKLQLVITLPLDDIVPTSSPYIGIGLAEAFKAIGEGLKNLKYVEKLQLVITLPLDDIVPTSSPYIGIGLAEAFKAIGEGLKNLKYVEKLQLVITLPLDDIVPTSSPYIGIGLAEAFKAIGEGLKNLKYVEKLQLVITLPLDDIVPTSSPYIGIGLGEAFKAIGEGLKNLKYVEKLQLVITLPLDDIVP |
| HALC7_230 | AF2 | TEEIQVLWLLIDLFTKAEWGDKNGVLINQFTSEFGFKQQGRMTATKILFRTLYKTSRRYALRVEI |
| HALC7_231 | AF2 | SAKFLVDKVEKFVRAATKGIHIPAEDYVEAMVYALSPGHDRILLILLTVFQVAVRQFWRKARYME |
| HALC7_232 | AF2 | DHPFEGSLKMDVNDPHNEILVTMARSVWRSIHEGSGLNMLLAQTLSPILTFVFQTTVAALGFKDL |
| HALC7_233 | AF2 | SMERNILTLRALAPVLKKDYQDEFFIFTLDLQAAKDLTGLMEVVESMLNSSGSRAIVQFSNFEKE |
| HALC7_234 | AF2 | SRYQNVYNNDFGKKILKLVFQLVTYTEVRLEEKKDTTEPSETAIGMMVGAEGLLRALAYYFSLFD |
| HALC7_235 | AF2 | EHLQNAVDIGLKYLAKWASHKALNTPAMNFLASFANDFFIYIFHNVITRMGVEVALKALFALEAI |
| HALC7_236 | AF2 | QMPRFFIILETYKAGKTGREGYRKVTGDIMRILGTVSNWSNSRHLPLDVQVKHASYLPLDILQNV |
| HALC7_237 | AF2 | LLDMDLPKAYMIHAFLHIWVALARQDGEFARVILDVLLQLENTGEPISFENLMRAWKIYKELIGE |
| HALC7_238 | AF2 | EAYRDVLGYYRFALFVQIIAWLPEAMDRLKAYIDEVAVEEGADFSEQAEWRTAIAVTRVGYLKYM |
| HALC7_239 | AF2 | DEKDASTDPHVWNDAIEDLMIENDLTNLPANKYVEIFKTHFFHRGVVAAVVNVLFSHYLRVQREG |
| HALC7_240 | AF2 | EGKLVGLIAKMIENVEDIIKLLYVTHAIRDLVDDSNTPIGKFFLDYLILMIGGGKRQTKLKPLLD |
| HALC7_241 | AF2 | LTESLTNLVLGYVRKIAANKGTDILDKWVPLDWLTLIKSLNTEHIKIAIIILADTEMALAMDERV |
| HALC7_242 | AF2 | PYELLADLAIEVLVYYLRSEPRVFKPRVQDVAGAKPGQMFQLQNYFDSEHDAALKIFFTGAIDDQ |
| HALC7_243 | AF2 | SLFKALSTHLRNIAKNIYGVEFKPIAFNDPANLDAMELTVPSDNYYQAAYVNMEVLYLGLYGMQL |
| HALC7_244 | AF2 | VKLSVFSILKHFVTNRSPHLMQSATDIGSAYEKEDTPGAPMDFEDLLLAGFILIDALSLFLTMNN |
| HALC7_245 | AF2 | AALVLTLLADIGIKLGLSNYAVQLIMVAVPKDWRRMLSSLIPTGAQWQTIRQTGSWRTAESDLDG |
| HALC7_246 | AF2 | LMLFALLYIMAMGVVIQMFPILDRYIDFTTIQAIAPRLVAFVASDLYSALKKIDGYESESHFQDF |
| HALC7_247 | AF2 | VLSADLQSRLIKMNNIERIVPIMTLVYDVNFMLVHALGVPLAQEFGVAARPIQQIDEIIRAILPS |
| HALC7_248 | AF2 | GHVRYFALGIRPSMYISGAIIYTMDKNRWNNAAAFLDRVDFRFLEIVIPADLVNALNFEEVFGLN |
| HALC7_249 | AF2 | EAVVFGLVKFILKTLSTIISHRIFNPDSSWETLGYPAAKQAALQLVTMVNGEAKDVLAENLTSLE |
| HALC7_250 | AF2 | NSQNVLAWLNAVNKYSVDRLQILAPILLPLGAKLVWSLIESPFFDEVKVQHRVLSLITKFVRRLL |
| HALC7_251 | AF2 | VDLVKRKFLEVMYGALYIPGVGPVASFASVLILLSLTFRSRGVHEWLGLIFEDPTPSDVIYGKSQ |
| HALC7_252 | AF2 | GEIGPGGFATLALTMLRPIYEPEEAGYDEIISFVDMKLGIVIRLLEVGSLNPTYVEVAHLMLRDG |
| HALC7_253 | MPNN | SRETIESYIRTIIDAAVNGEYVPAETFTDALVLSLEPGNEKALRILLTAFEVAVRNYWEVKKEKK |
| HALC7_254 | MPNN | TQKENVEQLRALEPVLRGPYSDHKFVFTLDLSDYKDKEALKEVVEAALRGAGSEVTIVFTNFLPD |
| HALC7_255 | MPNN | TTEENVEQLEFLKPVLKSNYSHHTFVFTLNLSDYKDKEGLKKVVQAALDGAGSKVTITFTNFNPD |
| HALC7_256 | MPNN | SEERELVRLTLQQVKEVQNRPDLDTPSNRFFSDFFRDFSLTILRLVFEKTGPRLALEALQLANEL |
| HALC7_257 | MPNN | SREKRRNDPDVRNEGIYKLLVEHDLENMPDEKFVEIISENFHNDLEHAEIFKVITEHWLRQKEQQ |
| HALC7_258 | MPNN | SEERRKLVDRMLEKVDEIVRLLRVHQAVQELRDSSHTPLGRFYLDYLIELVAGEEGREVLRPLLD |
| HALC7_259 | MPNN | DRELLYELLKLTIDTIGRVYGEETKDKYIPPEFEELAERLNVEQLKWALKFVNTLFLALALEEKK |
| HALC12-6_260 | MPNN | SIPREEQLRLLTLTFARYADPESLKILLTVLEQNFKIALHIPDPAERQRRLDFIAEVATRGLFAWNVMKNVSHEDYQAAVRELQRPNNGTLTPEQARMLDRRYMPPKYELVRLYLESQNPSIPREEQLRLLTLTFARYADPESLKILLTVLEQNFKIALHIPDPAERQRRLDFIAEVATRGLFAWNVMKNVSHEDYQAAVRELQRPNNGTLTPEQARMLDRRYMPPKYELVRLYLESQNP |
| HALC12-6_261 | MPNN | SIPREEQLRLLTLAVYRYADPEEIELLLRVLERNFEIALHIPDEKERARRLEFLAEVATRGLVAWSLMKRVSHEDFQAALVEMARPNNGVLTPEQARMRDPRHMPPKYDLVRLYVQAQDPSIPREEQLRLLTLAVYRYADPEEIELLLRVLERNFEIALHIPDEKERARRLEFLAEVATRGLVAWSLMKRVSHEDFQAALVEMARPNNGVLTPEQARMRDPRHMPPKYDLVRLYVQAQDP |
| HALC15-5_262 | MPNN | DVPLTDPKNLNEFLYALGEGLKGMKNLKKLTLTFPSNPLTIPGDISEGFRELGEGLKGMKNLEELTVTFNDVPLTDPKNLNEFLYALGEGLKGMKNLKKLTLTFPSNPLTIPGDISEGFRELGEGLKGMKNLEELTVTFNDVPLTDPKNLNEFLYALGEGLKGMKNLKKLTLTFPSNPLTIPGDISEGFRELGEGLKGMKNLEELTVTFN |
| HALC16-4_263 | MPNN | IVTFQLSFSLSLSYEKYNPFGVNDPNLSRAAREFAKGFENAAKELSKLPGSIVTFQLSFSLSLSYEKYNPFGVNDPNLSRAAREFAKGFENAAKELSKLPGSIVTFQLSFSLSLSYEKYNPFGVNDPNLSRAAREFAKGFENAAKELSKLPGSIVTFQLSFSLSLSYEKYNPFGVNDPNLSRAAREFAKGFENAAKELSKLPGS |
| HALC18-6_264 | MPNN | NSKPPDTFRLEISQASLALIYTIMLWFPADQRVRDDLVKALIGMLYYANSKPPDTFRLEISQASLALIYTIMLWFPADQRVRDDLVKALIGMLYYANSKPPDTFRLEISQASLALIYTIMLWFPADQRVRDDLVKALIGMLYYA |
| HALC18-6_265 | MPNN | NIKIPNPKDLSELLKKLGEGLKGLPNLKTLTLTLSNIELPEDADLSPGAEGLGEGLKGLPNLETLTFTISNIKIPNPKDLSELLKKLGEGLKGLPNLKTLTLTLSNIELPEDADLSPGAEGLGEGLKGLPNLETLTFTISNIKIPNPKDLSELLKKLGEGLKGLPNLKTLTLTLSNIELPEDADLSPGAEGLGEGLKGLPNLETLTFTIS |
| HALC18-6_266 | MPNN | GSEILNPKSLDYLLYHIGEGLRGLPNLEELDLTLYSGKLPPDSDLSKGAKGLGEGLRGLPNLRRLTITISGSEILNPKSLDYLLYHIGEGLRGLPNLEELDLTLYSGKLPPDSDLSKGAKGLGEGLRGLPNLRRLTITISGSEILNPKSLDYLLYHIGEGLRGLPNLEELDLTLYSGKLPPDSDLSKGAKGLGEGLRGLPNLRRLTITIS |
| HALC18-6_267 | MPNN | DVPLTDPKNLNEFLYALGEGLKGMKNLKKLTLTFPSNPLTIPGDISEGFRELGEGLKGMKNLEELTVTFNDVPLTDPKNLNEFLYALGEGLKGMKNLKKLTLTFPSNPLTIPGDISEGFRELGEGLKGMKNLEELTVTFNDVPLTDPKNLNEFLYALGEGLKGMKNLKKLTLTFPSNPLTIPGDISEGFRELGEGLKGMKNLEELTVTFN |
| HALC18-6_268 | MPNN | NNNISDPRVLAELLRAIGRGLRGLKNLKELNITLPSLNITDSSRLKPGFEELGRGLRGLENLEKLNITFNNNNISDPRVLAELLRAIGRGLRGLKNLKELNITLPSLNITDSSRLKPGFEELGRGLRGLENLEKLNITFNNNNISDPRVLAELLRAIGRGLRGLKNLKELNITLPSLNITDSSRLKPGFEELGRGLRGLENLEKLNITFN |
| HALC18-6_269 | MPNN | GNYISDIRVPAELIRKLGDGLKSLKYLQQLNLSLPLIIGQDPSEMQEGFDQLGDGLKSLQYLQQLNITFNGNYISDIRVPAELIRKLGDGLKSLKYLQQLNLSLPLIIGQDPSEMQEGFDQLGDGLKSLQYLQQLNITFNGNYISDIRVPAELIRKLGDGLKSLKYLQQLNLSLPLIIGQDPSEMQEGFDQLGDGLKSLQYLQQLNITFN |
| HALC18-6_270 | MPNN | VKLLEDPRVPAFLLYSLGKGLQSPVPITKLKLTLPGVKTEKDPLIEKAFKELGKGLSRLPSPLDLTLTFNVKLLEDPRVPAFLLYSLGKGLQSPVPITKLKLTLPGVKTEKDPLIEKAFKELGKGLSRLPSPLDLTLTFNVKLLEDPRVPAFLLYSLGKGLQSPVPITKLKLTLPGVKTEKDPLIEKAFKELGKGLSRLPSPLDLTLTFN |
| HALC18-6_271 | MPNN | VDILKDPRVPALLLKALGQGLQSPVPITELELTLPGFVEEEDPLLEEAFKELGQGLQQLPSPLKLRLTFRVDILKDPRVPALLLKALGQGLQSPVPITELELTLPGFVEEEDPLLEEAFKELGQGLQQLPSPLKLRLTFRVDILKDPRVPALLLKALGQGLQSPVPITELELTLPGFVEEEDPLLEEAFKELGQGLQQLPSPLKLRLTFR |
| HALC18-6_272 | MPNN | VDYLPDPRLPILLLRYLGKGLTSPNKITELELNLPGFTSPDEKEWEEAFKELGKGLSKLPYPLKLKLSFNVDYLPDPRLPILLLRYLGKGLTSPNKITELELNLPGFTSPDEKEWEEAFKELGKGLSKLPYPLKLKLSFNVDYLPDPRLPILLLRYLGKGLTSPNKITELELNLPGFTSPDEKEWEEAFKELGKGLSKLPYPLKLKLSFN |
| HALC18-6_273 | MPNN | VEYLPNPSFITELLYYLGYGLTSPNPITRLTLDLPGFTSPDFESIREAFEKLGYGLSKLKHPLDLTLRFRVEYLPNPSFITELLYYLGYGLTSPNPITRLTLDLPGFTSPDFESIREAFEKLGYGLSKLKHPLDLTLRFRVEYLPNPSFITELLYYLGYGLTSPNPITRLTLDLPGFTSPDFESIREAFEKLGYGLSKLKHPLDLTLRFR |
| HALC18-6_274 | MPNN | GEETTNPLYIGQLLKSLGKGLEKLEKLTTLTLTLPVFTTSVSPILEEGFKELGKGLSKIKEPLSLTITVLGEETTNPLYIGQLLKSLGKGLEKLEKLTTLTLTLPVFTTSVSPILEEGFKELGKGLSKIKEPLSLTITVLGEETTNPLYIGQLLKSLGKGLEKLEKLTTLTLTLPVFTTSVSPILEEGFKELGKGLSKIKEPLSLTITVL |
| HALC18-6_275 | MPNN | GSQTNNPYYIGTLLLALGKGLEKNKNLKNLTLNLPTFSSTYSPLFEQGFKELGKGLSKIEQPLNLSISVLGSQTNNPYYIGTLLLALGKGLEKNKNLKNLTLNLPTFSSTYSPLFEQGFKELGKGLSKIEQPLNLSISVLGSQTNNPYYIGTLLLALGKGLEKNKNLKNLTLNLPTFSSTYSPLFEQGFKELGKGLSKIEQPLNLSISVL |
| HALC18-6_276 | MPNN | GQYTPNPRYPGLLLYYLGEGLKKKKKLKRLRLSLPVFTTDGSPLLEEGFRSLGEGLSKIEEPLDLDLTFLGQYTPNPRYPGLLLYYLGEGLKKKKKLKRLRLSLPVFTTDGSPLLEEGFRSLGEGLSKIEEPLDLDLTFLGQYTPNPRYPGLLLYYLGEGLKKKKKLKRLRLSLPVFTTDGSPLLEEGFRSLGEGLSKIEEPLDLDLTFL |
| HALC18-6_277 | MPNN | GILTENPYYVGVLLLHLGQGLRRRSRLRRLTLSLPVFSSETNPLLERGFQALGQGLSRLEEPLDLTLTFNGILTENPYYVGVLLLHLGQGLRRRSRLRRLTLSLPVFSSETNPLLERGFQALGQGLSRLEEPLDLTLTFNGILTENPYYVGVLLLHLGQGLRRRSRLRRLTLSLPVFSSETNPLLERGFQALGQGLSRLEEPLDLTLTFN |
| HALC18-6_278 | MPNN | GERTDNPYYIGLLLKHLGEGLKKNKKLEKLKLDLPVFTTEPNPILEEGFKLLGEGLANIESPLDLEIKILGERTDNPYYIGLLLKHLGEGLKKNKKLEKLKLDLPVFTTEPNPILEEGFKLLGEGLANIESPLDLEIKILGERTDNPYYIGLLLKHLGEGLKKNKKLEKLKLDLPVFTTEPNPILEEGFKLLGEGLANIESPLDLEIKIL |
| HALC18-6_279 | MPNN | GDRTDNPREIGTLLRYLGQGLQKLKNLTSLTLTLPVFSSPPDPVLEQGFQELGQGLANIETPLTLTLTFKGDRTDNPREIGTLLRYLGQGLQKLKNLTSLTLTLPVFSSPPDPVLEQGFQELGQGLANIETPLTLTLTFKGDRTDNPREIGTLLRYLGQGLQKLKNLTSLTLTLPVFSSPPDPVLEQGFQELGQGLANIETPLTLTLTFK |
| HALC18-6_280 | MPNN | GTRTDNPTHLGLLLKHLGEGLSNQTNLSKLELDLPVFDSELNPVLEEGFEKLGEGIANVKSPLELTITFLGTRTDNPTHLGLLLKHLGEGLSNQTNLSKLELDLPVFDSELNPVLEEGFEKLGEGIANVKSPLELTITFLGTRTDNPTHLGLLLKHLGEGLSNQTNLSKLELDLPVFDSELNPVLEEGFEKLGEGIANVKSPLELTITFL |
| HALC18-6_281 | MPNN | GEETDNPTTLGLLLKELGKGLKENKNLVRLSLSLPVFTSPVSPLLVEGFKELGKGLKNVKSPLQLSLTFLGEETDNPTTLGLLLKELGKGLKENKNLVRLSLSLPVFTSPVSPLLVEGFKELGKGLKNVKSPLQLSLTFLGEETDNPTTLGLLLKELGKGLKENKNLVRLSLSLPVFTSPVSPLLVEGFKELGKGLKNVKSPLQLSLTFL |
| HALC18-6_282 | MPNN | GDRTSNPRYIGLLLKHLGEGLKNLKNLTSLNLTLPVFDTDLDPIFEEGFEELGEGLKNVETPLNLNLTFLGDRTSNPRYIGLLLKHLGEGLKNLKNLTSLNLTLPVFDTDLDPIFEEGFEELGEGLKNVETPLNLNLTFLGDRTSNPRYIGLLLKHLGEGLKNLKNLTSLNLTLPVFDTDLDPIFEEGFEELGEGLKNVETPLNLNLTFL |
| HALC18-6_283 | MPNN | GDFTDNPRYIGTLLKYLGEGLKKHENLSELRLDLPVFTSEPDPTVEEGFNSLGEGLANVKSPLSLTLTFKGDFTDNPRYIGTLLKYLGEGLKKHENLSELRLDLPVFTSEPDPTVEEGFNSLGEGLANVKSPLSLTLTFKGDFTDNPRYIGTLLKYLGEGLKKHENLSELRLDLPVFTSEPDPTVEEGFNSLGEGLANVKSPLSLTLTFK |
| HALC18-6_284 | MPNN | GEKTNNPEYIGLLLKHLGEGLKKNKNLINLNLTLPVFTSERSPLLEEGFEALGEGIANIKSPLNLTLTFLGEKTNNPEYIGLLLKHLGEGLKKNKNLINLNLTLPVFTSERSPLLEEGFEALGEGIANIKSPLNLTLTFLGEKTNNPEYIGLLLKHLGEGLKKNKNLINLNLTLPVFTSERSPLLEEGFEALGEGIANIKSPLNLTLTFL |
| HALC18-6_285 | MPNN | NVQVPSNVDPTQTWKWIGDGLSGLPNLKKLNLTFSNVQFPSNWDLGQTWKWIGDGLSGLPNLQELNLSFSNVQVPSNVDPTQTWKWIGDGLSGLPNLKKLNLTFSNVQFPSNWDLGQTWKWIGDGLSGLPNLQELNLSFSNVQVPSNVDPTQTWKWIGDGLSGLPNLKKLNLTFSNVQFPSNWDLGQTWKWIGDGLSGLPNLQELNLSFS |
| HALC18-6_286 | MPNN | GDFTSNPYFIGLLLYHLGKGLKQLKNLSNLNLDLPLFTTPPNPLLEEGFKELGKGLKNVKTPLNLNLTIRGDFTSNPYFIGLLLYHLGKGLKQLKNLSNLNLDLPLFTTPPNPLLEEGFKELGKGLKNVKTPLNLNLTIRGDFTSNPYFIGLLLYHLGKGLKQLKNLSNLNLDLPLFTTPPNPLLEEGFKELGKGLKNVKTPLNLNLTIR |
| HALC18-6_287 | MPNN | GERTENPLYLGRLLYWLGKGLKELRNLTSLTLELPLFTSPESPVLEEGFKELGKGLKEIKSPLTLTLKFLGERTENPLYLGRLLYWLGKGLKELRNLTSLTLELPLFTSPESPVLEEGFKELGKGLKEIKSPLTLTLKFLGERTENPLYLGRLLYWLGKGLKELRNLTSLTLELPLFTSPESPVLEEGFKELGKGLKEIKSPLTLTLKFL |
| HALC18-6_288 | MPNN | NITLPEGVDLSKVFREIGRGLQGLPNLKKLNLTISGITLPEPLDLSKGFEGIGKALQKVKNLEELNFTISNITLPEGVDLSKVFREIGRGLQGLPNLKKLNLTISGITLPEPLDLSKGFEGIGKALQKVKNLEELNFTISNITLPEGVDLSKVFREIGRGLQGLPNLKKLNLTISGITLPEPLDLSKGFEGIGKALQKVKNLEELNFTIS |
| HALC18-6_289 | MPNN | NLKIPNGFDISKVLYHIGKGLEGLNNLKTLHLTLSGLKINTPYDISKGFEGLGKALEKVKNLEELHFTLSNLKIPNGFDISKVLYHIGKGLEGLNNLKTLHLTLSGLKINTPYDISKGFEGLGKALEKVKNLEELHFTLSNLKIPNGFDISKVLYHIGKGLEGLNNLKTLHLTLSGLKINTPYDISKGFEGLGKALEKVKNLEELHFTLS |
| HALC18-6_290 | MPNN | NLTLPDGVDLSKVFYWLGEGLQGLDNLKRLNLTITGLTLPEPIDLSKGFKGLGEGLQKSKQLDELNFTISNLTLPDGVDLSKVFYWLGEGLQGLDNLKRLNLTITGLTLPEPIDLSKGFKGLGEGLQKSKQLDELNFTISNLTLPDGVDLSKVFYWLGEGLQGLDNLKRLNLTITGLTLPEPIDLSKGFKGLGEGLQKSKQLDELNFTIS |
| HALC18-6_291 | MPNN | NLNLPDGFDLDKVFYELGEGLKGLSNLKHLNLTISGLNLPEPVDISKGFKGLGEALQQAKNLESLNFTLSNLNLPDGFDLDKVFYELGEGLKGLSNLKHLNLTISGLNLPEPVDISKGFKGLGEALQQAKNLESLNFTLSNLNLPDGFDLDKVFYELGEGLKGLSNLKHLNLTISGLNLPEPVDISKGFKGLGEALQQAKNLESLNFTLS |
| HALC18-6_292 | MPNN | NINLPNGVDLSSVLLAIGQGLKGLKNLKKLNLTISGLNLPEPLDISSGFLGLGQALKETEKLEELNFTISNINLPNGVDLSSVLLAIGQGLKGLKNLKKLNLTISGLNLPEPLDISSGFLGLGQALKETEKLEELNFTISNINLPNGVDLSSVLLAIGQGLKGLKNLKKLNLTISGLNLPEPLDISSGFLGLGQALKETEKLEELNFTIS |
| HALC18-6_293 | MPNN | NTKLPDSPDLKGLYDAFGEGLRGLPNLEKLNLILKNVELPPSVDLSEAFEGLGEGLRGLPNLKELNLILKNTKLPDSPDLKGLYDAFGEGLRGLPNLEKLNLILKNVELPPSVDLSEAFEGLGEGLRGLPNLKELNLILKNTKLPDSPDLKGLYDAFGEGLRGLPNLEKLNLILKNVELPPSVDLSEAFEGLGEGLRGLPNLKELNLILK |
| HALC18-6_294 | MPNN | DTTLPNSPNISGIFSGLGDGLRGLPNLEYLELTLSNVQFHPNLDLTPGFDALGRGLRSLPNLKYLKFSLSDTTLPNSPNISGIFSGLGDGLRGLPNLEYLELTLSNVQFHPNLDLTPGFDALGRGLRSLPNLKYLKFSLSDTTLPNSPNISGIFSGLGDGLRGLPNLEYLELTLSNVQFHPNLDLTPGFDALGRGLRSLPNLKYLKFSLS |
| HALC18-6_295 | MPNN | DVRLPGHQLEAFVKFLFGVGLSGLPSLEELSFSVRDLRVPDDVDLTEAFEAVGKGLSSLPSLRRLSFSVRDVRLPGHQLEAFVKFLFGVGLSGLPSLEELSFSVRDLRVPDDVDLTEAFEAVGKGLSSLPSLRRLSFSVRDVRLPGHQLEAFVKFLFGVGLSGLPSLEELSFSVRDLRVPDDVDLTEAFEAVGKGLSSLPSLRRLSFSVR |
| HALC18-6_296 | MPNN | DVNIPNGEQLNQFILELGEGLRGLPNLKKLTLSLKNVNIPPDADLTEGYRGLGEGLRNLPNLEELNFSLKDVNIPNGEQLNQFILELGEGLRGLPNLKKLTLSLKNVNIPPDADLTEGYRGLGEGLRNLPNLEELNFSLKDVNIPNGEQLNQFILELGEGLRGLPNLKKLTLSLKNVNIPPDADLTEGYRGLGEGLRNLPNLEELNFSLK |
| HALC18-6_297 | MPNN | NVNIPNGEILNEYFRELGDGLRGLPNLKELNLTLTNVNIPPDADLREGFKALGDGLRGLPNLEKLNFTLTNVNIPNGEILNEYFRELGDGLRGLPNLKELNLTLTNVNIPPDADLREGFKALGDGLRGLPNLEKLNFTLTNVNIPNGEILNEYFRELGDGLRGLPNLKELNLTLTNVNIPPDADLREGFKALGDGLRGLPNLEKLNFTLT |
| HALC18-6_298 | MPNN | NIPWPRGEDLSYELLKLGDGLSGLKNLEHLTLTLENIPLPPDADLRPGFEGLGDGLSGLKNLKHLDFTIENIPWPRGEDLSYELLKLGDGLSGLKNLEHLTLTLENIPLPPDADLRPGFEGLGDGLSGLKNLKHLDFTIENIPWPRGEDLSYELLKLGDGLSGLKNLEHLTLTLENIPLPPDADLRPGFEGLGDGLSGLKNLKHLDFTIE |
| HALC20-4_299 | MPNN | ILSYSLSFSLSLSWTKYSPFGVTDPSLERAARAFAQGITQAAYFLSQLPGSILSYSLSFSLSLSWTKYSPFGVTDPSLERAARAFAQGITQAAYFLSQLPGSILSYSLSFSLSLSWTKYSPFGVTDPSLERAARAFAQGITQAAYFLSQLPGSILSYSLSFSLSLSWTKYSPFGVTDPSLERAARAFAQGITQAAYFLSQLPGSILSYSLSFSLSLSWTKYSPFGVTDPSLERAARAFAQGITQAAYFLSQLPGS |
| HALC20-4_300 | MPNN | AAAVELKLELELSLYPSGGIKLDPESLVEMAKSLADAMFLSAMTLALALGTSVTSAAAVELKLELELSLYPSGGIKLDPESLVEMAKSLADAMFLSAMTLALALGTSVTSAAAVELKLELELSLYPSGGIKLDPESLVEMAKSLADAMFLSAMTLALALGTSVTSAAAVELKLELELSLYPSGGIKLDPESLVEMAKSLADAMFLSAMTLALALGTSVTSAAAVELKLELELSLYPSGGIKLDPESLVEMAKSLADAMFLSAMTLALALGTSVTS |
| HALC20-5_301 | AF2 | LDKLDRLYLHLPTFMIGLRPRPEHDMTRAARALAHGFERAAHSFADLPGSLDKLDRLYLHLPTFMIGLRPRPEHDMTRAARALAHGFERAAHSFADLPGSLDKLDRLYLHLPTFMIGLRPRPEHDMTRAARALAHGFERAAHSFADLPGSLDKLDRLYLHLPTFMIGLRPRPEHDMTRAARALAHGFERAAHSFADLPGS |
| HALC20-5_302 | MPNN | PLDKLKELNLTFPDFEVELYPPDSHDMVKATKYLADGYTNAVKFLSKLPGPLDKLKELNLTFPDFEVELYPPDSHDMVKATKYLADGYTNAVKFLSKLPGPLDKLKELNLTFPDFEVELYPPDSHDMVKATKYLADGYTNAVKFLSKLPGPLDKLKELNLTFPDFEVELYPPDSHDMVKATKYLADGYTNAVKFLSKLPG |
| HALC20-5_303 | MPNN | PLSSLERLDLTFPNVEINLYPPSSHNMVEATRLFAEGYTEAAKELAKLPGPLSSLERLDLTFPNVEINLYPPSSHNMVEATRLFAEGYTEAAKELAKLPGPLSSLERLDLTFPNVEINLYPPSSHNMVEATRLFAEGYTEAAKELAKLPGPLSSLERLDLTFPNVEINLYPPSSHNMVEATRLFAEGYTEAAKELAKLPG |
| HALC20-5_304 | MPNN | PLSSLRELDLTFPNFYVELYPPDSHDMVKATKYLAEGYRKAVEALSKLPGPLSSLRELDLTFPNFYVELYPPDSHDMVKATKYLAEGYRKAVEALSKLPGPLSSLRELDLTFPNFYVELYPPDSHDMVKATKYLAEGYRKAVEALSKLPGPLSSLRELDLTFPNFYVELYPPDSHDMVKATKYLAEGYRKAVEALSKLPG |
| HALC20-5_305 | AF2 | LQRIKFVWIIDLTRTYYHPDGTRDKDMTRAARALAHGFERAAHSFAQLPGSSLQRIKFVWIIDLTRTYYHPDGTRDKDMTRAARALAHGFERAAHSFAQLPGSSLQRIKFVWIIDLTRTYYHPDGTRDKDMTRAARALAHGFERAAHSFAQLPGSSLQRIKFVWIIDLTRTYYHPDGTRDKDMTRAARALAHGFERAAHSFAQLPGSS |
| HALC20-5_306 | AF2 | VVYTELTFHVILYPSAKYASPETAQDMTRAARALAHGFERAAHSFAENLPGSSVVYTELTFHVILYPSAKYASPETAQDMTRAARALAHGFERAAHSFAENLPGSSVVYTELTFHVILYPSAKYASPETAQDMTRAARALAHGFERAAHSFAENLPGSSVVYTELTFHVILYPSAKYASPETAQDMTRAARALAHGFERAAHSFAENLPGSS |
| HALC20-5_307 | AF2 | ALYTELTFHVFLYPSARETPPSFSQDMTRAARALAHGFERAAHSFAENLGSSGALYTELTFHVFLYPSARETPPSFSQDMTRAARALAHGFERAAHSFAENLGSSGALYTELTFHVFLYPSARETPPSFSQDMTRAARALAHGFERAAHSFAENLGSSGALYTELTFHVFLYPSARETPPSFSQDMTRAARALAHGFERAAHSFAENLGSSG |
| HALC20-5_308 | MPNN | SLGWKVLLHLDLTWYPGADYTIDHDDMTRAARALAHGFERAAHSFAEAIGSTGSLGWKVLLHLDLTWYPGADYTIDHDDMTRAARALAHGFERAAHSFAEAIGSTGSLGWKVLLHLDLTWYPGADYTIDHDDMTRAARALAHGFERAAHSFAEAIGSTGSLGWKVLLHLDLTWYPGADYTIDHDDMTRAARALAHGFERAAHSFAEAIGSTG |
| HALC20-5_309 | MPNN | AAAWNLELHLHLSLNPSSNINLDPPFLVLMALALALAMFLGFLSFALALGTSVTSAAAWNLELHLHLSLNPSSNINLDPPFLVLMALALALAMFLGFLSFALALGTSVTSAAAWNLELHLHLSLNPSSNINLDPPFLVLMALALALAMFLGFLSFALALGTSVTSAAAWNLELHLHLSLNPSSNINLDPPFLVLMALALALAMFLGFLSFALALGTSVTS |
| HALC24-4_310 | MPNN | VEKLGISRETLEGIVNKLLDQNDPEILKLALEVLKLGTSVEKLGISRETLEGIVNKLLDQNDPEILKLALEVLKLGTSVEKLGISRETLEGIVNKLLDQNDPEILKLALEVLKLGTSVEKLGISRETLEGIVNKLLDQNDPEILKLALEVLKLGTSVEKLGISRETLEGIVNKLLDQNDPEILKLALEVLKLGTSVEKLGISRETLEGIVNKLLDQNDPEILKLALEVLKLGTS |
| HALC24-4_311 | MPNN | VIEPLAKLLSTIVEKSNDELKKYIIEKVFPYLVELIKKKSGTSTSVIEPLAKLLSTIVEKSNDELKKYIIEKVFPYLVELIKKKSGTSTSVIEPLAKLLSTIVEKSNDELKKYIIEKVFPYLVELIKKKSGTSTSVIEPLAKLLSTIVEKSNDELKKYIIEKVFPYLVELIKKKSGTSTSVIEPLAKLLSTIVEKSNDELKKYIIEKVFPYLVELIKKKSGTSTSVIEPLAKLLSTIVEKSNDELKKYIIEKVFPYLVELIKKKSGTSTS |
| HALC24-4_312 | MPNN | VPPSLAELLSEIIKVSDEKLKEKILKEVYPDLFELLKKKSGTSTSVPPSLAELLSEIIKVSDEKLKEKILKEVYPDLFELLKKKSGTSTSVPPSLAELLSEIIKVSDEKLKEKILKEVYPDLFELLKKKSGTSTSVPPSLAELLSEIIKVSDEKLKEKILKEVYPDLFELLKKKSGTSTSVPPSLAELLSEIIKVSDEKLKEKILKEVYPDLFELLKKKSGTSTSVPPSLAELLSEIIKVSDEKLKEKILKEVYPDLFELLKKKSGTSTS |
| HALC24-4_313 | MPNN | MKKLLEAGISRELLLEIIREILKLNNPEVLKLMLELLLLGSTSGTSMKKLLEAGISRELLLEIIREILKLNNPEVLKLMLELLLLGSTSGTSMKKLLEAGISRELLLEIIREILKLNNPEVLKLMLELLLLGSTSGTSMKKLLEAGISRELLLEIIREILKLNNPEVLKLMLELLLLGSTSGTSMKKLLEAGISRELLLEIIREILKLNNPEVLKLMLELLLLGSTSGTSMKKLLEAGISRELLLEIIREILKLNNPEVLKLMLELLLLGSTSGTS |
| HALC24-4_314 | MPNN | EALKELAKALEEFAKELGLALSKLKNLKELNLDLKLDLKSGSTQTGSEALKELAKALEEFAKELGLALSKLKNLKELNLDLKLDLKSGSTQTGSEALKELAKALEEFAKELGLALSKLKNLKELNLDLKLDLKSGSTQTGSEALKELAKALEEFAKELGLALSKLKNLKELNLDLKLDLKSGSTQTGSEALKELAKALEEFAKELGLALSKLKNLKELNLDLKLDLKSGSTQTGSEALKELAKALEEFAKELGLALSKLKNLKELNLDLKLDLKSGSTQTGS |
| HALC24-6_315 | MPNN | VEFLGISRETLEGIVEKLLNLNDPEYLKLLLEVLKLGTSVEFLGISRETLEGIVEKLLNLNDPEYLKLLLEVLKLGTSVEFLGISRETLEGIVEKLLNLNDPEYLKLLLEVLKLGTSVEFLGISRETLEGIVEKLLNLNDPEYLKLLLEVLKLGTS |
| HALC24-6_316 | AF2 | SKEKLGIQQDLFEGIIATLLSHKDPRVLYLMVTILKLTGSSPSKEKLGIQQDLFEGIIATLLSHKDPRVLYLMVTILKLTGSSPSKEKLGIQQDLFEGIIATLLSHKDPRVLYLMVTILKLTGSSPSKEKLGIQQDLFEGIIATLLSHKDPRVLYLMVTILKLTGSSP |
| HALC24-6_317 | AF2 | RALEKWADAERTMYRTIGEALGRHQDLRVLHLSFHNDLYGSSPRALEKWADAERTMYRTIGEALGRHQDLRVLHLSFHNDLYGSSPRALEKWADAERTMYRTIGEALGRHQDLRVLHLSFHNDLYGSSPRALEKWADAERTMYRTIGEALGRHQDLRVLHLSFHNDLYGSSP |
| HALC24-6_318 | MPNN | VPKSLANLLSEIIKVSDEKLKTYILENVYPDLFELLRKKSGTSTSVPKSLANLLSEIIKVSDEKLKTYILENVYPDLFELLRKKSGTSTSVPKSLANLLSEIIKVSDEKLKTYILENVYPDLFELLRKKSGTSTSVPKSLANLLSEIIKVSDEKLKTYILENVYPDLFELLRKKSGTSTS |
| HALC24-6_319 | MPNN | VALEELAKAFKEFAEKLGEALKELKNLKELHIDITLLLKSGSTQTGSVALEELAKAFKEFAEKLGEALKELKNLKELHIDITLLLKSGSTQTGSVALEELAKAFKEFAEKLGEALKELKNLKELHIDITLLLKSGSTQTGSVALEELAKAFKEFAEKLGEALKELKNLKELHIDITLLLKSGSTQTGS |
| HALC24-6_320 | MPNN | PSLLPLLLYFFDVYRGFAEALSSLKNLQKLTLVSPYGVGYALSEFFRNLPNDPSLLPLLLYFFDVYRGFAEALSSLKNLQKLTLVSPYGVGYALSEFFRNLPNDPSLLPLLLYFFDVYRGFAEALSSLKNLQKLTLVSPYGVGYALSEFFRNLPNDPSLLPLLLYFFDVYRGFAEALSSLKNLQKLTLVSPYGVGYALSEFFRNLPND |
| HALC24-6_321 | MPNN | PNLLPLLKYFFDVWRGFAEALKSLKNLEYLDLDFPWGPGFALAVYVLNQPNNPNLLPLLKYFFDVWRGFAEALKSLKNLEYLDLDFPWGPGFALAVYVLNQPNNPNLLPLLKYFFDVWRGFAEALKSLKNLEYLDLDFPWGPGFALAVYVLNQPNNPNLLPLLKYFFDVWRGFAEALKSLKNLEYLDLDFPWGPGFALAVYVLNQPNN |
| HALC24-6_322 | MPNN | PNVLDLLKYFFDVFRGFAEALKSLKNLKYLDLYLPWGVGIALSVFLWHSPDNPNVLDLLKYFFDVFRGFAEALKSLKNLKYLDLYLPWGVGIALSVFLWHSPDNPNVLDLLKYFFDVFRGFAEALKSLKNLKYLDLYLPWGVGIALSVFLWHSPDNPNVLDLLKYFFDVFRGFAEALKSLKNLKYLDLYLPWGVGIALSVFLWHSPDN |
| HALC24-6_323 | MPNN | PERLPLLLEIFSVFRSIAEALSSLKNLKELYLYDPWGVGYALAELLRERPREPERLPLLLEIFSVFRSIAEALSSLKNLKELYLYDPWGVGYALAELLRERPREPERLPLLLEIFSVFRSIAEALSSLKNLKELYLYDPWGVGYALAELLRERPREPERLPLLLEIFSVFRSIAEALSSLKNLKELYLYDPWGVGYALAELLRERPRE |
| HALC24-6_324 | MPNN | PDLLPLILEFINVIRGFAEALKSLKNLKSLVLYDPYGPGLAISTLLTVLPDNPDLLPLILEFINVIRGFAEALKSLKNLKSLVLYDPYGPGLAISTLLTVLPDNPDLLPLILEFINVIRGFAEALKSLKNLKSLVLYDPYGPGLAISTLLTVLPDNPDLLPLILEFINVIRGFAEALKSLKNLKSLVLYDPYGPGLAISTLLTVLPDN |
| HALC24-6_325 | MPNN | PDLLPLLLELVDTIRGFAEGLRSLKNLQTLTLYFPWGPGYAVSEFLKELPDDPDLLPLLLELVDTIRGFAEGLRSLKNLQTLTLYFPWGPGYAVSEFLKELPDDPDLLPLLLELVDTIRGFAEGLRSLKNLQTLTLYFPWGPGYAVSEFLKELPDDPDLLPLLLELVDTIRGFAEGLRSLKNLQTLTLYFPWGPGYAVSEFLKELPDD |
| HALC24-6_326 | MPNN | PDLLELIKEFVDVIRKFGEALESLKNLKELTLIDPYGPGLAVAILVRELSNNPDLLELIKEFVDVIRKFGEALESLKNLKELTLIDPYGPGLAVAILVRELSNNPDLLELIKEFVDVIRKFGEALESLKNLKELTLIDPYGPGLAVAILVRELSNNPDLLELIKEFVDVIRKFGEALESLKNLKELTLIDPYGPGLAVAILVRELSNN |
| HALC24-6_327 | MPNN | DLPLEPLFQMLLSISNLSSALSTLKNLEHLELHSITGAGYAVSSLFESLKDKDLPLEPLFQMLLSISNLSSALSTLKNLEHLELHSITGAGYAVSSLFESLKDKDLPLEPLFQMLLSISNLSSALSTLKNLEHLELHSITGAGYAVSSLFESLKDKDLPLEPLFQMLLSISNLSSALSTLKNLEHLELHSITGAGYAVSSLFESLKDK |
| HALC24-6_328 | MPNN | EVLLPFLRAITESFRSLAEGISSLKNLQELQLNLDHIGYLGMTISELFKNNPPNPEVLLPFLRAITESFRSLAEGISSLKNLQELQLNLDHIGYLGMTISELFKNNPPNPEVLLPFLRAITESFRSLAEGISSLKNLQELQLNLDHIGYLGMTISELFKNNPPNPEVLLPFLRAITESFRSLAEGISSLKNLQELQLNLDHIGYLGMTISELFKNNPPNP |
| HALC24-6_329 | MPNN | SPDLPVYDTITQMFENIAKGISSLKNLKELSLNLPSPSGIFIGVGLYIIFNPSIKSPDLPVYDTITQMFENIAKGISSLKNLKELSLNLPSPSGIFIGVGLYIIFNPSIKSPDLPVYDTITQMFENIAKGISSLKNLKELSLNLPSPSGIFIGVGLYIIFNPSIKSPDLPVYDTITQMFENIAKGISSLKNLKELSLNLPSPSGIFIGVGLYIIFNPSIK |
| HALC24-6_330 | MPNN | SPDLPALEMLTKMFEALAKGLSSLKNLKELHLELPSPSGVFLGFGLYLIFNPNITSPDLPALEMLTKMFEALAKGLSSLKNLKELHLELPSPSGVFLGFGLYLIFNPNITSPDLPALEMLTKMFEALAKGLSSLKNLKELHLELPSPSGVFLGFGLYLIFNPNITSPDLPALEMLTKMFEALAKGLSSLKNLKELHLELPSPSGVFLGFGLYLIFNPNIT |
| HALC24-6_331 | MPNN | SPDLPALDVIREMFEALAQGLSSLKNLKELELRLPSPGGVGLAYGIYLTFNPSITSPDLPALDVIREMFEALAQGLSSLKNLKELELRLPSPGGVGLAYGIYLTFNPSITSPDLPALDVIREMFEALAQGLSSLKNLKELELRLPSPGGVGLAYGIYLTFNPSITSPDLPALDVIREMFEALAQGLSSLKNLKELELRLPSPGGVGLAYGIYLTFNPSIT |
| HALC24-6_332 | MPNN | SPDTPTFDVIRDMYKNLADGLAKLKNLKTLNLKLPSPGGVFIGIGLFMIFNPRITSPDTPTFDVIRDMYKNLADGLAKLKNLKTLNLKLPSPGGVFIGIGLFMIFNPRITSPDTPTFDVIRDMYKNLADGLAKLKNLKTLNLKLPSPGGVFIGIGLFMIFNPRITSPDTPTFDVIRDMYKNLADGLAKLKNLKTLNLKLPSPGGVFIGIGLFMIFNPRIT |
| HALC24-6_333 | MPNN | SPDTPAYDMLVEIYKELAKGFSHLPNLERLDLRLPSPSGVFIGFGLYLIFNPSIKSPDTPAYDMLVEIYKELAKGFSHLPNLERLDLRLPSPSGVFIGFGLYLIFNPSIKSPDTPAYDMLVEIYKELAKGFSHLPNLERLDLRLPSPSGVFIGFGLYLIFNPSIKSPDTPAYDMLVEIYKELAKGFSHLPNLERLDLRLPSPSGVFIGFGLYLIFNPSIK |
| HALC24-6_334 | MPNN | DVKSPLHETVVEIYKLLAESFKNLSKLKELDLTLPSPFGAALGLGLAYFLDPSVHDVKSPLHETVVEIYKLLAESFKNLSKLKELDLTLPSPFGAALGLGLAYFLDPSVHDVKSPLHETVVEIYKLLAESFKNLSKLKELDLTLPSPFGAALGLGLAYFLDPSVHDVKSPLHETVVEIYKLLAESFKNLSKLKELDLTLPSPFGAALGLGLAYFLDPSVH |
| HALC24-6_335 | MPNN | DKKTPAHDTIVDIYRLIAEGFKYLKDLKELRLELPSPYGAALGLGLAIFLNPSLSDKKTPAHDTIVDIYRLIAEGFKYLKDLKELRLELPSPYGAALGLGLAIFLNPSLSDKKTPAHDTIVDIYRLIAEGFKYLKDLKELRLELPSPYGAALGLGLAIFLNPSLSDKKTPAHDTIVDIYRLIAEGFKYLKDLKELRLELPSPYGAALGLGLAIFLNPSLS |
| HALC24-6_336 | MPNN | SQETPAHRTILQVYQLIADSFSKLSGLKELVLDLPSVEGLSLGLGLAIYNDPSVLSQETPAHRTILQVYQLIADSFSKLSGLKELVLDLPSVEGLSLGLGLAIYNDPSVLSQETPAHRTILQVYQLIADSFSKLSGLKELVLDLPSVEGLSLGLGLAIYNDPSVLSQETPAHRTILQVYQLIADSFSKLSGLKELVLDLPSVEGLSLGLGLAIYNDPSVL |
| HALC24-6_337 | MPNN | TEEPPALRALVSVYREIREAFEKLKNLKELHLRLPSVDGLLLGFGLALVRDPSLFTEEPPALRALVSVYREIREAFEKLKNLKELHLRLPSVDGLLLGFGLALVRDPSLFTEEPPALRALVSVYREIREAFEKLKNLKELHLRLPSVDGLLLGFGLALVRDPSLFTEEPPALRALVSVYREIREAFEKLKNLKELHLRLPSVDGLLLGFGLALVRDPSLF |
| HALC24-6_338 | MPNN | DKPPPAVEALISIFEKIRESFKYLSNLEELHLSLPSVDGLLLGYGLLLVKHPELLDKPPPAVEALISIFEKIRESFKYLSNLEELHLSLPSVDGLLLGYGLLLVKHPELLDKPPPAVEALISIFEKIRESFKYLSNLEELHLSLPSVDGLLLGYGLLLVKHPELLDKPPPAVEALISIFEKIRESFKYLSNLEELHLSLPSVDGLLLGYGLLLVKHPELL |
| HALC24-8_339 | MPNN | VEKLGISRETLEGIVQKLLDENDPEYLKLMLEILKAGTSVEKLGISRETLEGIVQKLLDENDPEYLKLMLEILKAGTSVEKLGISRETLEGIVQKLLDENDPEYLKLMLEILKAGTS |
| HALC25-5_340 | AF2 | NVTLPASFDLATAYQGIGEGLASLKDLRVLHLSVVFRLGGSNVTLPASFDLATAYQGIGEGLASLKDLRVLHLSVVFRLGGSNVTLPASFDLATAYQGIGEGLASLKDLRVLHLSVVFRLGGSNVTLPASFDLATAYQGIGEGLASLKDLRVLHLSVVFRLGGSNVTLPASFDLATAYQGIGEGLASLKDLRVLHLSVVFRLGGS |
| HALC25-5_341 | AF2 | EGAAIAENLATAYQGIGETLPSLQDLRVLHLSVIFSAEGSSPEGAAIAENLATAYQGIGETLPSLQDLRVLHLSVIFSAEGSSPEGAAIAENLATAYQGIGETLPSLQDLRVLHLSVIFSAEGSSPEGAAIAENLATAYQGIGETLPSLQDLRVLHLSVIFSAEGSSPEGAAIAENLATAYQGIGETLPSLQDLRVLHLSVIFSAEGSSP |
| HALC25-5_342 | AF2 | TGMAAFAENLATAYQGIGETLPSLKDLRVLHLSVLFYVYSGSSPTGMAAFAENLATAYQGIGETLPSLKDLRVLHLSVLFYVYSGSSPTGMAAFAENLATAYQGIGETLPSLKDLRVLHLSVLFYVYSGSSPTGMAAFAENLATAYQGIGETLPSLKDLRVLHLSVLFYVYSGSSPTGMAAFAENLATAYQGIGETLPSLKDLRVLHLSVLFYVYSGSS |
| HALC33-3_343 | MPNN | PSNWPEVAKYFDLGKALKPIGEGLQNLKNLKHLDLSFSFSLELYPGLPSNWPEVAKYFDLGKALKPIGEGLQNLKNLKHLDLSFSFSLELYPGLPSNWPEVAKYFDLGKALKPIGEGLQNLKNLKHLDLSFSFSLELYPGLPSNWPEVAKYFDLGKALKPIGEGLQNLKNLKHLDLSFSFSLELYPGLPSNWPEVAKYFDLGKALKPIGEGLQNLKNLKHLDLSFSFSLELYPGLPSNWPEVAKYFDLGKALKPIGEGLQNLKNLKHLDLSFSFSLELYPGLPSNWPEVAKYFDLGKALKPIGEGLQNLKNLKHLDLSFSFSLELYPGLPSNWPEVAKYFDLGKALKPIGEGLQNLKNLKHLDLSFSFSLELYPGLPSNWPEVAKYFDLGKALKPIGEGLQNLKNLKHLDLSFSFSLELYPGLPSNWPEVAKYFDLGKALKPIGEGLQNLKNLKHLDLSFSFSLELYPGLPSNWPEVAKYFDLGKALKPIGEGLQNLKNLKHLDLSFSFSLELYPGL |
| HALC33-3_344 | MPNN | WPEVAKSFDLGKGLEPIGKGLSSLKNLEHLNLTFNFNLEFYPGIPIDPKQWPEVAKSFDLGKGLEPIGKGLSSLKNLEHLNLTFNFNLEFYPGIPIDPKQWPEVAKSFDLGKGLEPIGKGLSSLKNLEHLNLTFNFNLEFYPGIPIDPKQWPEVAKSFDLGKGLEPIGKGLSSLKNLEHLNLTFNFNLEFYPGIPIDPKQWPEVAKSFDLGKGLEPIGKGLSSLKNLEHLNLTFNFNLEFYPGIPIDPKQWPEVAKSFDLGKGLEPIGKGLSSLKNLEHLNLTFNFNLEFYPGIPIDPKQWPEVAKSFDLGKGLEPIGKGLSSLKNLEHLNLTFNFNLEFYPGIPIDPKQWPEVAKSFDLGKGLEPIGKGLSSLKNLEHLNLTFNFNLEFYPGIPIDPKQWPEVAKSFDLGKGLEPIGKGLSSLKNLEHLNLTFNFNLEFYPGIPIDPKQWPEVAKSFDLGKGLEPIGKGLSSLKNLEHLNLTFNFNLEFYPGIPIDPKQWPEVAKSFDLGKGLEPIGKGLSSLKNLEHLNLTFNFNLEFYPGIPIDPKQ |
| HALC36-6_345 | MPNN | KVMSFEGDDLSTLFGGYGEGLKPLKRVAVLTLSVAFTMQAANTKVMSFEGDDLSTLFGGYGEGLKPLKRVAVLTLSVAFTMQAANTKVMSFEGDDLSTLFGGYGEGLKPLKRVAVLTLSVAFTMQAANTKVMSFEGDDLSTLFGGYGEGLKPLKRVAVLTLSVAFTMQAANTKVMSFEGDDLSTLFGGYGEGLKPLKRVAVLTLSVAFTMQAANTKVMSFEGDDLSTLFGGYGEGLKPLKRVAVLTLSVAFTMQAANT |
| HALC36-12_346 | MPNN | KVMSFEGDDLSTLFGGYGEGLKPLKRVAVLTLSVAFTMQAANTKVMSFEGDDLSTLFGGYGEGLKPLKRVAVLTLSVAFTMQAANTKVMSFEGDDLSTLFGGYGEGLKPLKRVAVLTLSVAFTMQAANT |
| HALC42-6_347 | MPNN | PSLTINDFGDIGKGLGEGLEGMKNLNELQLTITLTLTVSTPSLTINDFGDIGKGLGEGLEGMKNLNELQLTITLTLTVSTPSLTINDFGDIGKGLGEGLEGMKNLNELQLTITLTLTVSTPSLTINDFGDIGKGLGEGLEGMKNLNELQLTITLTLTVSTPSLTINDFGDIGKGLGEGLEGMKNLNELQLTITLTLTVSTPSLTINDFGDIGKGLGEGLEGMKNLNELQLTITLTLTVSTPSLTINDFGDIGKGLGEGLEGMKNLNELQLTITLTLTVST |
| HALC42-6_348 | MPNN | TSSPYIGIGLAEAFKAIGEGLKNLKYVKELQLTIVLPLEDIVPTSSPYIGIGLAEAFKAIGEGLKNLKYVKELQLTIVLPLEDIVPTSSPYIGIGLAEAFKAIGEGLKNLKYVKELQLTIVLPLEDIVPTSSPYIGIGLAEAFKAIGEGLKNLKYVKELQLTIVLPLEDIVPTSSPYIGIGLAEAFKAIGEGLKNLKYVKELQLTIVLPLEDIVPTSSPYIGIGLAEAFKAIGEGLKNLKYVKELQLTIVLPLEDIVPTSSPYIGIGLAEAFKAIGEGLKNLKYVKELQLTIVLPLEDIVP |
| HALC42-6_349 | MPNN | LLSPYIGIGLAEAFKAIGEGLKNLKYVKELQLEITLPLYDLVPLLSPYIGIGLAEAFKAIGEGLKNLKYVKELQLEITLPLYDLVPLLSPYIGIGLAEAFKAIGEGLKNLKYVKELQLEITLPLYDLVPLLSPYIGIGLAEAFKAIGEGLKNLKYVKELQLEITLPLYDLVPLLSPYIGIGLAEAFKAIGEGLKNLKYVKELQLEITLPLYDLVPLLSPYIGIGLAEAFKAIGEGLKNLKYVKELQLEITLPLYDLVPLLSPYIGIGLAEAFKAIGEGLKNLKYVKELQLEITLPLYDLVP |
| HALC42-6_350 | MPNN | LLSPYIGIGLAEAFKAIGEGLKNLKYVEQLELTIVLPLENVVPLLSPYIGIGLAEAFKAIGEGLKNLKYVEQLELTIVLPLENVVPLLSPYIGIGLAEAFKAIGEGLKNLKYVEQLELTIVLPLENVVPLLSPYIGIGLAEAFKAIGEGLKNLKYVEQLELTIVLPLENVVPLLSPYIGIGLAEAFKAIGEGLKNLKYVEQLELTIVLPLENVVPLLSPYIGIGLAEAFKAIGEGLKNLKYVEQLELTIVLPLENVVPLLSPYIGIGLAEAFKAIGEGLKNLKYVEQLELTIVLPLENVVP |
| HALC42-7_351 | MPNN | PSLTLNDFGDLGKGLGEGLQGMENLEKLQLTITLKLTVSTPSLTLNDFGDLGKGLGEGLQGMENLEKLQLTITLKLTVSTPSLTLNDFGDLGKGLGEGLQGMENLEKLQLTITLKLTVSTPSLTLNDFGDLGKGLGEGLQGMENLEKLQLTITLKLTVSTPSLTLNDFGDLGKGLGEGLQGMENLEKLQLTITLKLTVSTPSLTLNDFGDLGKGLGEGLQGMENLEKLQLTITLKLTVST |
| HALC42-7_352 | MPNN | LLSPYIGIGLAKAFKAIGEGLQNLKYVEELELTIVLPLEYIVPLLSPYIGIGLAKAFKAIGEGLQNLKYVEELELTIVLPLEYIVPLLSPYIGIGLAKAFKAIGEGLQNLKYVEELELTIVLPLEYIVPLLSPYIGIGLAKAFKAIGEGLQNLKYVEELELTIVLPLEYIVPLLSPYIGIGLAKAFKAIGEGLQNLKYVEELELTIVLPLEYIVPLLSPYIGIGLAKAFKAIGEGLQNLKYVEELELTIVLPLEYIVP |

**Table S4.** Designed sequences.

## **Supplementary References**

Citations
